# Supplementary material for: A general strategy to develop fluorogenic polymethine dyes for bioimaging
Source: Nat Chem. 2023 Nov 27;16(1):28–35. doi: 10.1038/s41557-023-01367-y (PMC10774129; doi:10.1038/s41557-023-01367-y)

nature chemistry

Article https://doi.org/10.1038/s41557-023-01367-y

A general strategy to develop fluorogenic polymethine dyes for bioimaging

In the format provided by the authors and unedited

Table of Contents

Supplementary Discussion 1 2

Supplementary Discussion 2 6

Supplementary Figures 9

Supplementary Tables 13

Synthetic procedures 15

Synthesis of building blocks *15*

Synthesis of 5-endo-trig carbocyanines *17*

Synthesis of 5-exo-trig carbocyanines *20*

Click reactions *31*

Supplementary Compounds *36*

References 41

NMR spectra 42

Plasmid maps 74

Supplementary Discussion 1

We have shown that compound **11**, a 5-*exo*-*trig* Cy5 with hydroxyethyl ring-closing moiety, has a very similar pK_a to HMSiR (5.7 for **11** vs 5.8 for HMSiR) and exhibits similar spontaneously blinking behavior.

Next, we investigated other ring-closing moieties used in fluorogenic rhodamines. We first opted for a carboxylic acid to generate tetramethylrhodamine (TAMRA)-like carbocyanines. We synthesized **S1** (Figure SD1), keeping the CF_3 substituent present in compound **11**. Probe **S1** has a D_{50} value of 13, which is very similar to TAMRA, but exhibits no pH dependence.

These results indicated that a more stable ring was needed to increase fluorogenicity, thus, we next tried amides as ring-closing moieties. We transformed methyl ester **9d** into an *N*-methyl amide, clicked it to SNAP-tag azide, and evaluated the resulting probe **S3** in vitro and in live-cell imaging experiments. Probe **S3** has a pK_a value below 2 and a closed form that is stable at all measured polarities. We tested compound **S3** in live HeLa cells transfected with H2B-SNAPf-mTurquoise2 and observed very low non-specific fluorescence signal, but also only weak turn-on fluorescence in the nucleus upon SNAP-tag binding. Guided by our computational modeling, we hypothesized that the CF_3 group shifts the equilibrium significantly towards the closed form. Its removal led to compound **15**, which we expected to be more prone to ring opening. Indeed, compound **15** shows a good compromise between a sufficiently stable closed form in solution (pK_a of 2.6 and no polarity dependence) with very low non-specific fluorescence signal, but a good turn-on upon binding to SNAP-tag.

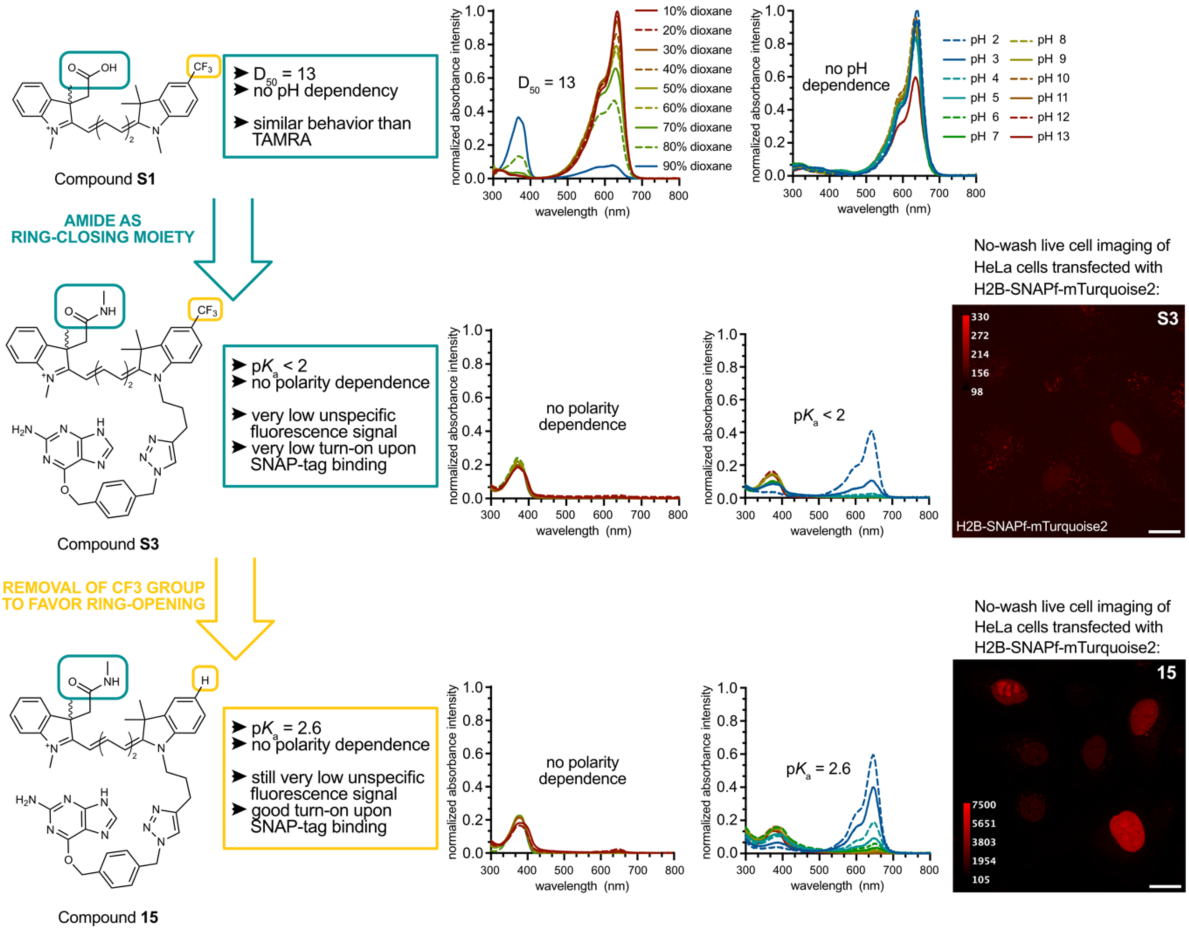

Figure SD1. Overview of the rational design of compound **15**.

The effect of the 5-*exo-trig* ring-closing strategy is clear when comparing the properties of compounds **15** and **16** (Figure SD2). 5-*endo*-trig compound **16** has a pK_a value of 9.4 (compound **15** has a pK_a value of 2.6) and remains in the open form regardless of polarity (compound **15** remains in the closed form regardless of polarity). Scale bar: 15 μm. Spectra are representative from three independent experiments and micrographs are representative of three independent biological samples measured over three separate imaging sessions.

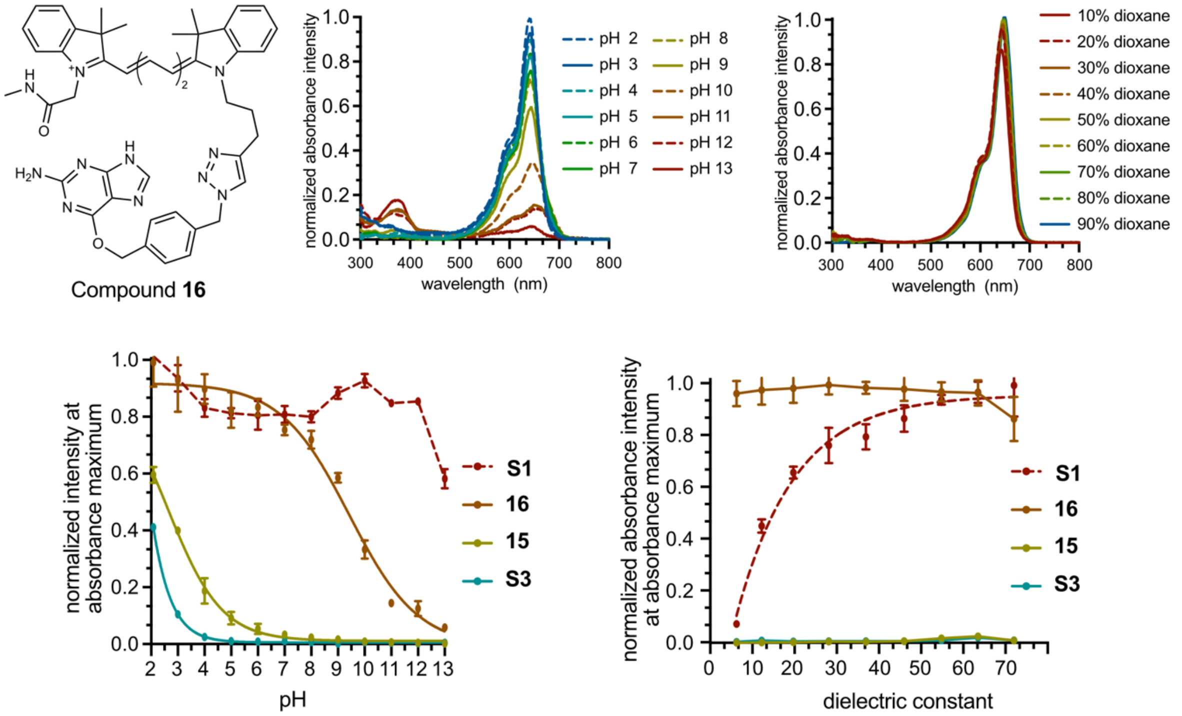

Figure SD2. pH and polarity profile of the corresponding 5-*endo-trig* compound **16** and summary of all Cy5 probes. Symbols indicate the mean of three independent experiments and error bars represent standard deviation. Spectra are representative from three independent experiments.

To design fluorogenic Cy3 and Cy7 derivatives, we suspected that the Cy3 derivative would have a higher LUMO energy, whereas the opposite would be true for the Cy7 derivative. Thus, we expected the Cy3 to have a higher tendency to exist in the open form than Cy5, and the Cy7 to have a higher tendency to exist in the closed form. Based on this assumption, we kept the CF_3 group for our Cy3 design resulting in compound **19** (Figure SD3). Compound **19** has a pK_a value of 3.7, which is higher than that of **15**, and its closed form is stable at all measured polarities. In live-cell imaging experiments, this compound still shows only minimal lysosomal background and a good fluorescence turn-on upon SNAP-tag binding. These results demonstrate that Cy3 derivatives indeed have a higher intrinsic tendency to remain in the open form and thus strongly nucleophilic ring-closing moieties and electron-withdrawing substituents are needed for fluorogenicity.

In the case of our Cy7 design, we synthesized **S6**, which has a methyl amide as nucleophile and no CF_3 group (Figure SD3). This compound has a closed form that is stable at all measured polarities and pH values. Compound **S6** displayed very low background in live-cell imaging, but also only a weak turn-on upon binding to SNAP-tag. Therefore, we decided to weaken the nucleophilicity of the ring-closing amide by introducing fluorine substituents, resulting in compound **20**. We note that an alternative strategy would be to introduce electron-donating substituents to the indolenine or polymethine chain.

Compound **20** has a pK_a value of 2.7 and a D_{50} value of approximately 52. It is noteworthy that the absorbance decreases in dioxane:water mixtures with >70% water content, possibly indicating aggregation in aqueous medium at the relatively high concentration of this experiment (10 µM). In live-cell imaging experiments, **20** showed a good compromise between low non-specific background and fluorescence turn-on upon SNAP-tag binding. Since live-cell experiment were carried out at a much lower concentration (50 nM), we do not expect aggregation to play a significant role in fluorogenicity.

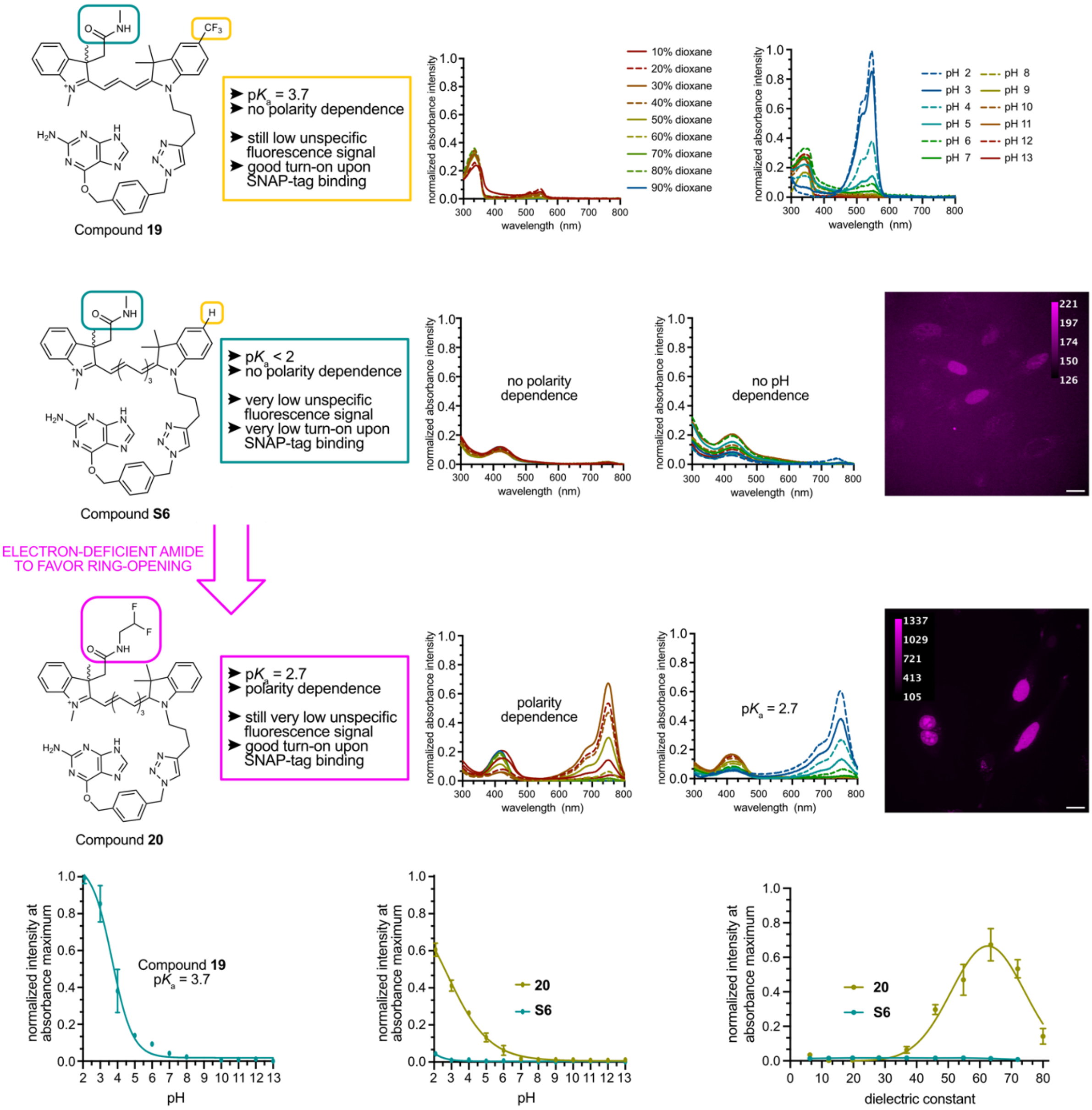

Figure SD3. Overview of the rational design of compounds **19** and **20**. In pH and polarity profiles, the symbols indicate the mean of three independent experiments and error bars represent standard deviation. Scale bars: 15 µm. Spectra are representative from three independent experiments and micrographs are representative of three independent biological samples measured over three separate imaging sessions

In summary, we illustrated that our 5-*exo-trig* ring-closing strategy is general and robust. The cyclization equilibrium can be fine-tuned by varying substituents on the capping indoleninium, as well as by modifying the ring-closing group. In general, 5-*exo-trig* dyes with pK_a values between 2.5 and 3.5 showed very low non-specific background in live HeLa cells and good turn-on upon SNAP-tag binding. Dyes with pK_a values below 2 showed only very little turn-on, and dyes with pK_a values above 4 are expected to show lysosomal background signal.
Further improvement of the dyes can be achieved by enhancing the photophysical properties of the dyes and optimizing the linker to the targeting moiety *via* chemical modifications, as well as by protein engineering to increase the interactions of the small molecule with the protein surface (see Supplementary Discussion 2) and to stabilize the open form.

Supplementary Discussion 2

We have shown that carbocyanines with a 5-*exo*-trig cyclization mechanism are largely present as non-fluorescent, cyclized forms in solution. In most cases, their pK_a of ring opening is rather low, and they are largely insensitive to changes in polarity. Naturally, the question of how they turn-on upon binding to their target arises. To provide preliminary insight into this process, we carried out molecular mechanics simulations of (*S*)-**15** and (*S*)-**18**-DNA bound to their targets.

Method
For (*S*)-**15**, conformational searches were carried out using the crystal structure of SNAP-tag attached to a rhodamine ligand (PDB: 6Y8P). All calculations were carried out with the modules of the Schrödinger package, 2023-1 release. The protein structure was loaded onto Maestro and the atoms of the ligand (rhodamine) were deleted up to the benzyl group that is covalently attached to C145. Water molecules that had contacts to the ligand but not to the protein were removed as well. Compound (*S*)-**15**, either in the open or closed form, was built in a conformation that avoided obvious steric clashes and the conjugated p-system was roughly planar. The protein conjugate was prepared using the Protein Preparation Workflow of Maestro. The simulation was set up at pH = 7.4 and crystallographic water molecules farther than 5 Å from heteroatoms were removed. Other solvents (ethylene glycol) were also removed, but the bound Zn atom was kept. Hydrogens and lone pairs were added to the structure, which was subsequently optimized using the OPLS4 force field using water as solvent. A conformational search was set up by designating the ligand as freely moving atoms. A first shell of restricted residues was defined after considering the possible conformations of the ligand and residues within a distance of 5.0 Å of any of those conformations were restricted using a force constant of 200.0 kJ mol^{-1} $Å^{-2}$. This shell included residues E30, I32, F33, L34, G35, K36, H86, P87, V88, T95, K131, T132, A133, L134, S135, G136, N136, P138, P140, C145, H146, G157, Y158, E159, G160, G161, L162, and V164. All atoms in the protein further away from this shell were frozen. The conformational space of the ligand was searched using the torsional

sampling method (MCMM), taking 50 steps per rotational bond and saving structures within 10 kJ mol^{-1} of the global minimum. The energy minimization of each structure was carried out using the OPLS4 force field, with water as solvent, using charges from force field and an extended cutoff. Minimization was carried out using the Polak-Ribier Conjugate Gradient method with 5000 maximum iterations and a converge threshold of 0.05 on gradient. Conformational searches were carried out equivalently for **18**-DNA bound to DNA using the crystal structure of Hoechst 33258 bound to dodecamer CGCGAATTCGCG (PDB: 1DNH).

Results

The closed form of (*S*)-**15** binds to SNAP-tag in a way that the cyclic indolenine is positioned over the β-sheet formed between residues H29 and G35. About 10 conformations were found within 2 kcal mol^{-1} of the global minimum, all of which were oriented in a similar way, but with different rotations around the bond that joins the cyclized indolenine with the polymethine chain (Figure SD4a). This distribution of conformers suggests that the closed indolenine can explore several binding poses, but the polymethine chain is relatively rigid. In one of the binding poses, we observed that the carbonyl of the lactam that cyclizes the indolenine forms a hydrogen bond with the backbone amide of K36, with a distance O–H = 1.93 Å (Figure SD4a, inset). An equivalent conformational search for the open form revealed that the open amide (formerly a lactam in the closed form) also forms a hydrogen bond to the backbone amide of K36, but with a shorter distance O–H = 1.75 Å (Figure SD4b), suggesting a stronger interaction that could be the driving force for ring opening. These results, albeit preliminary, suggest that specific interactions (e.g., hydrogen bonding) could be responsible for ring-opening in 5-*exo-trig* carbocyanines once that they are tightly bound to a macromolecule.

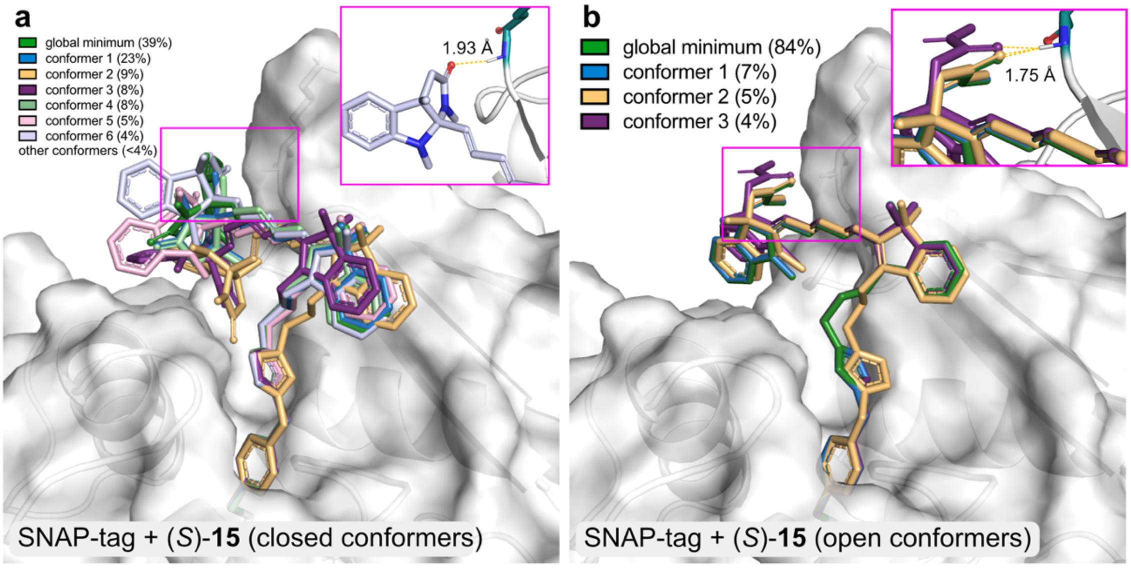

Figure SD4. Calculated low-energy conformers of compound (*S*)-**15** bound to SNAP-tag. a) Ensemble of conformers of the closed (a) and open (b) forms of compound (*S*)-**15** bound to SNAP-tag.

To investigate whether such interactions were also observed in other macromolecules, we performed an equivalent conformational search of (*S*)-**18**-DNA bound to dsDNA. As mentioned before, the starting point for this calculation was the crystal structure of Hoechst 33258 bound to dodecamer CGCGAATTCGCG (PDB: 1DNH).

In this case, all conformations found within 2 kcal mol^{-1} of the global minimum were identical both for the open and closed forms. The closed form adopted a pose in which the lactam of the cyclized indolenine point out into the solvent and does not have any specific interactions with DNA (Figure SD5a, pale cyan structure). In contrast, in the open form, the acyclic amide can form a hydrogen bond with a phosphate of the DNA backbone (Figure SD5a, magenta structure). The O–H distance of this interaction is 1.80 Å (Figure SD5b). Similar to what was observed for (*S*)-**15** and SNAP-tag, a specific interaction (hydrogen bonding) seems to be plausible way in which the open, fluorescent, form of these molecules is stabilized upon tight binding to a macromolecular target.

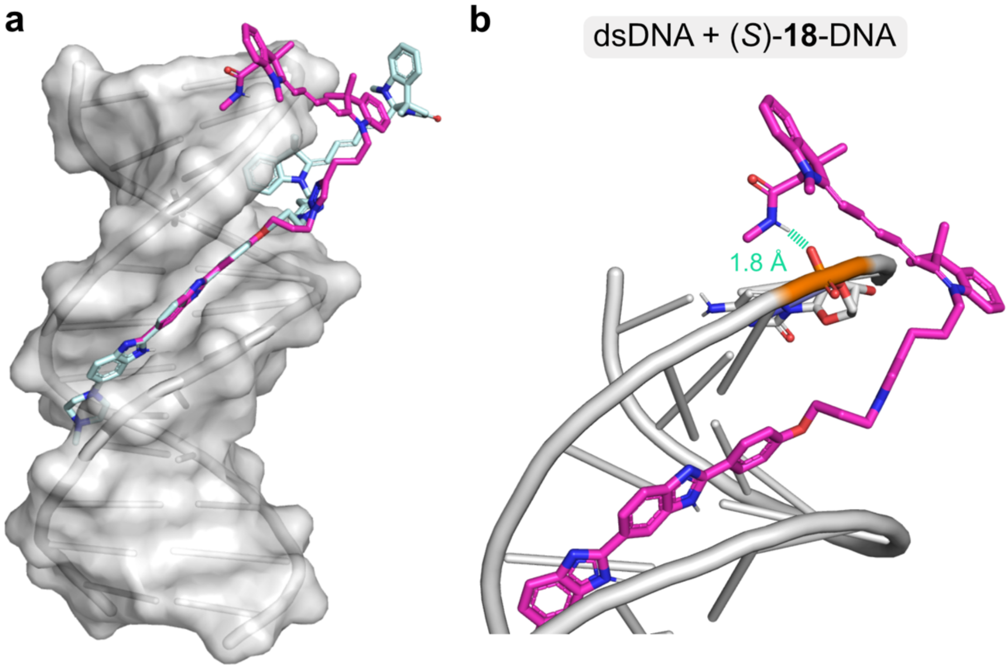

Figure SD5. Structures of (*S*)-**18**-DNA bound to dsDNA. a) Conformations of the global minima calculated for the open (magenta) and closed (pale cyan) forms of (*S*)-**18**-DNA bound to dsDNA. b) Hydrogen bonding between the acyclic amide in the open form of (*S*)-**18**-DNA and a backbone phosphate group in DNA.

Clearly, these results are only preliminary and a future investigation of the mechanism of fluorogenicity would require full molecular dynamics simulations with explicit solvent molecules, quantum mechanics/molecular mechanics (QM/MM) calculations of the ring-opening process, and experimental mutagenesis experiments to probe the importance of the interactions identified computationally.

Supplementary Figures

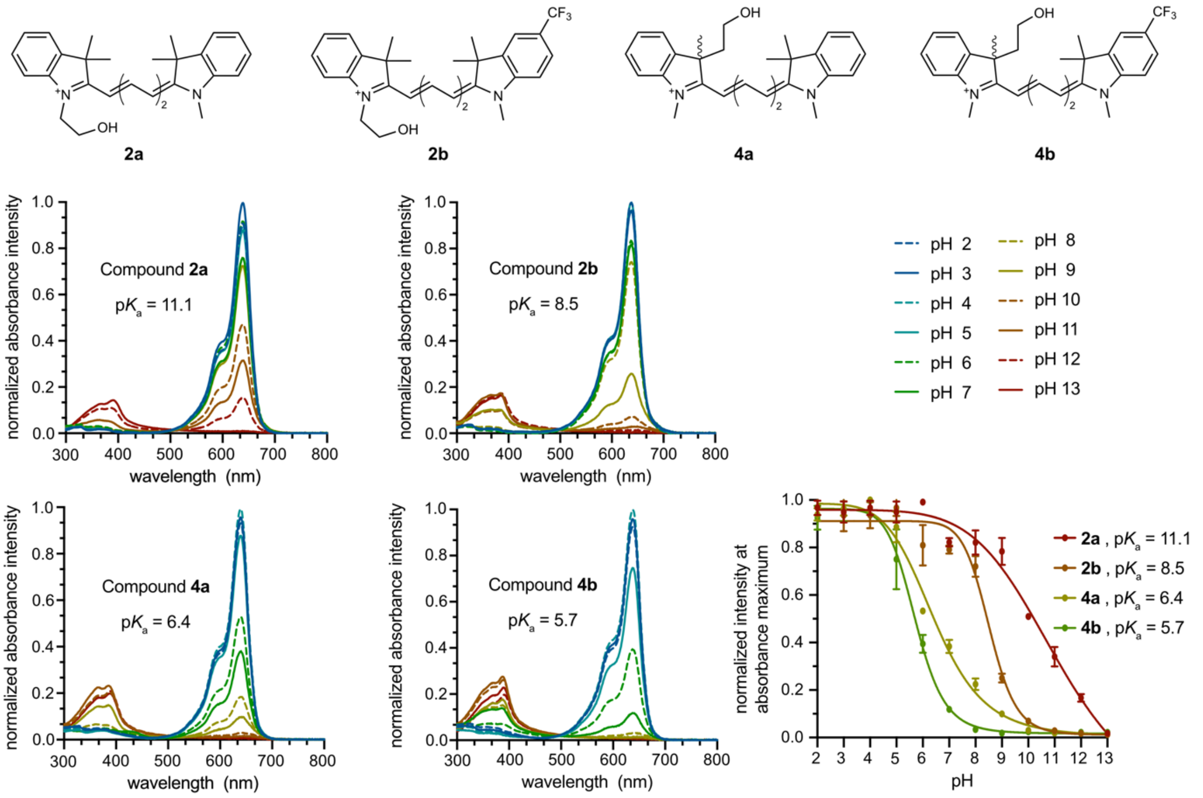

Figure 1. pH profiles of compounds with an alcohol as ring-closing moiety. Symbols indicate the mean of three independent experiments and error bars represent standard deviation. Spectra are representative from three independent experiments.

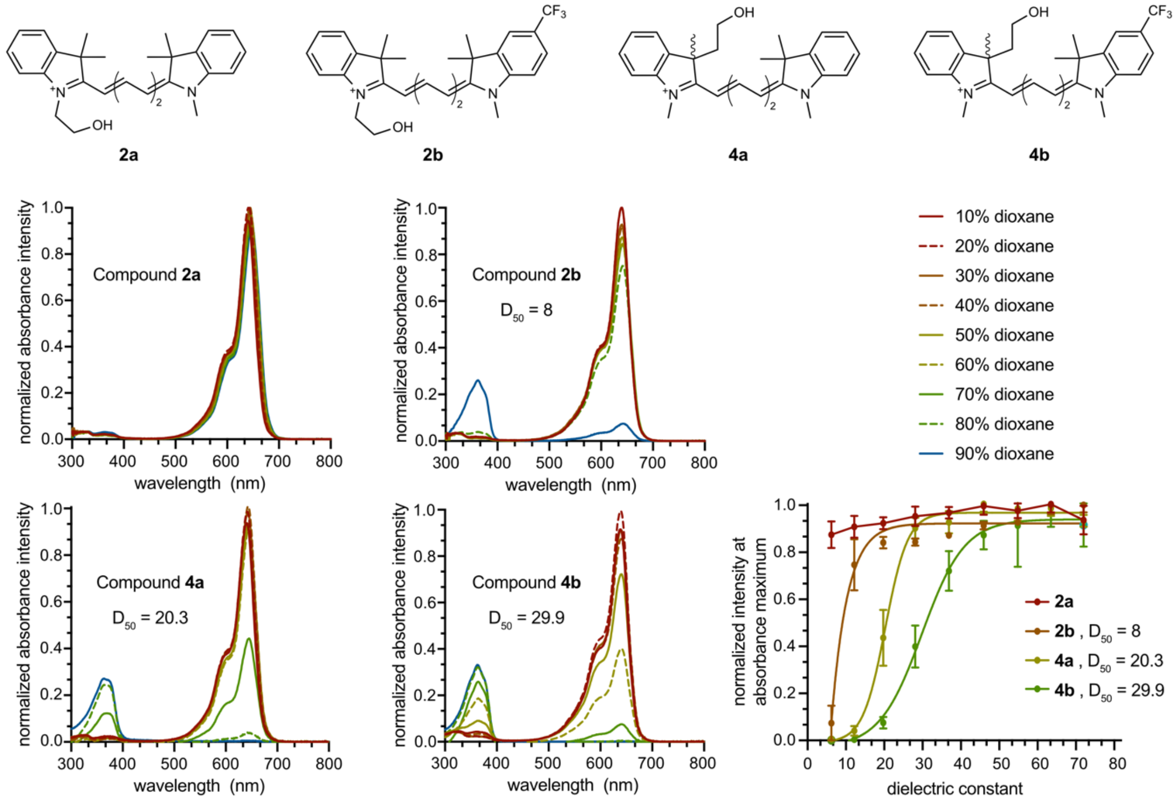

Figure 2. polarity profiles of compounds with an alcohol as ring-closing moiety. Symbols indicate the mean of three independent experiments and error bars represent standard deviation. Spectra are representative from three independent experiments.

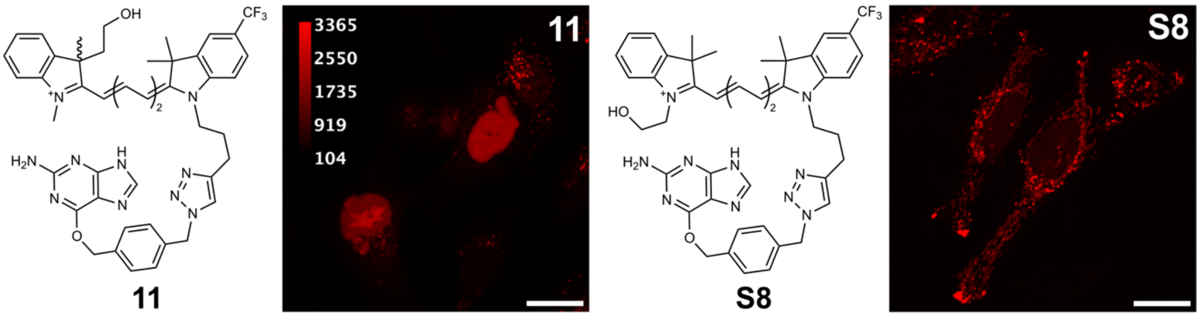

Figure 3. Comparison of cellular uptake of compounds 5-*exo* trig and 5-*endo-trig* compounds. No-wash live-cell imaging of HeLa cells transfected with H2B-SNAPf-mTurquoise2 and incubated with **11** (50 nM) or **S8** (50 nM). Since probe **11** has a pK_a value of 5.7 and compound S8 has a pK_a value of 8.5, their similar fluorescence intensities in acidic vesicles (pH of 4–5) suggest comparable cellular uptake. Scale bar = 15 μm. Micrographs are representative of three independent biological samples measured over three separate imaging sessions.

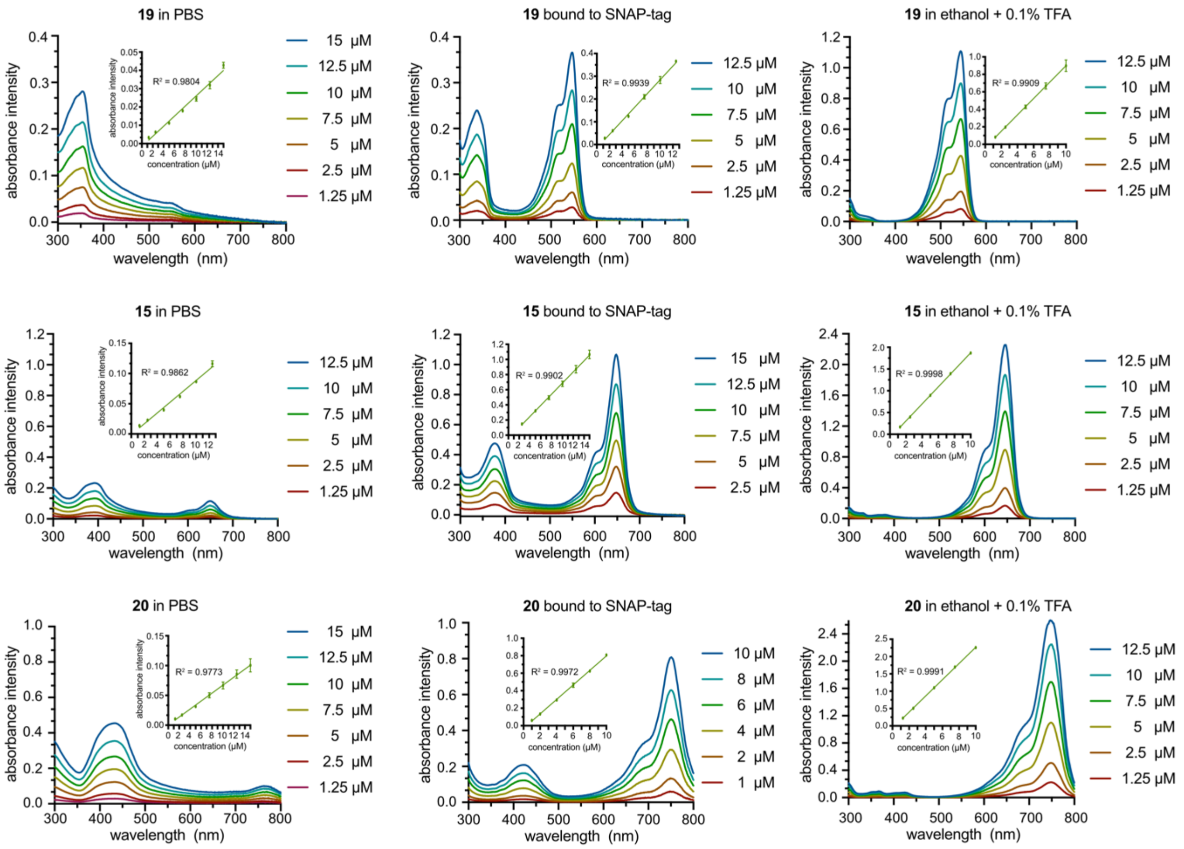

Figure 4. Full absorbance spectra of extinction coefficient measurements of **15**, **19** and **20** in PBS, bound to SNAP-tag protein and in ethanol + 0.1% TFA. Spectra are representative from three independent experiments.

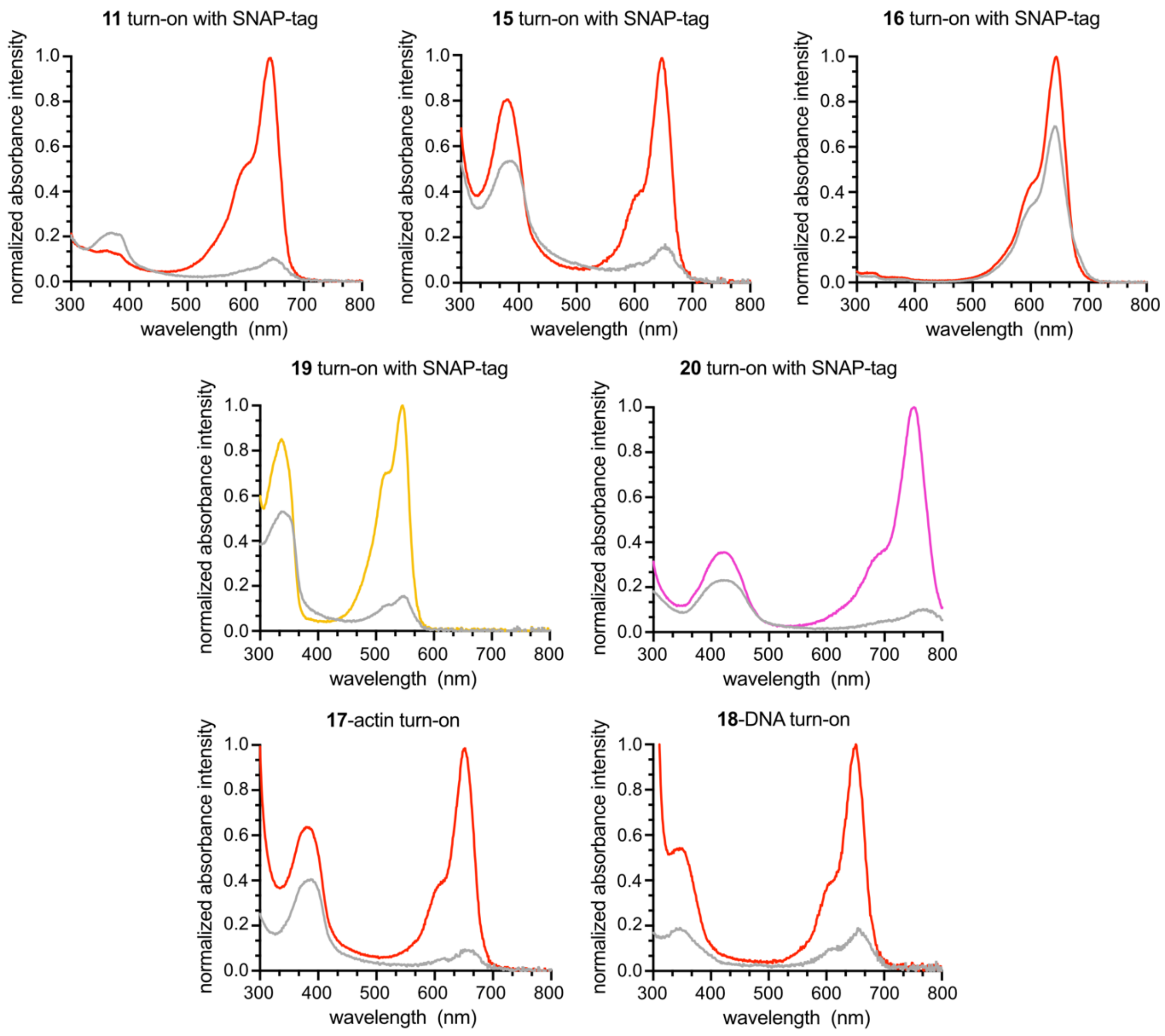

Figure 5. Absorbance spectra of **11**, **15**, **16**, **19** and **20** with SNAP-tag protein, **17**-actin with actin and **18**-DNA with ds-DNA in the range 300–800 nm. Spectra are representative of three independent experiments. Grey lines indicate absorbance of free dye and colored lines indicate absorbance of the dye-macromolecule conjugate.

Supplementary Tables

Table 1. Calculated energies of the open and closed form of **1**, **2**, **3, 4a**, **4b** and of the transition state of ring closure using DFT theory, B3LYP or M06-2X functional and the DGTZVP basis set. Energy values are reported in kcal mol^{-1}.

Probe	closed form	open form	transition state
1 (B3LYP)	-930591.73	-930566.39	-930563.39
2 (B3LYP)	-797468.7184	-797455.0657	-797449.7312
3 (B3LYP)	-822124.75	-822108.05	-822102.69
4a (B3LYP)	-797472.89	-797446.15	-797441.7
4b (B3LYP)	-1009053.449	-1009023.302	-1009020.434
1 (M06-2X)	-930256.9573	-930219.9555	-930216.4001
2 (M06-2X)	-797134.25	-797104.38	-797103.34
3 (M06-2X)	-821779.50	-821747.81	-821745.36*
4a (M06-2X)	-797137.69	-797096.93	-797094.28
4b (M06-2X)	-1008650.22	-1008608.32	-1008605.57

*For the calculation of this transition state, the following Gaussian command was used: opt=(calcfc,ts,noeigentest)

Table 2. Plasmid sources.

Plasmid	Source	Vector precursor	Insert precursor
pET24b-6His-fSNAP	Addgene #106999		
pSNAPf-H2B	Addgene #101124		
pEBTet-β-tubulin_SNAP	Addgene #136831		
pmTurquoise2-Golgi	Addgene #36205		
pmTurquoise2-ER	Addgene #36204		
pmTurquoise2-Mito	Addgene #36208		
LifeAct-Halo-T2A-eGFP	Addgene #135445		
TOM20-Halo-T2A-eGFP	Addgene #135443		
TUBB5-Halo	Addgene #64691		
SNAPf-mTurquoise2	Gibson assembly[1]	pmTurquoise2-Golgi	pSNAPf-H2B
H2B-SNAPf-mTurquoise2	Gibson assembly[1]	pmTurquoise2-Golgi	pSNAPf-H2B
SNAPf-mTurquoise2-KDEL	Gibson assembly[1]	pmTurquoise2-ER	pSNAPf-H2B
LifeAct-SNAPf-mTurquoise2	Gibson assembly[1]	H2B-SNAPf-mTurquoise2	LifeAct-HT7-T2A-eGFP
TOM20-SNAPf-mTurquoise2	Gibson assembly[1]	H2B-SNAPf-mTurquoise2	TOM20-HT7-T2A-eGFP

Table 3. List of primers for Gibson Assembly.

Plasmid	backbone forward primer	backbone reverse primer	fragment forward primer	fragment reverse primer
SNAPf-mTurquoise2	GAGGT TAATG TGAGC AAGGGC	TTTGT CCATT GA GTC CGGTA GCG	CGGAC TCAAT GGACA AAGAC TGC	CCTTG CTCAC ATTAA CCTCG AG
H2B-SNAPf-mTurquoise2	GAGGT TAATG TGAGC AAGGGC	TCTGG CATTG AGTCC GGTAGC	CGGAC TCAAT GCCAG AGCCA	CCTTG CTCAC A TTAA CCTCGAG
SNAPf-mTurquoise2-KDEL	GAGGT TAATG TGAGC AAGGGC	GTCTT TGTCC ATGGT GGCGAC	CCACC ATGGA CAAAG ACTGC	CCTTG CTCAC ATTAA CCTCG AG
LifeAct-SNAPf-mTurquoise2	CGGTA CTGCG CCAGC ATTT	CCACG CCCAT TGAGT CCGG	CTCAA TGGGC GTGGC CGAC	GCTGG CGCAG TACCG ATTTCG
TOM20-SNAPf-mTurquoise2	TGTGG AAGCG CCAGC ATTT	CCCAC CATTG AGTCC GGTAG	GGACT CAATG GTGGG TCGG	GCTGG CGCTT CCACA TCATCT

Table 4. Characterization of SNAP-conjugated fluorogenic dyes.

SNAP-dye	$\lambda_{ex}/\lambda_{em}$	ε [$\times$ 10^4 $M^{-1}cm^{-1}$]	ϕ
Cy3-SNAP **19**	545/567	0.3^a/ 3.0^b/ 9.4^c	$—^a$/0.16^b/0.04^c
Cy5-SNAP **15**	644/672	0.9^a/ 7.3^b/ 19.6^c	0.16^a/0.46^b/0.28^c
Cy7-SNAP **20**	749/784	0.7^a/ 8.3^b/ 23.4^c	0.03^a/0.33^b/0.16^c

a: PBS, b: bound to SNAP-tag protein in PBS, c: ethanol + 0.1% TFA

Table 5. Microscope settings for imaging channels.

Channel	λ_{ex}	Emission filter	Laser intensity	Exposure time
mTurquoise2	445 nm	472/30	30 mW	300 ms
Cy3, JF549	561 nm	600/52	23 mW	300 ms
Cy5, JF646	638 nm	708/75	25 mW	400 ms
Cy7	638 nm	647LP	25 mW	400 ms

Synthetic procedures

Synthesis of building blocks

Building blocks **5**, **7a**, **7b**, **8** and **13** were prepared according to previously reported methods.[2–4]

3-(Carboxymethyl)-1,2,3-trimethyl-3*H*-indol-1-ium bromide[2] (6)

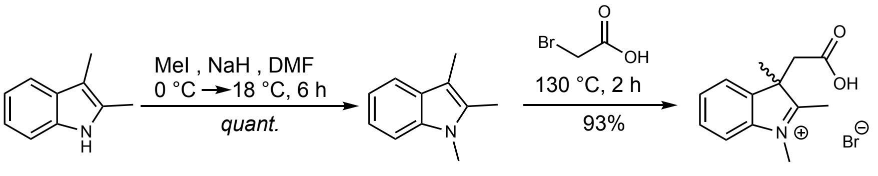

6

Sodium hydride (60% dispersion in mineral oil, 7.3 g, 183 mmol, 2.6 equiv.) was added to a flame-dried round-bottom flask, cooled to 0 °C and carefully dissolved in DMF (50 mL). 2,3-dimethylindole (10 g, 68.9 mmol, 1.0 equiv.) was added portion-wise at 0 °C followed by the addition of iodomethane (5.6 mL, 89.5 mmol, 1.3 equiv.). After stirring at 0 °C for 1 h, the suspension was warmed to room temperature and stirred for 4 h. The mixture was cooled to 0 °C and cold water (25 mL) was added dropwise. The mixture was extracted twice with ethyl acetate and the organic phase was washed with brine, dried over Na_2SO_4 and filtered. Solvents were removed under reduced pressure to afford 1,2,3-trimethyl-1*H*-indole as a dark orange oil, which was used directly without further purification.

Bromoacetic acid (28.8 g, 207 mmol, 3.0 equiv.) was slowly melted in a flame-dried flask (45-50 °C) under argon atmosphere. 1,2,3-trimethyl-1*H*-indole (11 g, 69.1 mmol, 1.0 equiv.) was added and the mixture was heated to 130 °C for 2 h. The mixture was slowly cooled to 18 °C and the residue was washed twice with diethyl ether and CH_2Cl_2. Acetonitrile was added and the suspension was left in the fridge for 1 h. The beige precipitate was collected by filtration and washed with diethyl ether to afford the product as a beige solid (19.1 g, 93%). The racemic mixture was then separated on a ChiralPak IJ column using 100% EtOH + 0.1% FA as mobile phase.

(*R*)-(+)-6: $[\alpha]_{589}^{296K}$ = +74° ± 2°, (c = 6.0 mg mL^{-1}, CH_3OH).
(*S*)-(–)-6: $[\alpha]_{589}^{297K}$ = –64° ± 3°, (c = 6.0 mg mL^{-1}, CH_3OH).
^{1}H NMR (400 MHz, CD_3OD): δ = 7.83 (dd, *J* = 6.6, 2.5 Hz, 1H), 7.79 – 7.77 (m, 1H), 7.68 – 7.61 (m, 2H), 4.07 (s, 3H), 3.58 (d, *J* = 17.5 Hz, 1H), 3.50 (d, *J* = 17.5 Hz, 1H), 1.56 (s, 3H) ppm.
$^{13}C\{^1H\}$ NMR (101 MHz, CD_3OD): δ = 196.1, 171.2, 143.0, 139.5, 129.5, 129.4, 122.9, 114.67, 55.6, 40.9, 33.6, 20.7 ppm.
HRMS (ESI) calcd for $[C_{13}H_{16}NO_2]^+$: 218.11810, found 218.11770.

2,3,3-Trimethyl-1-(pent-4-yn-1-yl)-3*H*-indol-1-ium iodide (7c)

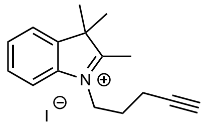

5-iodo-pent-1-yne was prepared according to previously reported methods.[5] 2,3,3-trimethylindolenine (200 μL, 1.26 mmol, 1.0 equiv.) and 5-iodo-pent-1-yne (731 mg, 3.77 mmol, 3.0 equiv.) were dissolved in CH_3CN (5 mL) and heated under reflux for 16 h. Solvents were removed under reduced pressure and the residue was washed with diethyl ether multiple times to yield a pale pink solid (389 mg, 88%).

^{1}H NMR (400 MHz, $(CD_3)_2SO$): δ = 7.99 – 7.96 (m, 1H), 7.87 – 7.84 (m, 1H), 7.64 – 7.62 (m, 2H), 4.51 (t, *J* = 7.7 Hz, 2H), 2.95 (t, *J* = 2.7 Hz, 1H), 2.86 (s, 3H), 2.43 (td, *J* = 7.2, 2.7 Hz, 2H), 2.07 (p, *J* = 7.2 Hz, 2H), 1.55 (s, 6H) ppm.
^{13}C{^{1}H} NMR (101 MHz, $(CD_3)_2SO$): δ = 197.0, 141.8, 141.1, 129.4, 128.9, 123.5, 115.2, 83.1, 72.4, 54.3, 46.8, 26.0, 22.0, 15.2, 14.2 ppm.
HRMS (ESI) calcd for $[C_{16}H_{20}N]^+$: 226.15958, found 226.15904.

2,3,3-Trimethyl-1-(pent-4-yn-1-yl)-5-(trifluoromethyl)-3*H*-indol-1-ium iodide (7d)

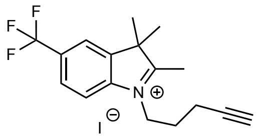

2,3,3-trimethyl-5-(trifluoromethyl)-*3H*-indole was prepared according to previously reported methods.[6] 2,3,3-trimethyl-5-(trifluoromethyl)-*3H*-indole (850 mg, 3.74 mmol, 1.0 equiv.) and 5-iodo-pent-1-yne (1.81 g, 9.35 mmol, 2.5 equiv.) were dissolved in CH_3CN (10 mL) and heated under reflux for 36 h. Solvents were removed under reduced pressure and the residue was washed with diethyl ether multiple times to yield the product as a pale purple solid (580 mg, 37%).

^{1}H NMR (400 MHz, CD_3CN): δ = 8.08 (s, 1H), 7.98 – 7.93 (m, 2H), 4.48 (t, *J* = 7.8 Hz, 2H), 2.81 (s, 3H), 2.42 (td, *J* = 6.9, 2.7 Hz, 2H), 2.35 (t, *J* = 2.7 Hz, 1H), 2.14 (p, *J* = 7.3 Hz, 2H), 1.59 (s, 6H).
^{13}C{^{1}H} NMR (101 MHz, CD_3CN): δ = 160.3 (q, *J* = 37.1 Hz), 145.0, 143.8, 127.9 (q, 3.8 Hz), 126.2, 123.5, 122.1 (q, 3.8 Hz), 117.2, 115,6, 83.2, 71.8, 56.3, 48.5, 27.0, 22.5, 16.3.
HRMS (ESI) calcd for $[C_{17}H_{19}F_3N]^+$: 294.14696, found 294.14606.

6-((4-(Aminomethyl)benzyl)oxy)-9*H*-purin-2-amine-6-((4-(azidomethyl)benzyl)-oxy)-9*H*-purin-2-amine (10)

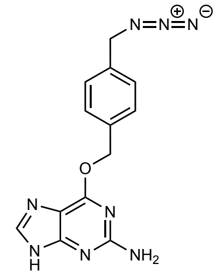

SNAP-amine[7] and FSO_2N_3[8] were prepared according to previously reported methods. SNAP-amine (30 mg, 0.11 mmol, 1.0 equiv.) was dissolved in DMF/MTBE/H_2O (94:5:1, 1.5 mL) and FSO_2N_3 solution (1.35 equiv., 307 μL of a 488 mM solution) and aqueous potassium hydrogen carbonate solution (3 M, 1.5 mL) were added. The mixture was stirred for 3 h at 18 °C. Ethyl acetate was added and the organic phase was washed trice with brine, once with water and again with brine. The extracted organic phase was dried over Na_2SO_4, filtered, and solvents were removed under reduced pressure to yield the product as an off-white solid (32 mg, 97%).

^{1}H NMR (400 MHz, $(CD_3)_2SO$): δ = 12.43 (s, 1H), 7.82 (s, 1H), 7.54 (d, *J* = 8.1 Hz, 2H), 7.40 (d, *J* = 8.2 Hz, 2H), 6.29 (s, 2H), 5.49 (s, 2H), 4.46 (s, 2H) ppm.
^{13}C{^{1}H} NMR (101 MHz, $(CD_3)_2SO$): δ = 159.6, 136.7, 135.4, 129.0, 128.7, 128.54, 128.50, 128.4, 66.3, 53.3 ppm.
HRMS (ESI) calcd for $[C_{13}H_{12}N_8O+H]^+$: 297.12068, found 297.12043.

Synthesis of 5-*endo*-*trig* carbocyanines

General procedure to synthesize 5-*endo*-*trig* hydroxyethyl probes 2a and 2b

Compound **5** (50 mg, 0.15 mmol, 1.0 equiv.) and **8** (45 mg, 0.18 mmol, 1.2 equiv.) were dissolved in Ac_2O (1 mL) in a microwave vial. The solution was heated to 120 °C for 1 h in the microwave. The mixture was cooled to room temperature and a solution of **7** (0.17 mmol, 1.1 equiv.) in pyridine (1 mL) was added dropwise. The mixture was stirred for 30 min at 18 °C. The color of the solution turned from green to blue. Solvents were removed under reduced pressure. The residue was washed with diethyl ether and then stirred in a mixture of CH_3OH (2 mL) and saturated K_2CO_3 (3 mL) for 2 h at 18 °C to deprotect the acetylated alcohol. Solvents were removed under reduced pressure and the residue was dissolved in CH_2Cl_2 and washed three times with water and twice with brine. The organic layer was dried over Na_2SO_4, filtered, and purified via reverse-phase column chromatography (10 → 70% CH_3CN in ddH_2O + 0.1% TFA over 35 min) to afford the product as a fine blue powder.

1-(2-Hydroxyethyl)-3,3-dimethyl-2-((1*E*,3*E*)-5-((*E*)-1,3,3-trimethylindolin-2-ylidene)-penta-1,3-dien-1-yl)-3*H*-indol-1-ium 2,2,2-trifluoroacetate (2a)

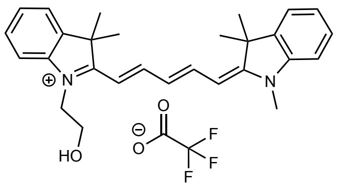

Yield: 54 mg, 68%

^{1}H NMR (400 MHz, CD_3OD): δ = 8.23 (td, *J* = 13.2 Hz, 5.4 Hz, 2H), 7.47 (d, *J* = 7.4 Hz, 2H), 7.40 (q, *J* = 7.6 Hz, 7.1 Hz, 2H), 7.33 – 7.22 (m, 4H), 6.59 (t, *J* = 12.4 Hz, 1H), 6.35 (d, *J* = 13.7 Hz, 1H), 6.25 (d, *J* = 13.7 Hz, 1H), 4.23 (t, *J* = 5.4 Hz, 2H), 3.94 (t, *J* = 5.4 Hz, 2H), 3.61 (s, 3H), 1.74 (s, 6H), 1.72 (s, 6H) ppm.

^{13}C{^{1}H} NMR (101 MHz, CD_3OD): δ = 175.7, 175.2, 155.5, 155.4, 155.3, 155.2, 144.3, 144.1, 142.6, 142.5, 129.7, 129.6, 126.2, 126.2, 126.2, 123.3, 123.3, 112.4, 111.8, 60.0, 50.6, 50.4, 47.7, 31.4, 28.0, 27.8 ppm.

HRMS (ESI) calcd for $[C_{28}H_{33}N_2O]^+$: 413.25874, found 413.25813.

1-(2-Hydroxyethyl)-3,3-dimethyl-2-((1*E*,3*E*)-5-((*E*)-1,3,3-trimethyl-5-(trifluoromethyl) indolin-2-ylidene)penta-1,3-dien-1-yl)-3*H*-indol-1-ium 2,2,2-trifluoroacetate (2b)

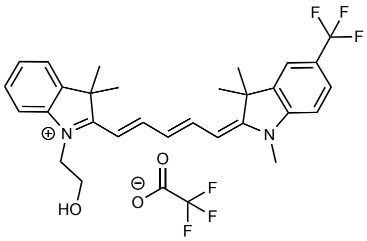

Yield: 71 mg, 79%

^{1}H NMR (400 MHz, CD_3OD): δ = 8.31 (t, *J* = 13.1 Hz, 1H), 8.19 (t, *J* = 13.1 Hz, 1H), 7.69 (s, 1H), 7.65 (d, *J* = 10.0 Hz, 1H), 7.54 (d, *J* = 7.4 Hz, 1H), 7.47 – 7.41 (m, 2H), 7.37 – 7.31 (m, 1H), 7.27 (d, *J* = 8.3 Hz, 1H), 6.63 (t, *J* = 12.4 Hz, 1H), 6.54 (d, *J* = 14.2 Hz, 1H), 6.14 (d, *J* = 13.3 Hz, 1H), 4.33 (t, *J* = 5.3 Hz, 2H), 3.98 (t, *J* = 5.3 Hz, 2H), 3.53 (s, 3H), 1.76 (s, 6H), 1.73 (s, 6H) ppm.

^{13}C{^{1}H} NMR (101 MHz, CD_3OD): δ = 178.8, 172.7, 157.2, 154.0, 147.7, 143.7, 143.3, 142.7, 129.8, 127.6, 127.5, 127.3 (q, *J* = 3.9 Hz), 126.7 (q, *J* = 32.6 Hz), 125.9 (q, *J* = 270.8 Hz), 123.5, 120.3 (q, *J* = 3.7 Hz), 113.5, 110.9, 107.6, 103.5, 60.2, 51.6, 51.5, 49.6, 48.3, 27.9, 27.7 ppm.

HRMS (ESI) calcd for $[C_{29}H_{32}F_3N_2O]^+$: 481.24612, found 481.24606.

2-((1*E*,3*E*)-5-((*E*)-3,3-Dimethyl-1-(pent-4-yn-1-yl)indolin-2-ylidene)penta-1,3-dien-1-yl)-3,3-dimethyl-1-(2-(methylamino)-2-oxoethyl)-3*H*-indol-1-ium 2,2,2-trifluoroacetate (14)

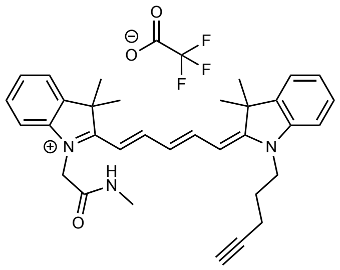

Compound **12** (100 mg, 0.28 mmol, 1.0 equiv.) and **8** (86 mg, 0.34 mmol, 1.2 equiv.) were dissolved in Ac_2O (2 mL) in a microwave vial. The solution was heated to 120 °C for 1 h in the microwave. The mixture was cooled to room temperature and a solution of **7c** (108 mg, 0.31 mmol, 1.1 equiv.) in pyridine (2 mL) was added dropwise. The mixture was stirred for 30 min at 18 °C. Solvents were removed under reduced pressure and the residue was purified via reverse phase column chromatography (10 → 70% CH_3CN in ddH_2O + 0.1% TFA over 35 min) to afford the product as a fine blue powder (87 mg, 52%).

^{1}H NMR (400 MHz, CD_3OD): δ = 8.32 – 8.22 (m, 2H), 7.52 (d, *J* = 7.5 Hz, 1H), 7.48 (d, *J* = 7.5 Hz, 1H), 7.46 – 7.41 (m, 1H), 7.40 – 7.34 (m, 2H), 7.33 – 7.29 (m, 1H), 7.24 (t, *J* = 7.9 Hz, 1H), 7.15 (d, *J* = 7.5 Hz, 1H), 6.57 (t, *J* = 12.4 Hz, 1H), 6.42 (d, *J* = 13.9 Hz, 1H), 6.13 (d, *J* = 13.6 Hz, 1H), 4.79 (s, 2H), 4.26 (t, *J* = 7.4 Hz, 2H), 2.82 (s, 3H), 2.50 (t, *J* = 2.6 Hz, 1H), 2.38 (td, *J* = 6.6, 2.6 Hz, 2H), 2.04 – 1.98 (m, 2H), 1.77 (s, 6H), 1.74 (s, 6H) ppm.

^{13}C NMR (101 MHz, CD_3OD) δ = 175.9, 175.3, 168.3, 156.4, 155.5, 144.1, 143.3, 142.8, 142.1, 129.8, 129.6, 127.9, 126.8, 126.0, 123.5, 123.3, 112.3, 111.3, 105.2, 104.1, 83.7, 71.4, 50.9, 50.5, 47.2, 44.0, 28.1, 27.8, 27.3, 26.6, 16.5 ppm.

HRMS (ESI) calcd for $[C_{33}H_{38}N_3O]^+$: 492.30149, found 492.30118.

Synthesis of 5-*exo-trig* carbocyanines

General procedure for the synthesis of 5-*exo-trig* Cy5 methyl esters (9)

In a flame-dried flask, compound **6** (50 mg, 0.16 mmol, 1.0 equiv.) was dissolved in anhydrous CH_3CN (5 mL) and oxalyl chloride (19 μL, 0.22 mmol, 1.3 equiv.) was added dropwise. The solution was stirred for 1 h at 18 °C. Anhydrous CH_3OH (5 mL) was added and the solution was stirred for additional 15 min. Solvents were removed under reduced pressure and the residue was used directly without further purification. The residue was dissolved in Ac_2O (1 mL) in a microwave vial. Compound **8** (51 mg, 0.20 mmol, 1.2 equiv.) was added and the solution was heated to 120 °C for 1 h in the microwave. The mixture was cooled to room temperature and a solution of **7** (0.18 mmol, 1.1 equiv.) in pyridine (1 mL) was added dropwise. The mixture was heated in the microwave at 110 °C for 30 min. Solvents were removed under reduced pressure and the residue was purified via reverse-phase column chromatography (10 → 55% CH_3CN in ddH_2O + 0.1% TFA over 35 min) to afford the product as a fine blue powder.

3-(2-Methoxy-2-oxoethyl)-1,3-dimethyl-2-((1*E*,3*E*)-5-((*E*)-1,3,3-trimethylindolin-2-ylidene)penta-1,3-dien-1-yl)-3*H*-indol-1-ium 2,2,2-trifluoroacetate (9a)

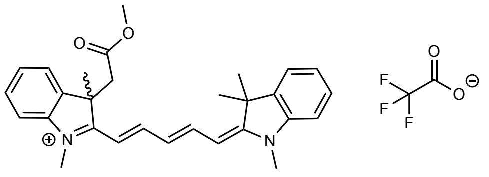

Yield: 57 mg, 61%

^{1}H NMR (400 MHz, CD_3OD): δ = 8.27 – 8.16 (m, 2H), 7.49 – 7.39 (m, 4H), 7.30 – 7.21 (m, 4H), 6.60 (t, *J* = 12.4 Hz, 1H), 6.27 (m, 2H), 3.63 (d, *J* = 17,4 Hz, 1H), 3.63 (s, 3H), 3,61 (s, 3H), 3,42 (d, *J* = 17.4 Hz, 1 H), 3.40 (s, 3H), 1.72 (s, 3H), 1.72 (s, 3H), 1.67 (s, 3H) ppm.

^{13}C{^{1}H} NMR (101 MHz, CD_3OD): δ = 175.3, 173.7, 171.4, 155.4, 154.7, 145.3, 144.2, 142.5, 140.1, 130.0, 129.7, 126.6, 126.2, 126.0, 123.3, 123.0, 111.8, 111.7, 104.4, 104.3, 52.1, 51.4, 50.4, 44.9, 31.5, 31.4, 27.8, 27.6 ppm.

HRMS (ESI) calcd for $[C_{29}H_{33}N_2O_2]^+$: 441.25420, found 441.25313.

3-(2-Methoxy-2-oxoethyl)-1,3-dimethyl-2-((1*E*,3*E*)-5-((*E*)-1,3,3-trimethyl-5-(trifluoro-methyl)indolin-2-ylidene)penta-1,3-dien-1-yl)-3*H*-indol-1-ium 2,2,2-trifluoroacetate (9b)

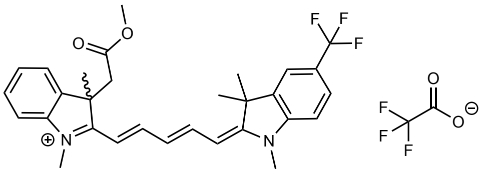

Yield: 67 mg, 64%

^{1}H NMR (400 MHz, CD_3OD): δ = 8.32 – 8.19 (m, 2H), 7.73 (s, 1H), 7.68 (d, *J* = 8.4 Hz, 1H), 7.55 (d, *J* = 7.5 Hz, 1H), 7.50 – 7.42 (m, 2H), 7.36 – 7.30 (m, 2H), 6.66 (t, *J* = 12.4 Hz, 1H), 6.50 (d, *J* = 14.3 Hz, 1H), 6.17 (d, *J* = 13.3 Hz, 1H), 3.76 (s, 3H), 3.70 (d, *J* = 17.5 Hz, 1H), 3.55 (s, 3H), 3.50 (d, *J* = 17.7 Hz, 1H), 3.41 (s, 3H), 1.75 (s, 3H), 1.74 (s, 3H), 1.70 (s, 3H) ppm.

^{13}C{^{1}H} NMR (101 MHz, CD_3OD): δ = 176.7, 172.8, 171.3, 156.4, 154.2, 147.7, 145.0, 142.7, 140.8, 130.2, 127.6, 127.35, 127.30 (q, *J* = 3.7 Hz), 126.6 (q, *J* = 32 Hz), 124.6, 120.3 (q, *J* = 3.7 Hz), 112.9, 110.9, 107.0, 103.5, 52.3, 52.2, 44.7, 32.1, 31.1, 27.92, 27.90, 27.2 ppm.

HRMS (ESI) calcd for $[C_{30}H_{32}F_3N_2O_2]^+$: 509.24159, found 509.24065.

2-((1*E*,3*E*)-5-((*E*)-3,3-Dimethyl-1-(pent-4-yn-1-yl)indolin-2-ylidene)penta-1,3-dien-1-yl)-3-(2-methoxy-2-oxoethyl)-1,3-dimethyl-3*H*-indol-1-ium 2,2,2-trifluoro-acetate (9c)

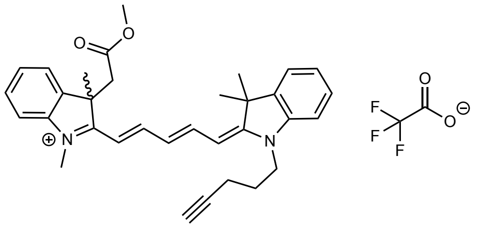

Yield: 67 mg, 66%

^{1}H NMR (400 MHz, CD_3OD): δ = 8.28 – 8.18 (m, 2H), 7.49 (d, *J* = 7.5 Hz, 2H), 7.45 – 7.38 (m, 2H), 7.31 (d, *J* = 7.9 Hz, 2H), 7.25 (t, *J* = 8.0 Hz, 2H), 6.59 (t, *J* = 12.4 Hz, 1H), 6.35 – 6.30 (m, 2H), 4.20 (t, *J* = 7.4 Hz, 2H), 3.65 (s, 3H), 3.64 (d, *J* = 17.3 Hz, 1H), 3.44 (d, *J* = 17.4 Hz, 1H), 3.40 (s, 3H), 2.50 (t, *J* = 2.6 Hz, 1H), 2.37 (td, *J* = 6.6, 2.6 Hz, 2H), 2.03 – 1.96 (m, 2H), 1.73 (s, 6H), 1.73 (s, 3H), 1.68 (s, 3H) ppm.

^{13}C{^{1}H} NMR (101 MHz, CD_3OD): δ = 174.4, 174.2, 171.4, 155.3, 155.0, 145.3, 143.6, 142.5, 140.2, 130.1, 129.7, 126.6, 126.3, 126.1, 123.4, 123.0, 111.9, 111.7, 104.9, 104.0, 83.8, 71.3, 52.1, 51.6, 50.4, 44.9, 43.6, 31.7, 28.0, 27.5, 27.2, 16.5 ppm.

HRMS (ESI) calcd for $[C_{33}H_{37}N_2O_2]^+$: 493.28550, found 493.28501.

2-((1*E*,3*E*)-5-((*E*)-3,3-Dimethyl-1-(pent-4-yn-1-yl)-5-(trifluoromethyl)indolin-2-ylidene) penta-1,3-dien-1-yl)-3-(2-methoxy-2-oxoethyl)-1,3-dimethyl-3*H*-indol-1-ium 2,2,2-tri-fluoroacetate (9d)

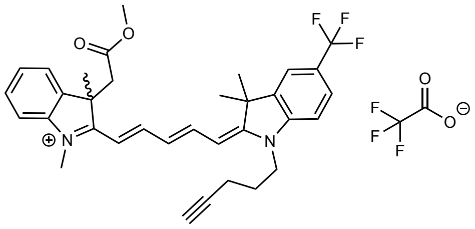

Yield: 71 mg, 63%
^{1}H NMR (400 MHz, CD_3OD): δ = 8.33 – 8.18 (m, 2H), 7.73 (s, 1H), 7.67 (d, *J* = 8.3 Hz, 1H), 7.56 (d, *J* = 7.1 Hz, 1H), 7.51 – 7.44 (m, 2H), 7.35 (t, *J* = 7.4 Hz, 2H), 6.64 (t, *J* = 12.4 Hz, 1H), 6.53 (d, *J* = 14.4 Hz, 1H), 6.26 (d, *J* = 13.3 Hz, 1H), 4.15 (t, *J* = 7.4 Hz, 2H), 3.77 (s, 3H), 3.71 (d, *J* = 17.5 Hz, 1H), 3.51 (d, *J* = 17.5 Hz, 1H), 3.41 (s, 3H), 2.49 (t, *J* = 2.6 Hz, 1H), 2.37 (td, *J* = 6.7, 2.6 Hz, 2H), 1.98 (p, *J* = 7.3 Hz, 2H), 1.76 (s, 3H), 1.75 (s, 3H), 1.70 (s, 3H) ppm.
^{13}C{^{1}H} NMR (101 MHz, CD_3OD): δ = 177.1, 171.7, 171.3, 156.4, 153.9, 147.1, 144.9, 142.7, 140.9, 130.3, 127.7, 127.5, 127.3 (q, *J* = 3.7 Hz), 126.6 (q, *J* = 32 Hz), 125.9 (q, *J* = 270.8 Hz), 123.2, 120.43 (q, *J* = 3.7 Hz), 113.0, 110.9, 107.4, 103.3, 83.8, 71.3, 52.4, 52.2, 44.7, 43.4, 32.3, 28.0, 27.1, 26.9, 16.5 ppm.
HRMS (ESI) calcd for $[C_{34}H_{36}F_3N_2O_2]^+$: 561.27289, found 561.27194.

General procedure for the synthesis of 5-*exo-trig* Cy5 alcohols (4)

Methyl ester **9** (40 mg, 1.0 equiv.) was dissolved in anhydrous THF (3 mL) in a flame-dried flask and cooled to 0 °C. $LiAlH_4$ (4.0 equiv.) was added, and the solution was stirred for 1 h at 0 °C, followed by 16 h at 18 °C. The mixture was cooled again to 0 °C and water was added. The aqueous phase was extracted trice with CH_2Cl_2, and the combined organic phases were washed with brine and dried over Na_2SO_4. DDQ (1.0 equiv.) was added to re-oxidize the conjugated system of the carbocyanine core. Solvents were removed under reduced pressure and the residue was purified via reverse-phase column chromatography (10 $\rightarrow$ 65% CH_3CN in ddH_2O + 0.1% TFA over 35 min) to afford the product as a fine blue powder.

3-(2-Hydroxyethyl)-1,3-dimethyl-2-((1*E*,3*E*)-5-((*E*)-1,3,3-trimethylindolin-2-ylidene)-penta-1,3-dien-1-yl)-3*H*-indol-1-ium 2,2,2-trifluoroacetate (4a)

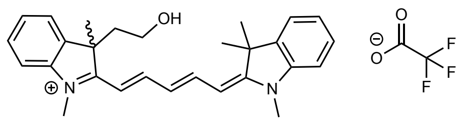

Yield: 21 mg, 55%

^{1}H NMR (400 MHz, CD_3OD): δ = 8.24 (t, *J* = 13.1 Hz, 2H), 7.50 – 7.40 (m, 4H), 7.30 – 7.24 (m, 4H), 6.62 (t, *J* = 12.4 Hz, 1H), 6.30 (d, *J* = 4.9 Hz, 1H), 6.26 (d, *J* = 4.9 Hz, 1H), 3.62 (s, 3H), 3.60 (s, 3H), 3.14 – 3.06 (m, 1H), 3.04 – 2.97 (m, 1H), 2.75 – 2.63 (m, 1H), 2.56 – 2.35 (m, 1H), 1.73 (s, 9H) ppm.

^{13}C{^{1}H} NMR (101 MHz, CD_3OD): δ = 175.4, 173.8, 155.4, 155.0, 145.0, 144.3, 142.5, 140.2, 129.9, 129.7, 126.6, 126.3, 126.2, 123.4, 123.3, 111.82, 111.80, 104.8, 104.4, 59.2, 57.5, 53.0, 50.5, 44.5, 31.5, 28.2, 27.8 ppm.

HRMS (ESI) calcd for $[C_{28}H_{33}N_2O]^+$: 413.25874, found 413.25819.

3-(2-Hydroxyethyl)-1,3-dimethyl-2-((1*E*,3*E*)-5-((*E*)-1,3,3-trimethyl-5-(trifluoromethyl)-indolin-2-ylidene)penta-1,3-dien-1-yl)-3*H*-indol-1-ium 2,2,2-trifluoroacetate (4b)

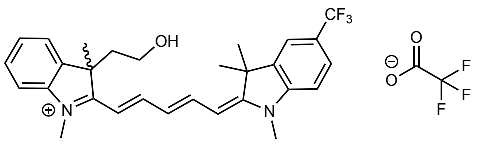

Yield: 24 mg, 63%

^{1}H NMR (400 MHz, CD_3OD): δ = 8.33 (t, *J* = 13.1 Hz, 1H), 8.21 (t, *J* = 13.1 Hz, 1H), 7.73 (s, 1H), 7.67 (d, *J* = 8.4 Hz, 1H), 7.55 (d, *J* = 7.6 Hz, 1H), 7.50 (t, *J* = 7.0 Hz, 1H), 7.45 – 7.36 (m, 2H), 7.30 (d, *J* = 8.4 Hz, 1H), 6.67 (t, *J* = 12.5 Hz, 1H), 6.50 (d, *J* = 14.2 Hz, 1H), 6.17 (d, *J* = 13.3 Hz, 1H), 3.72 (s, 3H), 3.55 (s, 3H), 3.12 – 3.01 (m, 2H), 2.80 – 2.72 (m, 1H), 2.51 (m, 1H), 1.75 (s, 3H), 1.74 (s, 6H) ppm.

$^{13}C\{^{1}H\}$ NMR (101 MHz, CD_3OD): δ = 177.3, 172.6, 156.7, 153.9, 147.7, 144.7, 142.6, 140.9, 130.1, 127.6, 127.5, 127.3 (q, *J* = 3.7 Hz), 126.6 (q, *J* = 32.7 Hz), 124.6, 123.7, 120.3 (q, *J* = 3.7 Hz), 113.0, 110.9, 107.6, 103.4, 59.1, 57.5, 54.0, 44.5, 32.2, 31.1, 27.9 ppm.

HRMS (ESI) calcd for $[C_{29}H_{32}F_3N_2O]^+$: 481.24612, found 481.24588.

2-((1*E*,3*E*)-5-((*E*)-3,3-Dimethyl-1-(pent-4-yn-1-yl)-5-(trifluoromethyl)indolin-2-ylidene)-penta-1,3-dien-1-yl)-3-(2-hydroxyethyl)-1,3-dimethyl-3*H*-indol-1-ium 2,2,2-trifluoro-acetate (4c)

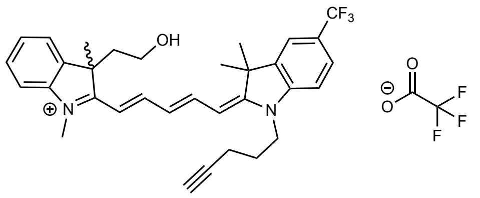

Yield: 26 mg, 69%

^{1}H NMR (400 MHz, CD_3OD): δ = 8.34 (t, *J* = 13.2 Hz, 1H), 8.20 (t, *J* = 13.0 Hz, 1H), 7.73 (s, 1H), 7.67 (d, *J* = 8.4 Hz, 1H), 7.56 (d, *J* = 6.7 Hz, 1H), 7.53 – 7.48 (m, 1H), 7.46 (d, *J* = 8.1 Hz, 1H), 7.40 (td, *J* = 7.3, 1.3 Hz, 1H), 7.33 (d, *J* = 8.4 Hz, 1H), 6.66 (t, *J* = 12.4 Hz, 1H), 6.53 (d, *J* = 14.3 Hz, 1H), 6.26 (d, *J* = 13.1 Hz, 1H), 4.15 (t, *J* = 7.4 Hz, 2H), 3.74 (s, 3H), 3.15 – 3.00 (m, 2H), 2.82 – 2.72 (m, 1H), 2.57 – 2.50 (m, 1H), 2.50 (t, *J* = 2.6 Hz, 1H), 2.37 (td, *J* = 6.6, 2.6 Hz, 2H), 1.98 (p, *J* = 6.8 Hz, 2H), 1.75 (s, 9H) ppm.

$^{13}C\{^{1}H\}$ NMR (101 MHz, CD_3OD): δ = 177.7, 171.5, 156.8, 153.5, 147.2, 144.6, 142.6, 141.0, 130.1, 127.74, 127.65, 127.3 (q, *J* = 3.7 Hz), 126.6 (q, *J* = 32.6 Hz), 124.6,

123.7, 120.4 (q, J = 3.7 Hz), 113.2, 110.9, 108.0, 103.2, 83.9, 71.3, 59.2, 54.2, 44.5, 43.4, 32.3, 28.1, 27.9, 26.9, 16.5 ppm.
HRMS (ESI) calcd for $[C_{33}H_{36}F_3N_2O]^+$: 533.27742, found 533.27686.

2-((1*E*,3*E*)-5-((*E*)-3,3-Dimethyl-1-(pent-4-yn-1-yl)indolin-2-ylidene)penta-1,3-dien-1-yl) 1,3-dimethyl-3-(2-(methylamino)-2-oxoethyl)-3*H*-indol-1-ium 2,2,2-trifluoroacetate (12)

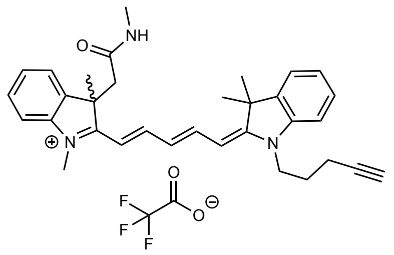

Compound **9c** (25 mg, 0.04 mmol, 1.0 equiv.) was dissolved in anhydrous THF (3 mL) and methyl amine (2.0 M solution in THF, 5 mL) was added. The solution was stirred at 70 °C for 16 h. Solvents were removed under reduced pressure and the residue was purified via reverse-phase column chromatography (10 → 55% CH_3CN in ddH_2O + 0.1% TFA over 35 min) to afford the product as a fine blue powder (24 mg, 96%).

^{1}H NMR (400 MHz, CD_3OD): δ = 8.18 (t, J = 13.3 Hz, 2H), 7.48 – 7.33 (m, 4H), 7.37 – 7.21 (m, 4H), 6.58 (t, J = 12.4 Hz, 1H), 6.36 (d, J = 14.0 Hz, 1H), 6.28 (d, J = 13.5 Hz, 1H), 4.17 (t, J = 7.5 Hz, 2H), 3.67 (s, 3H), 3.40 (d, J = 15.6 Hz, 1H), 3.21 (d, J = 15.6 Hz, 1H), 2.50 (t, J = 2.6 Hz, 1H), 2.43 (s, 3H), 2.36 (td, J = 6.6, 2.6 Hz, 2H), 1.99 (p, J = 6.8 Hz, 2H), 1.72 (s, 3H), 1.72 (s, 3H) 1.68 (s, 3H) ppm.
$^{13}C\{^1H\}$ NMR (101 MHz, CD_3OD): δ = 175.7, 173.4, 171.4, 155.3, 154.5, 145.1, 143.7, 142.3, 140.40 130.0, 129.7, 126.5, 126.4, 125.8, 123.4, 123.2, 112.3, 111.4, 105.7, 103.4, 83.8, 71.3, 52.4, 50.2, 46.7, 43.5, 31.8, 28.1, 27.4, 27.2, 26.0, 16.5 ppm.
HRMS (ESI) calcd for $[C_{33}H_{38}N_3O]^+$: 492.30149, found 492.30128.

(4*R*,7*R*,10*S*,13*S*,19*S*,*E*)-7-((1*H*-indol-3-yl)methyl)-10-(4-(4-(3-((*E*)-3,3-dimethyl-2-((2*E*,4*E*)-5-(1,3a,8-trimethyl-2-oxo-2,3,3a,8-tetrahydropyrrolo[2,3-*b*]indol-8a(1*H*)-yl)penta-2,4-dien-1-ylidene)indolin-1-yl)propyl)-1*H*-1,2,3-triazol-1-yl)butyl)-4-(4-hydroxyphenyl)-8,13,15,19-tetramethyl-1-oxa-5,8,11-triazacyclononadec-15-ene-2,6,9,12-tetraone (17-actin)

Boc-Lys-jasplakinolide (7 mg, 0.009 mmol, 1.0 equiv.) was dissolved in TFA:CH_2Cl_2 (3:7 v/v, 0.3 mL) and stirred at 18 °C for 7.5 min. Toluene (0.6 mL) was added and solvents were removed under reduced pressure. Co-evaporation was carried out twice with 0.4 mL THF, and once with 0.4 mL THF and 10 μL triethylamine. The residue was used directly for the diazotizing reaction. The residue was dissolved in DMF/MTBE/H_2O (94:5:1, 0.3 mL) and FSO_2N_3 solution (1.35 equiv., 25 μL of a 488 mM solution) and aqueous potassium hydrogen carbonate solution (3 M, 0.3 mL) were added. The mixture was stirred for 1 h at 18 °C. Ethyl acetate was added and the organic phase was washed trice with brine, once with water and again with brine. The extracted organic phase was dried over Na_2SO_4, filtered, and solvents were removed under reduced pressure to yield azide-modified Lys-jasplakinolide, which was used directly in the Click reaction without further purification.
In a flame-dried flask, **12** (2.7 mg, 0.0045 mmol, 0.5 equiv.) and azide-modified Lys-jasplakinolide were dissolved in degassed THF (2 mL) and the solution was degassed for an additional hour by bubbling argon through the solution while stirring vigorously. $Cu(PPh_3)_2NO_3$ (1.5 mg, 0.0023 mmol, 0.25 equiv.) and triethylamine (5 μL) were added and the solution was stirred for 16 h at room temperature. Solvents were removed under reduced pressure and the residue was purified via reverse-phase column chromatography (10 → 90% CH_3CN in ddH_2O over 35 min).

Yield: 4.5 mg (42 %)
HRMS (ESI) calcd for $[C_{71}H_{87}N_{10}O_7]^+$: 1191.67537, found 1191.67359.

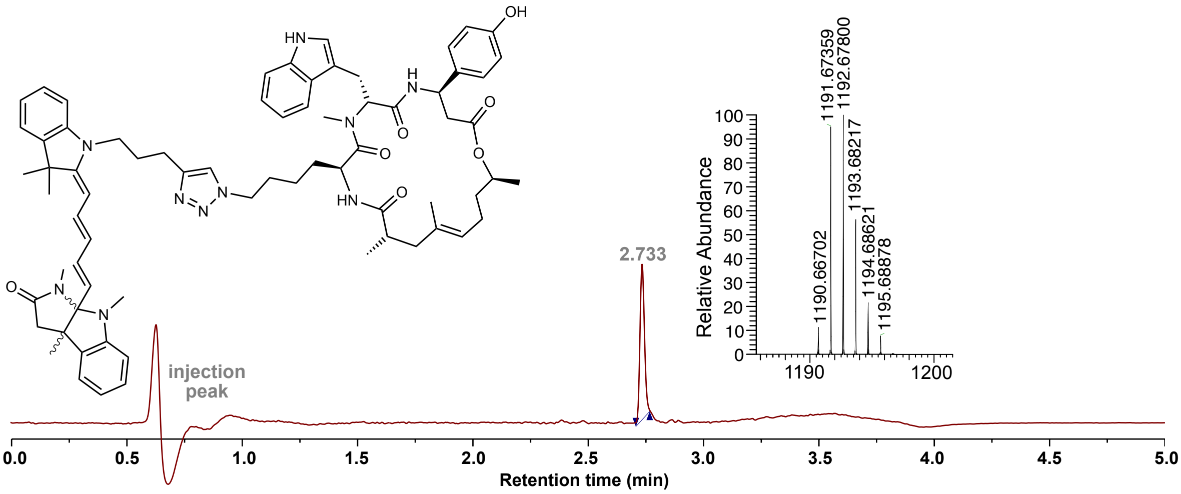

8a-((1*E*,3*E*)-5-((*E*)-3,3-dimethyl-1-(3-(1-(4-(4-(6-(4-methylpiperazin-1-yl)-1*H*,3'*H*-[2,5'-bibenzo[*d*]imidazol]-2'-yl)phenoxy)butyl)-1*H*-1,2,3-triazol-4-yl)propyl)-indolin-2-ylidene)penta-1,3-dien-1-yl)-1,3a,8-trimethyl-3,3a,8,8a-tetrahydro-pyrrolo[2,3-*b*]indol-2(1*H*)-one (18-DNA)

Boc-C4-Hoechst (6.3 mg, 0.011 mmol, 1.0 equiv.) was dissolved in dry CH_2Cl_2 (0.8 mL) and TFA (0.2 mL) was added dropwise. The mixture was stirred at 18 °C for 30 min. Solvents were removed under reduced pressure and co-evaporation with THF (2x 0.4 mL) was carried out twice. The residue was used directly for the diazotizing reaction. The residue was dissolved in DMF/MTBE/H_2O (94:5:1, 0.3 mL) and FSO_2N_3 solution (1.35 equiv., 30 μL of a 488 mM solution) and aqueous potassium hydrogen carbonate solution (3 M, 0.3 mL) were added. The mixture was stirred for 1 h at 18 °C. Ethyl acetate was added and the organic phase was washed trice with brine, once with water and again with brine. The extracted organic phase was dried over Na_2SO_4, filtered, and solvents were removed under reduced pressure to yield azide-modified C4-Hoechst, which was used directly in the Click reaction without further purification. In a flame-dried flask, **12** (3.2 mg, 0.0053 mmol, 0.5 equiv.) and azide-modified C4-Hoechst were dissolved in degassed THF (2 mL) and the solution was degassed for an additional hour by bubbling argon through the solution while stirring vigorously. $Cu(PPh_3)_2NO_3$ (1.7 mg, 0.0023 mmol, 0.25 equiv.) and triethylamine (5 μL) were added and the solution was stirred for 16 h at room temperature. Solvents were removed under reduced pressure and the residue was purified via reverse-phase column chromatography (10 → 55% CH_3CN in ddH_2O + 0.1% TFA over 35 min).

Yield: 2.9 mg (27 %)
HRMS (ESI) calcd for $[C_{62}H_{69}N_{12}O_2]^+$: 1013.56610, found 1013.56422.

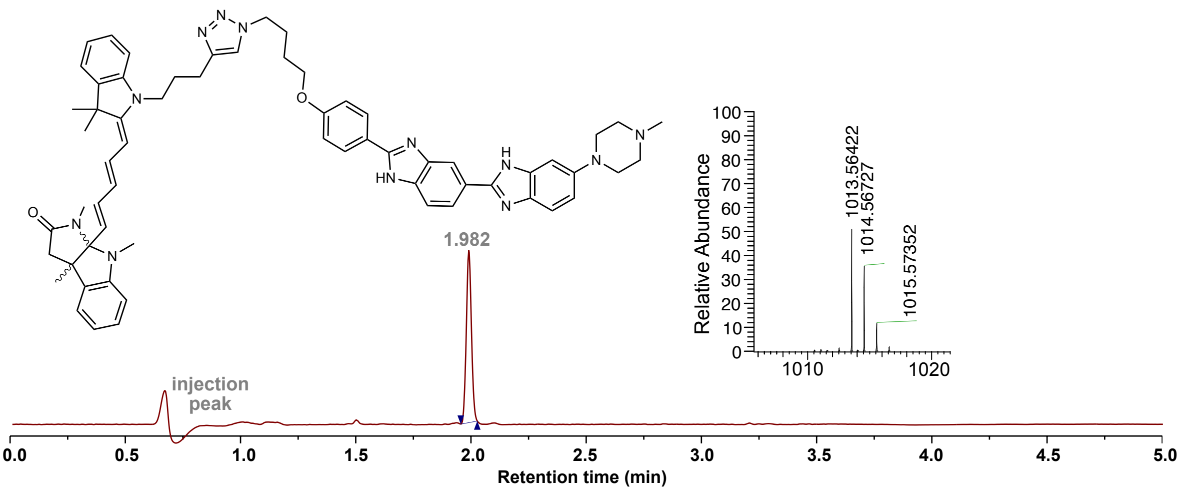

2-((*E*)-3-((*E*)-3,3-Dimethyl-1-(pent-4-yn-1-yl)-5-(trifluoromethyl)indolin-2-ylidene)prop-1-en-1-yl)-3-(2-methoxy-2-oxoethyl)-1,3-dimethyl-3*H*-indol-1-ium 2,2,2-trifluoro-acetate (21)

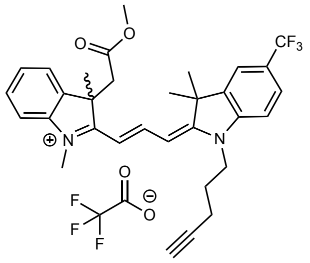

In a flame-dried flask, compound **6** (50 mg, 0.16 mmol, 1.0 equiv.) was dissolved in anhydrous CH_3CN (5 mL) and oxalyl chloride (19 μL, 0.22 mmol, 1.3 equiv.) was added dropwise. The solution was stirred for 1 h at 18 °C. Anhydrous CH_3OH (5 mL) was added and the solution was stirred for additional 15 min. Solvents were removed under reduced pressure and the residue was used directly without further purification. The residue was dissolved in Ac_2O (1 mL) in a microwave vial. *N,N'*-Diphenylformamidine (40 mg, 0.21 mmol, 1.2 equiv.) was added and the solution was heated to 95 °C for 1 h in the microwave. The mixture was cooled to room temperature and a solution of **7d** (74 mg, 0.18 mmol, 1.1 equiv.) in pyridine (1 mL) was added dropwise. The mixture was heated in the microwave at 100 °C for 30 min. Solvents were removed under reduced pressure and the residue was purified via reverse-phase column chromatography (10 → 60% CH_3CN in ddH_2O + 0.1% TFA over 35 min) to afford the product as a fine red powder (72 mg, 67%).

^{1}H NMR (400 MHz, CD_3OD): δ = 8.45 (t, *J* = 13.5 Hz, 1H), 7.85 (s, 1H), 7.74 (d, *J* = 9.4 Hz, 1H), 7.59 (d, *J* = 7.4 Hz, 1H), 7.53 – 7.45 (m, 3H), 7.39 – 7.35 (m, 1H), 6.56 (d, *J* = 13.9 Hz, 1H), 6.48 (d, *J* = 13.1 Hz, 1H), 4.24 (t, *J* = 7.4 Hz, 2H), 3.79 (s, 3H), 3.65 (d, *J* = 16.9 Hz, 1H), 3.42 (s, 3H), 3.36 (d, *J* = 17.1 Hz, 1H), 2.50 (t, *J* = 2.6 Hz, 1H), 2.40 (td, *J* = 6.8, 2.7 Hz, 2H), 2.04 (p, *J* = 6.8 Hz, 2H), 1.81 (s, 3H), 1.80 (s, 3H), 1.73 (s, 3H) ppm.

^{13}C{^{1}H} NMR (101 MHz, CD_3OD): δ = 175.5, 173.8, 169.4, 150.2, 145.3, 143.4, 141.2, 139.0, 129.0, 126.4 (q, *J* = 32 Hz), 126.2, 124.4 (q, *J* = 270 Hz), 121.9, 119.4 (q, *J* = 3.7 Hz), 111.7, 110.6, 104.5, 102.2, 82.3, 70.0, 51.0, 50.9, 48.8, 44.0, 42.6, 31.1, 26.9, 26.9, 26.2, 25.7, 15.2 ppm.

HRMS (ESI) calcd for $[C_{32}H_{34}F_3N_2O_2]^+$: 535.25724, found 535.25695.

2-((*E*)-3-((*E*)-3,3-Dimethyl-1-(pent-4-yn-1-yl)-5-(trifluoromethyl)indolin-2-ylidene)prop-1-en-1-yl)-1,3-dimethyl-3-(2-(methylamino)-2-oxoethyl)-3*H*-indol-1-ium 2,2,2-trifluoro-acetate (22)

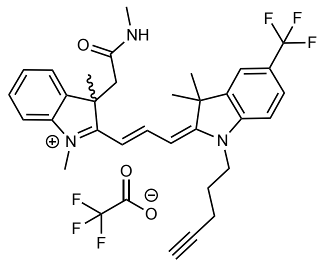

Compound **21** (25 mg, 0.04 mmol, 1.0 equiv.) was dissolved in anhydrous THF (3 mL) and methyl amine (2.0 M solution in THF, 5 mL) was added. The solution was stirred at 70 °C for 16 h. Solvents were removed under reduced pressure and the residue was purified via reverse-phase column chromatography (10 → 50% CH_3CN in ddH_2O + 0.1% TFA over 35 min) to afford the product as a fine red powder (24 mg, 96%).

^{1}H NMR (400 MHz, CD_3OD): δ = 8.45 (t, *J* = 13.5 Hz, 1H), 7.82 (s, 1H), 7.72 (d, *J* = 8.4 Hz, 1H), 7.53 (d, *J* = 7.5 Hz, 1H), 7.49 (dd, *J* = 7.1, 1.2 Hz, 1H), 7.47 – 7.43 (m, 2H), 7.37 (td, *J* = 7.3, 1.3 Hz, 1H), 6.57 (d, *J* = 13.9 Hz, 1H), 6.43 (d, *J* = 12.9 Hz, 1H), 4.22 (t, *J* = 7.4 Hz, 2H), 3.80 (s, 3H), 3.39 (d, *J* = 15.9 Hz, 1H), 3.23 (d, *J* = 15.9 Hz, 1H), 2.50 (t, *J* = 2.7 Hz, 1H), 2.43 (s, 3H), 2.39 (td, *J* = 6.8, 2.6 Hz, 2H), 2.03 (p, *J* = 6.9 Hz, 2H), 1.79 (s, 3H), 1.78 (s, 3H), 1.72 (s, 3H) ppm.
^{13}C{^{1}H} NMR (101 MHz, CD_3OD): δ = 178.4, 174.2, 170.5, 151.4, 146.8, 144.8, 142.4, 140.4, 130.3, 127.6 (q, *J* = 4.1 Hz), 127.53, 127.47 (q, *J* = 32 Hz), 125.8 (q, *J* = 270.7 Hz), 123.3, 120.7 (q, *J* = 3.5 Hz), 113.3, 111.7, 106.7, 103.1, 83.7, 71.4, 52.9, 50.0, 47.1, 43.9, 32.6, 28.5, 28.4, 27.4, 27.0, 26.0, 16.6 ppm.
HRMS (ESI) calcd for $[C_{32}H_{35}F_3N_3O]^+$: 534.27322, found 534.27268.

3-(Carboxymethyl)-2-((1*E*,3*E*,5*E*)-7-((*E*)-3,3-dimethyl-1-(pent-4-yn-1-yl)indolin-2-ylidene)hepta-1,3,5-trien-1-yl)-1,3-dimethyl-3*H*-indol-1-ium 2,2,2-trifluoro-acetate (23)

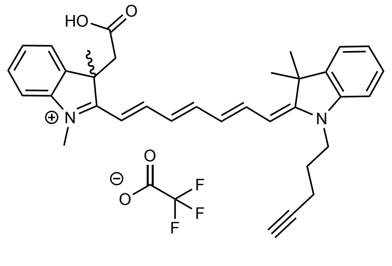

Compound **6** (100 mg, 0.34 mmol, 1.0 equiv.) and glutaconaldehydedianil hydrochloride (114 mg, 0.40 mmol, 1.2 equiv.) were dissolved in Ac_2O (2 mL) in a microwave vial. The solution was heated to 120 °C for 1 h in the microwave. The mixture was cooled to room temperature and a solution of **7c** (130 mg, 0.37 mmol, 1.1 equiv.) in pyridine (2 mL) was added dropwise. The mixture was heated to 100 °C for 30 min. Solvents were removed under reduced pressure and the residue was purified via reverse phase column chromatography (10 → 65% CH_3CN in ddH_2O + 0.1% TFA over 35 min) to afford the product as a fine green powder (95 mg, 46%).
^{1}H NMR (400 MHz, CD_3OD): δ = 7.97 (t, *J* = 12.7 Hz, 1H), 7.71 (t, *J* = 12.9 Hz, 1H), 7.51 (d, *J* = 7.5 Hz, 1H), 7.43 (td, *J* = 8.2, 1.3 Hz, 1H), 7.39 – 7.35 (m, 2H), 7.33 – 7.28

(m, 2H), 7.10 (d, *J* = 7.8 Hz, 2H), 6.58 – 6.39 (m, 4H), 6.04 (d, *J* = 13.3 Hz, 1H), 4.03 (t, *J* = 7.4 Hz, 2H), 3.72 (s, 3H), 3.38 (d, *J* = 16.8 Hz, 1H), 3.28 (d, *J* = 16.8 Hz, 1H), 2.47 (t, *J* = 2.7 Hz, 1H), 2.34 (dt, *J* = 6.9, 3.4 Hz, 2H), 1.94 (p, *J* = 6.8 Hz, 2H), 1.65 (s, 6H), 1.58 (s, 3H) ppm.

^{13}C NMR (101 MHz, CD_3OD) δ = 177.9, 175.6, 168.4, 156.1, 154.4, 148.5, 145.1, 144.4, 142.7, 141.5, 129.6, 129.4, 127.3, 126.2, 124.2, 123.2, 123.0, 112.9, 110.1, 108.7, 101.6, 84.0, 73.7, 71.6, 71.1, 53.5, 42.9, 32.2, 28.4, 27.4, 26.9, 16.6 ppm.

HRMS (ESI) calcd for $[C_{34}H_{37}N_2O_2]^+$: 505.28495, found 505.28459.

3-(2-((2,2-Difluoroethyl)amino)-2-oxoethyl)-2-((1*E*,3*E*,5*E*)-7-((*E*)-3,3-dimethyl-1-(pent-4-yn-1-yl)indolin-2-ylidene)hepta-1,3,5-trien-1-yl)-1,3-dimethyl-3*H*-indol-1-ium 2,2,2-trifluoro-acetate (24)

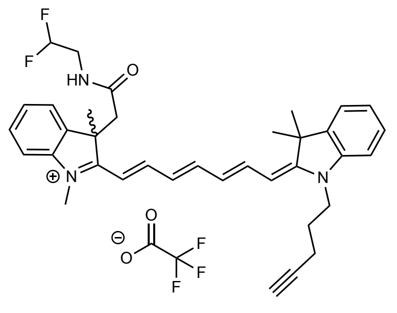

In a flame-dried flask, compound **23** (20 mg, 0.03 mmol, 1.0 equiv.) was dissolved in dry CH_2Cl_2 (5 mL) and 4-dimethylaminopyridine (15.8 mg, 0.13 mmol, 4.0 equiv.) and *N*-(3-dimethylamino-propyl)-3-ethyl carbodiimide hydrochloride (EDC-HCl, 24.8 mg, 0.13 mmol, 4.0 equiv.) were added. The solution was stirred at 18 °C for 30 min. 2,2-Difluoroethylamine hydrochloride (76 mg, 0.65 mmol, 20.0 equiv.) was added and the solution was stirred overnight at 18 °C. Solvents were removed under reduced pressure and the residue was purified via reverse phase column chromatography (10 → 75% CH_3CN in ddH_2O + 0.1% TFA over 35 min) to afford the product as a fine green powder (18 mg, 82%).

^{1}H NMR (400 MHz, CD_3CN): δ = 7.89 – 7.83 (m, 1H), 7.78 – 7.72 (m, 1H), 7.58 (t, *J* = 13.0 Hz, 1H), 7.47 (d, *J* = 7.9 Hz, 1H), 7.43 – 7.39 (m, 2H), 7.37 – 7.33 (m, 1H), 7.26 (t, *J* = 7.6 Hz, 2H), 7.17 (d, *J* = 7.8 Hz, 2H), 6.53 – 6.42 (m, 2H), 6.30 (d, *J* = 14.2 Hz, 1H), 6.15 (d, *J* = 13.4 Hz, 1H), 5.55 (tt, *J* = 56.0, 3.9 Hz, 1H), 4.02 (t, *J* = 7.5 Hz, 2H), 3.57 (s, 3H), 3.37 (d, *J* = 16.0 Hz, 1H), 3.32 – 3.22 (m, 3H), 2.36 – 2.31 (m, 3H), 1.64 (s, 6H), 1.60 (s, 3H) ppm.

^{13}C NMR (101 MHz, CD_3CN) δ = 174.5, 171.0, 169.9, 156.6, 152.9, 150.6, 144.8, 143.8, 141.8, 140.6, 129.7, 129.4, 126.6, 126.2, 125.0, 123.2, 117.6, 115.2, 112.8, 112.1, 111.0, 106.3, 103.1, 84.2, 71.1, 68.3, 52.0, 49.5, 46.4, 43.4, 41.8 (t, *J* = 26.7 Hz), 32.3, 28.0, 27.4, 26.7, 16.4 ppm.

HRMS (ESI) calcd for $[C_{36}H_{40}F_2N_3O]^+$: 568.31340, found 568.31309.

Click reactions

General procedure for the click reaction of carbocyanines bearing an alkyne handle (4c, 13, 14, 22, 24) to azide-modified SNAP-tag ligand 10

In a flame-dried flask, alkyne-modified carbocyanine dye (15 mg, 1.0 equiv.) and **10** (1.5 equiv.) were dissolved in degassed THF (5 mL) and the solution was degassed for an additional hour by bubbling argon through the solution while stirring vigorously. $Cu(PPh_3)_2NO_3$ (0.5 equiv.) and triethylamine (10 µL) were added and the solution was stirred for 16 h at room temperature. Solvents were removed under reduced pressure and the residue was purified via reverse-phase column chromatography (10 → 45% CH_3CN in ddH_2O + 0.1% TFA over 35 min).

2-((1*E*,3*E*)-5-((*E*)-1-(3-(1-(4-(((2-Amino-9*H*-purin-6-yl)oxy)methyl)benzyl)-1*H*-1,2,3-triazol-4-yl)propyl)-3,3-dimethyl-5-(trifluoromethyl)indolin-2-ylidene)penta-1,3-dien-1-yl)-3-(2-hydroxyethyl)-1,3-dimethyl-3*H*-indol-1-ium 2,2,2-trifluoroacetate (11)

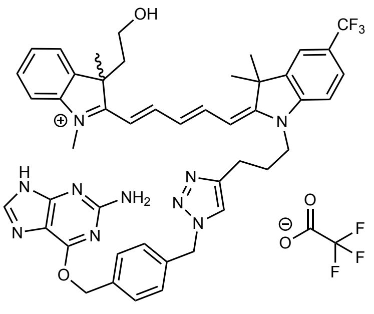

Yield: 10 mg, 45%

^{1}H NMR (400 MHz, CD_3OD): δ = 8.36 – 8.29 (m, 1H), 8.26 – 8.151 (m, 2H), 7.83 (s, 1H), 7.71 (s, 1H), 7.60 – 7.54 (m, 4H), 7.52 – 7.48 (m, 1H), 7.44 – 7.37 (m, 4H), 7.19 (d, *J* = 8.4 Hz, 1H), 6.60 (t, *J* = 12.4 Hz, 1H), 6.49 (d, *J* = 14.2 Hz, 1H), 6.13 (d, *J* = 13.1 Hz, 1H), 5.62 (s, 2H), 5.61 (s, 2H), 4.07 (t, *J* = 7.6 Hz, 2H), 3.73 (s, 3H), 3.13 – 3.00 (m, 2H), 2.86 (t, *J* = 7.3 Hz, 2H), 2.79 – 2.72 (m, 1H), 2.55 – 2.47 (m, 1H), 2.21 – 2.13 (m, 2H), 1.74 (s, 3H), 1.72 (s, 6H) ppm.

^{13}C{^{1}H} NMR (126 MHz, CD_3OD): δ = 177.6, 171.4, 161.1, 158.9, 156.7, 153.6, 148.1, 147.1, 144.6, 142.7, 141.0, 137.4, 137.2, 130.4, 130.1, 129.4, 129.2, 127.8, 127.6, 127.3 (q, *J* = 4.2 Hz), 125.9 (q, *J* =270.8 Hz), 123.8, 123.7, 120.4 (q, *J* = 3.3 Hz), 113.1, 110.9, 107.9, 103.4, 70.1, 59.2, 54.5, 54.1, 44.5, 43.9, 36.5, 32.3, 28.0, 27.9, 27.3, 23.4, 14.4 ppm.

HRMS (ESI) calcd for $[C_{46}H_{48}F_3N_{10}O_2]^+$: 829.39083, found 829.38980.

2-((1*E*,3*E*)-5-((*E*)-1-(3-(1-(4-(((2-Amino-9*H*-purin-6-yl)oxy)methyl)benzyl)-1*H*-1,2,3-triazol-4-yl)propyl)-3,3-dimethylindolin-2-ylidene)penta-1,3-dien-1-yl)-1,3-dimethyl-3-(2-(methylamino)-2-oxoethyl)-3*H*-indol-1-ium 2,2,2-trifluoroacetate (15)

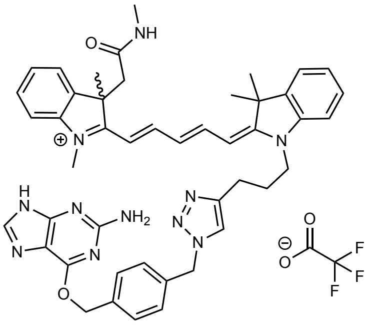

Yield: 14 mg, 64%

^{1}H NMR (400 MHz, CD_3OD): δ = 8.30 (s, 1H), 8.16 (td, *J* = 13.0, 12.6, 5.9 Hz, 2H), 7.83 (s, 1H), 7.57 (d, *J* = 8.3 Hz, 2H), 7.45 – 7.37 (m, 5H), 7.31 (dd, *J* = 8.4, 3.2 Hz, 2H), 7.26 (t, *J* = 8.0 Hz, 1H), 7.19 (t, *J* = 7.5 Hz, 1H), 7.14 (d, *J* = 7.8 Hz, 1H), 6.52 (t, *J* = 12.4 Hz, 1H), 6.32 (d, *J* = 14.0 Hz, 1H), 6.13 (d, *J* = 13.5 Hz, 1H), 5.63 (s, 2H), 5.61 (s, 2H), 4.10 (t, *J* = 7.6 Hz, 2H), 3.66 (s, 3H), 3.40 (d, *J* = 15.5 Hz, 1H), 3.20 (d, *J* = 15.8 Hz, 1H), 2.86 (t, *J* = 7.3 Hz, 2H), 2.43 (s, 3H), 2.18 (p, *J* = 7.3 Hz, 2H), 1.69 (s, 6H), 1.67 (s, 3H) ppm.

^{13}C{^{1}H} NMR (151 MHz, CD_3OD): δ = 175.6, 173.4, 171.4, 161.1, 158.6, 155.2, 154.5, 148.1, 145.1, 143.7, 142.3, 140.4, 137.5, 130.5, 130.0, 129.6, 129.4, 126.5, 126.3, 125.7, 123.8, 123.4, 123.2, 112.2, 111.5, 105.5, 103.5, 70.3, 54.5, 52.3, 50.19, 46.73, 44.0, 31.8, 28.04, 28.02, 27.6, 27.5, 26.0, 23.4 ppm.

HRMS (ESI) calcd for $[C_{46}H_{50}N_{11}O_2]^+$: 788.41435, found 788.41445.

2-((1*E*,3*E*)-5-((*E*)-1-(3-(1-(4-(((2-Amino-9*H*-purin-6-yl)oxy)methyl)benzyl)-1*H*-1,2,3-triazol-4-yl)propyl)-3,3-dimethylindolin-2-ylidene)penta-1,3-dien-1-yl)-3,3-dimethyl-1-(2-(methylamino)-2-oxoethyl)-3*H*-indol-1-ium 2,2,2-trifluoroacetate (16)

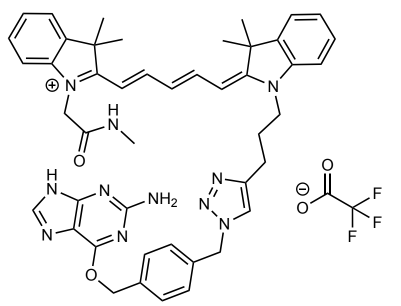

Yield: 9 mg, 42%

^{1}H NMR (400 MHz, CD_3OD): δ = 8.32 – 8.17 (m, 2H), 8.13 (s, 1H), 7.83 (s, 1H), 7.56 (d, *J* = 8.4 Hz, 2H), 7.48 (dd, *J* = 14.6, 6.7 Hz, 2H), 7.37 (dd, *J* = 9.5, 8.1 Hz, 4H), 7.29 – 7.23 (m, 3H), 7.14 (d, *J* = 7.9 Hz, 1H), 6.46 (t, *J* = 12.4 Hz, 1H), 6.23 (d, *J* = 13.5 Hz, 1H), 6.07 (d, *J* = 13.9 Hz, 1H), 5.61(s, 2H), 5.60 (s, 2H), 4.78 (s, 2H), 4.18 (t, *J* = 7.5 Hz, 2H), 2.87 (t, *J* = 7.1 Hz, 2H), 2.80 (s, 3H), 2.20 (t, *J* = 7.5 Hz, 2H), 1.75 (s, 6H), 1.70 (s, 6H) ppm.

^{13}C{^{1}H} NMR (151 MHz, CD_3OD): δ = 175.9, 175.1, 168.4, 161.1, 156.4, 155.2, 148.0, 144.1, 143.3, 142.9, 142.1, 137.5, 137.3, 130.3, 129.8, 129.6, 129.3, 127.0, 126.8, 126.0, 123.9, 123.5, 123.4, 112.3, 111.2, 105.4, 104.0, 69.7, 54.6, 50.9, 50.4, 49.6, 47.3, 44.4, 28.0, 27.8, 27.7, 26.6, 23.3 ppm.

HRMS (ESI) calcd for $[C_{46}H_{50}N_{11}O_2]^+$: 788.41435, found 788.41441.

2-((*E*)-3-((*E*)-1-(3-(1-(4-(((2-Amino-9*H*-purin-6-yl)oxy)methyl)benzyl)-1*H*-1,2,3-triazol-4-yl)propyl)-3,3-dimethyl-5-(trifluoromethyl)indolin-2-ylidene)prop-1-en-1-yl)-1,3-di-methyl-3-(2-(methylamino)-2-oxoethyl)-3*H*-indol-1-ium 2,2,2-tri-fluoroacetate (19)

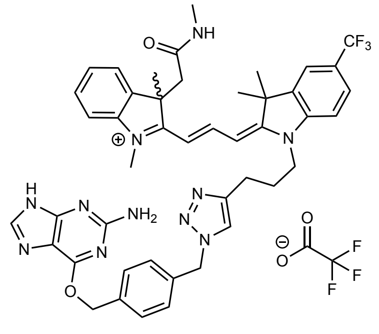

Yield: 16 mg, 73%

^{1}H NMR (400 MHz, CD_3OD): δ = 8.42 (t, *J* = 13.5 Hz, 1H), 8.31 (s, 1H), 7.84 (s, 1H), 7.80 (s, 1H), 7.66 (d, *J* = 8.4 Hz, 1H), 7.57 (d, *J* = 8.1 Hz, 2H), 7.52 (d, *J* = 7.5 Hz, 1H), 7.50 – 7.42 (m, 2H), 7.40 – 7.28 (m, 4H), 6.57 (d, *J* = 13.9 Hz, 1H), 6.37 (d, *J* = 13.0 Hz, 1H), 5.64 (s, 2H), 5.61 (s, 2H), 4.16 (t, *J* = 7.5 Hz, 2H), 3.78 (s, 3H), 3.38 (d, *J* = 15.9 Hz, 1H), 3.22 (d, *J* = 15.7 Hz, 1H), 2.88 (t, *J* = 7.3 Hz, 2H), 2.42 (s, 3H), 2.21 (p, *J* = 7.5 Hz, 2H), 1.76 (s, 3H), 1.76 (s, 3H), 1.70 (s, 3H) ppm.

^{13}C{^{1}H} NMR (151 MHz, CD_3OD): δ = 178.3, 174.1, 170.5, 161.0, 158.5, 153.9, 151.3, 148.0, 146.8, 144.8, 143.3, 142.5, 140.4, 137.5, 137.1, 130.5, 130.3, 129.4, 127.5, 127.3, 125.8 (q, *J* = 271.3 Hz), 123.9, 123.3, 120.7 (q, *J* = 4.1 Hz), 113.3, 111.7, 106.8, 103.4, 70.3, 54.5, 52.9, 49.9, 47.1, 44.3, 32.6, 28.5, 28.4, 27.43, 27.35, 26.0, 23.4 ppm.

HRMS (ESI) calcd for $[C_{45}H_{47}F_3N_{11}O_2]^+$: 830.38608, found 830.38619.

2-((1*E*,3*E*,5*E*)-7-((*E*)-1-(3-(1-(4-(((2-Amino-9*H*-purin-6-yl)oxy)methyl)benzyl)-1*H*-1,2,3-triazol-4-yl)propyl)-3,3-dimethylindolin-2-ylidene)hepta-1,3,5-trien-1-yl)-3-(2-((2,2-difluoroethyl)amino)-2-oxoethyl)-1,3-dimethyl-3*H*-indol-1-ium 2,2,2-trifluoroacetate (20)

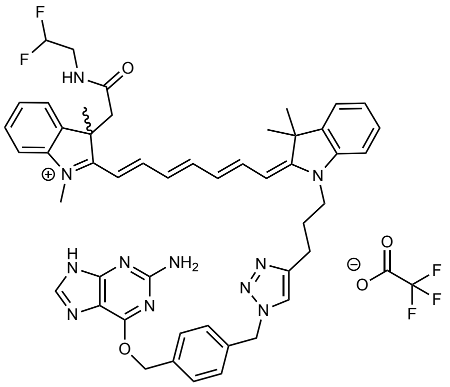

Yield: 12.5 mg, 58%

^{1}H NMR (400 MHz, CD_3CN): δ = 7.96 (s, 1H), 7.79 – 7.66 (m, 2H), 7.61 (s, 1H), 7.53 (d, *J* = 8.1 Hz, 2H), 7.45 – 7.38 (m, 3H), 7.34 – 7.30 (m, 3H), 7.26 – 7.21 (m, 2H), 7.17 (t, *J* = 7.6 Hz, 1H), 7.11 (d, *J* = 7.9 Hz, 1H), 6.76 (t, *J* = 6.0 Hz, 1H), 6.42 (t, *J* = 12.6 Hz, 1H), 6.29 (dd, *J* = 31.8, 13.3 Hz, 2H), 6.04 (d, *J* = 13.5 Hz, 1H), 5.53 (tt, *J* = 55.9, 3.8 Hz, 1H), 5.56 (s, 2H), 5.55 (s, 2H), 4.00 (t, *J* = 7.7 Hz, 2H), 3.55 (s, 3H), 3.31 – 3.17 (m, 2H), 3.13 – 3.07 (m, 2H), 2.80 (t, *J* = 7.3 Hz, 2H), 2.15 – 2.07 (m, 2H), 1.59 (s, 9H) ppm.

^{13}C{^{1}H} NMR (126 MHz, CD_3CN): δ = 173.5, 171.6, 170.0, 160.9, 158.0, 156.2, 154.2, 152.1, 151.0, 147.8, 144.9, 143.6, 141.9, 140.2, 137.5, 136.9, 130.1, 129.7, 129.4, 129.1, 126.4, 125.9, 125.3, 123.2, 123.1, 117.0, 115.1, 113.2, 111.9, 111.3, 105.7, 103.8, 69.6, 54.0, 51.8, 49.7, 47.2, 46.5, 44.1, 41.8 (t, *J* = 20.8 Hz), 32.3, 27.9, 27.4, 27.2, 23.2, 9.0 ppm.

HRMS (ESI) calcd for $[C_{49}H_{52}F_2N_{11}O_2]^+$: 864.42680, found 864.42562.

Supplementary Compounds

3-(Carboxymethyl)-1,3-dimethyl-2-((1*E*,3*E*)-5-((*E*)-1,3,3-trimethyl-5-(trifluoro-methyl)indolin-2-ylidene)penta-1,3-dien-1-yl)-3*H*-indol-1-ium 2,2,2-tri-fluoro-acetate (S1)

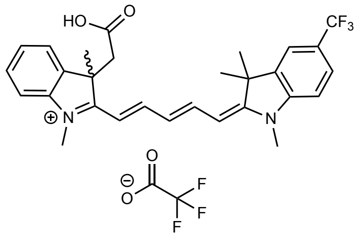

Compound **S1** was synthesized in the same manner as compound **2b** in 56% yield.

^{1}H NMR (400 MHz, CD_3OD): δ = 8.30 (dd, *J* = 14.3, 11.9 Hz, 1H), 8.22 (t, *J* = 13.1 Hz, 1H), 7.71 (s, 1H), 7.66 (dd, *J* = 8.3, 1.1 Hz, 1H), 7.57 (d, *J* = 7.6 Hz, 1H), 7.50 – 7.42 (m, 2H), 7.35 (td, *J* = 7.4, 1.3 Hz, 1H), 7.29 (d, *J* = 8.4 Hz, 1H), 6.66 (t, *J* = 12.4 Hz, 1H), 6.51 (d, *J* = 14.3 Hz, 1H), 6.14 (d, *J* = 13.3 Hz, 1H), 3.75 (s, 3H), 3.69 (d, *J* = 17.6 Hz, 1H), 3.54 (s, 3H), 3.47 (d, *J* = 17.9 Hz, 1H), 1.75 (s, 3H), 1.74 (s, 3H), 1.68 (s, 3H) ppm.

^{13}C{^{1}H} NMR (101 MHz, CD_3OD): δ = 177.4, 172.5, 172.3, 156.4, 153.8, 147.8, 145.0, 142.6, 141.3, 130.1, 127.5, 127.4, 127.3 (q, *J* = 3.6 Hz), 126.5 (q, *J* = 32.6 Hz), 124.6, 123.1, 120.3 (q, *J* = 3.5 Hz), 110.8, 107.4, 103.2, 52.4, 44.9, 32.2, 28.0, 27.9, 27.3 ppm.

HRMS (ESI) calcd for $[C_{29}H_{30}F_3N_2O_2]^+$: 495.22539, found 495.22509.

2-((1*E*,3*E*)-5-((*E*)-3,3-Dimethyl-1-(pent-4-yn-1-yl)-5-(trifluoromethyl)indolin-2-ylidene)penta-1,3-dien-1-yl) 1,3-dimethyl-3-(2-(methylamino)-2-oxoethyl)-3*H*-indol-1-ium 2,2,2-trifluoroacetate (S2)

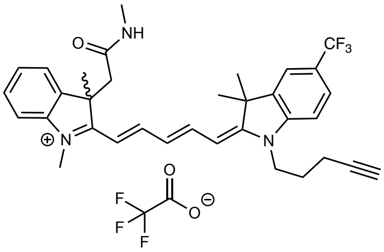

Compound **S2** was synthesized in 94% yield in the same manner as compound **22,** but with compound **9d** as starting material.

^{1}H NMR (400 MHz, CD_3OD): δ = 8.25 (dd, *J* = 14.5, 11.9 Hz, 1H), 8.15 (t, *J* = 13.0 Hz, 1H), 7.70 (s, 1H), 7.65 (d, *J* = 8.2 Hz, 1H), 7.53 – 7.44 (m, 3H), 7.36 (td, *J* = 7.2, 1.4 Hz, 1H), 7.30 (d, *J* = 8.2 Hz, 1H), 6.63 (t, *J* = 12.5 Hz, 1H), 6.56 (d, *J* = 14.5 Hz, 1H), 6.21 (d, *J* = 13.1 Hz, 1H), 4.12 (t, *J* = 7.4 Hz, 2H), 3.78 (s, 3H), 3.50 (d, *J* = 16.0 Hz, 1H), 3.28 (d, *J* = 15.8 Hz, 1H), 2.49 (t, *J* = 2.7 Hz, 1H), 2.43 (s, 3H), 2.36 (td, *J* = 6.7, 2.7 Hz, 2H), 1.97 (p, *J* = 7.1 Hz, 2H), 1.74 (s, 6H), 1.69 (s, 3H) ppm.

$^{13}C\{^1H\}$ NMR (101 MHz, CD_3OD): δ = 178.6, 171.1, 170.6, 156.4, 152.7, 147.3, 144.8, 142.5, 141.1, 130.2, 127.6, 127.6, 127.3 (q, *J* = 4.0 Hz), 126.3 (q, *J* = 32 Hz), 124.6, 123.2, 120.4 (q, J = 3.5 Hz), 113.3, 110.6, 108.3, 102.9, 83.9, 71.3, 53.1, 46.7, 43.2, 32.5, 28.1, 27.1, 26.8, 26.0, 16.5 ppm.
HRMS (ESI) calcd for $[C_{34}H_{37}F_3N_3O]^+$: 560.28832, found 560.28772.

2-((1*E*,3*E*)-5-((*E*)-1-(3-(1-(4-(((2-amino-9*H*-purin-6-yl)oxy)methyl)benzyl)-1*H*-1,2,3-triazol-4-yl)propyl)-3,3-dimethyl-5-(trifluoromethyl)indolin-2-ylidene)-penta-1,3-dien-1-yl)-1,3-dimethyl-3-(2-(methylamino)-2-oxoethyl)-3*H*-indol-1-ium 2,2,2-trifluoroacetate (S3)

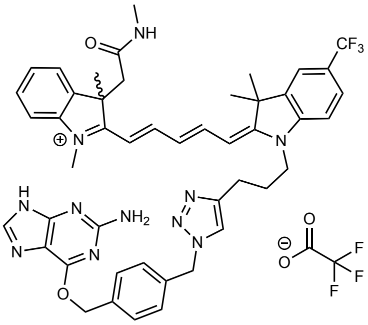

Compound **S3** was synthesized according to the general procedure for click reactions in 53% yield.
^1H NMR (400 MHz, CD_3OD): δ = 8.26 (d, *J* = 12.2 Hz, 1H), 8.22 – 8.19 (m, 1H), 8.11 (t, *J* = 13.0 Hz, 1H), 7.83 (s, 1H), 7.68 (s, 1H), 7.59 – 7.55 (m, 3H), 7.50 (d, *J* = 7.3 Hz, 1H), 7.44 (t, *J* = 7.8 Hz, 2H), 7.40 – 7.33 (m, 3H), 7.16 (d, *J* = 8.4 Hz, 1H), 6.62 – 6.49 (m, 2H), 6.04 (d, *J* = 13.1 Hz, 1H), 5.61 (s, 4H), 4.04 (t, *J* = 7.5 Hz, 2H), 3.78 (s, 3H), 3.54 (d, *J* = 17.8 Hz, 1H), 3.26 (d, *J* = 15.9 Hz, 1H), 2.86 (t, *J* = 7.0 Hz, 2H), 2.42 (s, 3H), 2.16 (p, *J* = 7.2 Hz, 2H), 1.70 (s, 6H), 1.68 (s, 3H) ppm.
HRMS (ESI) calcd for $[C_{47}H_{49}F_3N_{11}O_2]^+$: 856.40228, found 856.40135.

2-((1*E*,3*E*,5*E*)-7-((*E*)-3,3-dimethyl-1-(pent-4-yn-1-yl)indolin-2-ylidene)hepta-1,3,5-trien-1-yl)-3-(2-methoxy-2-oxoethyl)-1,3-dimethyl-3*H*-indol-1-ium 2,2,2-trifluoro-acetate (S4)

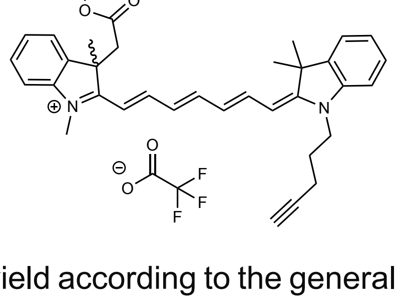

S4 was synthesized in 57% yield according to the general procedure for compounds **9**, but with heating to 100 °C in the microwave for 30 min after addition of the second indoleninium building block.

^{1}H NMR (400 MHz, CD_3OD): δ = 7.90 (td, *J* = 13.2, 4.5 Hz, 2H), 7.58 (t, *J* = 12.8 Hz, 1H), 7.49 – 7.36 (m, 4H), 7.32 – 7.20 (m, 4H), 6.62 – 6.47 (m, 2H), 6.32 (t, *J* = 14.2 Hz, 2H), 4.17 (t, *J* = 7.4 Hz, 2H), 3.64 (s, 3H), 3.51 (d, *J* = 17.3 Hz, 1H), 3.43 (d, *J* = 17.6 Hz, 1H), 3.40 (s, 3H), 2.49 (t, *J* = 2.7 Hz, 1H), 2.36 (td, *J* = 6.6, 2.6 Hz, 2H), 1.99 (p, *J* = 7.1 Hz, 2H), 1.69 (s, 6H), 1.66 (s, 3H) ppm.
$^{13}C\{^1H\}$ NMR (101 MHz, CD_3OD): δ = 173.1, 172.4, 171.3, 157.6, 152.8, 152.3, 145.4, 143.8, 142.2, 140.1, 130.1, 129.7, 127.2, 126.9, 126.2, 125.7, 123.4, 123.1, 119.0, 116.1, 111.9, 111.4, 83.8, 71.3, 52.1, 51.5, 50.1, 45.0, 43.6, 31.7, 28.1, 27.5, 27.2, 16.5 ppm.
HRMS (ESI) calcd for $[C_{35}H_{39}N_2O_2]^+$: 519.30060, found 519.30015.

2-((1*E*,3*E*,5*E*)-7-((*E*)-3,3-dimethyl-1-(pent-4-yn-1-yl)indolin-2-ylidene)hepta-1,3,5-trien-1-yl)-1,3-dimethyl-3-(2-(methylamino)-2-oxoethyl)-3*H*-indol-1-ium 2,2,2-trifluoroacetate (S5)

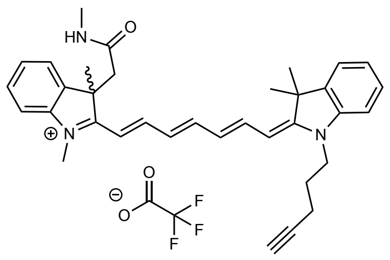

Compound **S5** was synthesized in 96% yield in the same manner as compound **22,** but with compound **S4** as starting material.
^{1}H NMR (400 MHz, CD_3OD): δ = 7.93 (t, *J* = 13.1 Hz, 1H), 7.82 (t, *J* = 13.0 Hz, 1H), 7.54 (t, *J* = 12.6 Hz, 1H), 7.46 – 7.40 (m, 3H), 7.37 – 7.32 (m, 2H), 7.28 (td, *J* = 7.5, 1.1 Hz, 1H), 7.22 – 7.15 (m, 2H), 6.57 (t, *J* = 12.5 Hz, 1H), 6.49 (t, *J* = 12.6 Hz, 1H), 6.41 (d, *J* = 14.2 Hz, 1H), 6.20 (d, *J* = 13.3 Hz, 1H), 4.12 (t, *J* = 7.4 Hz, 2H), 3.68 (s, 3H), 3.31 (d, *J* = 15.4 Hz, 1H), 3.21 (d, *J* = 15.7 Hz, 1H), 2.48 (t, *J* = 2.7 Hz, 1H), 2.44 (s, 3H), 2.35 (td, *J* = 6.7, 2.6 Hz, 2H), 1.97 (p, *J* = 6.7 Hz, 2H), 1.68 (s, 6H), 1.66 (s, 3H) ppm.
$^{13}C\{^1H\}$ NMR (101 MHz, CD_3OD): δ = 175.2, 171.2, 170.8, 157.0, 153.6, 150.8, 145.1, 144.0, 141.9, 140.6, 130.0, 129.6, 127.3, 126.5, 125.1, 123.3, 123.2, 112.4, 110.9, 83.9, 71.2, 52.4, 46.8, 43.3, 32.0, 28.2, 27.4, 27.1, 26.0, 16.6 ppm.
HRMS (ESI) calcd for $[C_{35}H_{40}N_3O]^+$: 518.31659, found 518.31596.

2-((1*E*,3*E*,5*E*)-7-((*E*)-1-(3-(1-(4-(((2-amino-9*H*-purin-6-yl)oxy)methyl)benzyl)-1*H*-1,2,3-triazol-4-yl)propyl)-3,3-dimethylindolin-2-ylidene)hepta-1,3,5-trien-1-yl)-1,3-dimethyl-3-(2-(methylamino)-2-oxoethyl)-3*H*-indol-1-ium 2,2,2-trifluoro-acetate (S6)

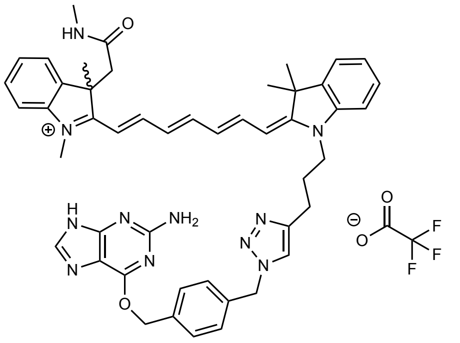

Compound **S6** was synthesized according to the general procedure for Click reactions with 43% yield.

^{1}H NMR (400 MHz, CD_3OD): δ = 8.21 (s, 1H), 7.91 (t, *J* = 13.3 Hz, 1H), 7.81 (s, 1H), 7.77 – 7.72 (m, 1H), 7.57 (d, *J* = 8.2 Hz, 2H), 7.44 (d, *J* = 7.8 Hz, 2H), 7.42 – 7.35 (m, 4H), 7.34 – 7.25 (m, 3H), 7.14 (t, *J* = 7.4 Hz, 1H), 7.07 (d, *J* = 7.9 Hz, 1H), 6.52 (t, *J* = 12.6 Hz, 1H), 6.44 – 6.35 (m, 2H), 6.04 (d, *J* = 13.4 Hz, 1H), 5.62 (s, 2H), 5.60 (s, 2H), 4.04 (t, *J* = 7.2 Hz, 2H), 3.67 (s, 3H), 3.46 (d, *J* =15.8 Hz, 1H), 3.20 (d, *J* = 15.7 Hz, 1H), 2.85 (t, *J* = 7.2 Hz, 2H), 2.43 (s, 3H), 2.16 (p, *J* = 7.1 Hz, 2H), 1.65 (s, 3H), 1.64 (s, 6H) ppm.

HRMS (ESI) calcd for $[C_{48}H_{52}N_{11}O_2]^+$: 814.43000, found 814.43016.

2-((1*E*,3*E*)-5-((*E*)-3,3-dimethyl-1-(pent-4-yn-1-yl)-5-(trifluoromethyl)indolin-2-ylidene)penta-1,3-dien-1-yl)-1-(2-hydroxyethyl)-3,3-dimethyl-3*H*-indol-1-ium 2,2,2-trifluoroacetate (S7)

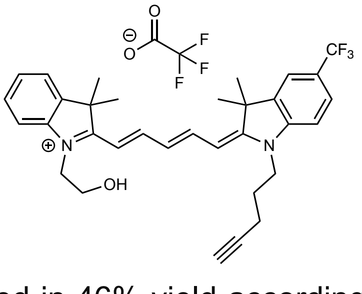

Compound **S7** was synthesized in 46% yield according to the general procedure for compounds **2**, but with heating to 110 °C in the microwave for 30 min after addition of the second indoleninium building block.

^{1}H NMR (400 MHz, CD_3OD): δ = 8.36 (dd, *J* = 14.2, 11.9 Hz, 1H), 8.21 (t, *J* = 13.0 Hz, 1H), 7.73 (s, 1H), 7.66 (d, *J* = 8.4 Hz, 1H), 7.57 (d, *J* = 7.3 Hz, 1H), 7.47 (d, *J* = 3.7 Hz, 2H), 7.40 – 7.32 (m, 2H), 6.67 – 6.58 (m, 2H), 6.26 (d, *J* = 13.1 Hz, 1H), 4.37 (t, *J* = 5.3 Hz, 2H), 4.15 (t, *J* = 7.4 Hz, 2H), 3.98 (t, *J* = 5.3 Hz, 2H), 2.49 (t, *J* = 2.6 Hz,

1H), 2.37 (td, *J* = 6.6, 2.7 Hz, 2H), 1.98 (p, *J* = 6.7 Hz, 2H), 1.78 (s, 6H), 1.75 (s, 6H) ppm.

$^{13}C\{^1H\}$ NMR (101 MHz, CD_3OD): δ = 179.2, 171.6, 157.3, 153.7, 147.1, 143.6, 143.4, 142.7, 129.8, 127.8, 127.6, 127.3 (q, *J* = 3.9 Hz), 126.6 (q, *J* = 32.8 Hz), 124.6, 123.5, 120.4 (q, *J* = 3.9 Hz), 113.7, 110.9, 108.0, 103.3, 83.9, 71.3, 60.2, 51.7, 43.4, 28.0, 27.6, 26.9, 16.5 ppm.

HRMS (ESI) calcd for $[C_{33}H_{36}F_3N_2O]^+$: 533.27742, found 533.27693.

2-((1*E*,3*E*)-5-((*E*)-1-(3-(1-(4-(((2-amino-9*H*-purin-6-yl)oxy)methyl)benzyl)-1*H*-1,2,3-triazol-4-yl)propyl)-3,3-dimethyl-5-(trifluoromethyl)indolin-2-ylidene)-penta-1,3-dien-1-yl)-1-(2-hydroxyethyl)-3,3-dimethyl-3*H*-indol-1-ium 2,2,2-trifluoroacetate (S8)

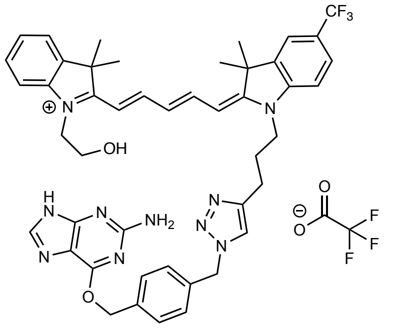

Compound **S8** was synthesized according to the general procedure for Click reactions with 58% yield.

^1H NMR (400 MHz, CD_3OD): δ = 8.34 (dd, *J* = 14.1, 11.8 Hz, 1H), 8.19 (t, *J* = 13.0 Hz, 2H), 7.83 (s, 1H), 7.71 (d, *J* = 2.0 Hz, 1H), 7.61 – 7.54 (m, 4H), 7.48 – 7.44 (m, 2H), 7.39 – 7.35 (m, 3H), 7.20 (d, *J* = 8.4 Hz, 1H), 6.58 (dd, *J* = 13.5, 10.0 Hz, 2H), 6.12 (d, *J* = 13.1 Hz, 1H), 5.61 (s, 4H), 4.36 (t, *J* = 5.3 Hz, 2H), 4.08 (t, *J* = 7.6 Hz, 2H), 3.98 (t, *J* = 5.3 Hz, 2H), 2.86 (t, *J* = 7.3 Hz, 2H), 2.17 (p, *J* = 7.6 Hz, 2H), 1.77 (s, 6H), 1.72 (s, 6H) ppm.

HRMS (ESI) calcd for $[C_{46}H_{48}F_3N_{10}O_2]^+$: 829.39083, found 829.39070.

References

1. Gibson, D. G. *et al.* Enzymatic assembly of DNA molecules up to several hundred kilobases. *Nat. Methods* **6**, 343–345 (2009).
2. Kim, I. K. & Erickson, K. L. Models for uleine-alkaloid biogenesis. *Tetrahedron* **27**, 3979–3991 (1971).
3. Owens, E. A., Bruschi, N., Tawney, J. G. & Henary, M. A microwave-assisted and environmentally benign approach to the synthesis of near-infrared fluorescent pentamethine cyanine dyes. *Dyes Pigm.* **113**, 27–37 (2015).
4. Wolf, N., Kersting, L., Herok, C., Mihm, C. & Seibel, J. High-yielding water-soluble asymmetric cyanine dyes for labeling applications. *J. Org. Chem.* **85**, 9751–9760 (2020).
5. Trost, B. M., Breder, A. & Kai, B. Atom-Economical Synthesis of Functionalized Cycloalkanes via Catalytic Redox Cycloisomerization of Propargyl Alcohols. *Org. Lett.* **14**, 1708–1711 (2012).
6. Li, H., Luan, Z.-J., Zheng, G.-W. & Xu, J.-H. Efficient Synthesis of Chiral Indolines using an Imine Reductase from Paenibacillus lactis. *Adv. Synth. Catal.* **357**, 1692–1696 (2015).
7. Keppler, A. *et al.* A general method for the covalent labeling of fusion proteins with small molecules in vivo. *Nat. Biotechnol* **21**, 86–89 (2003).
8. Meng, G. *et al.* Modular click chemistry libraries for functional screens using a diazotizing reagent. *Nature* **574**, 86–89 (2019).

NMR spectra

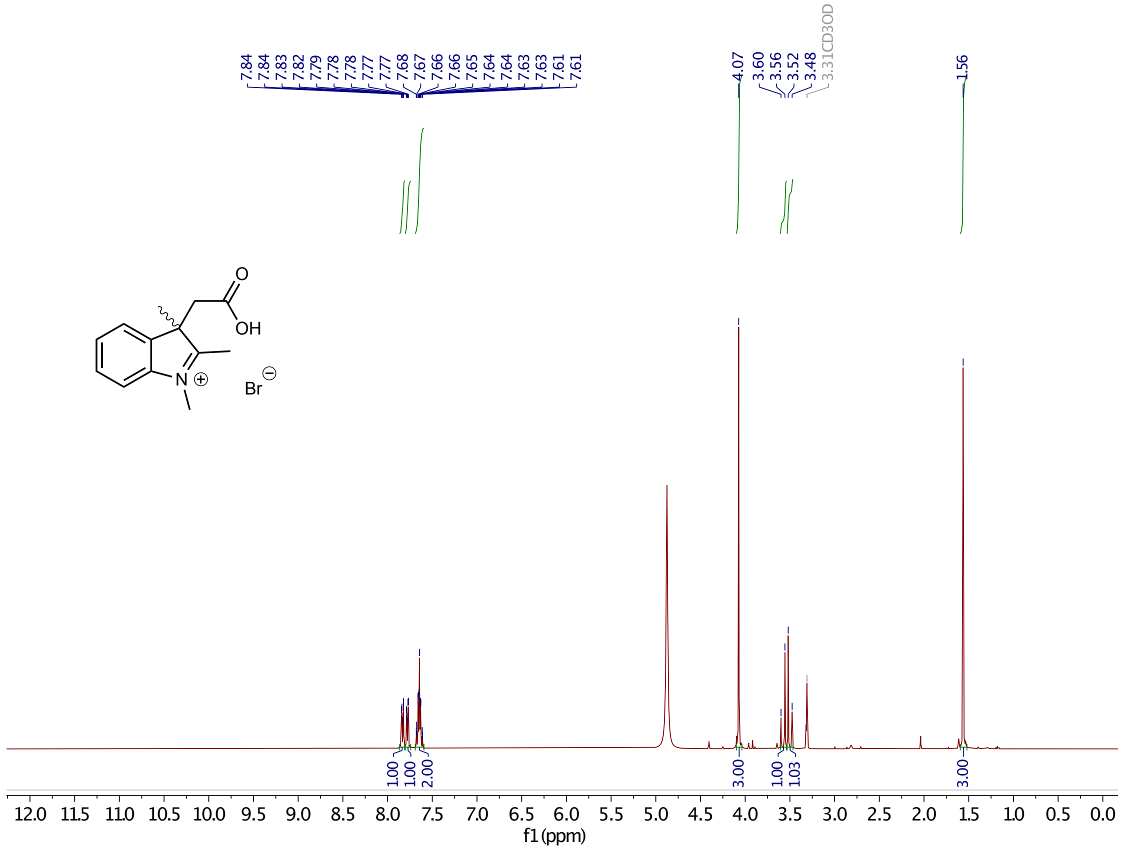

Figure 6. ^{1}H-NMR of **6.**

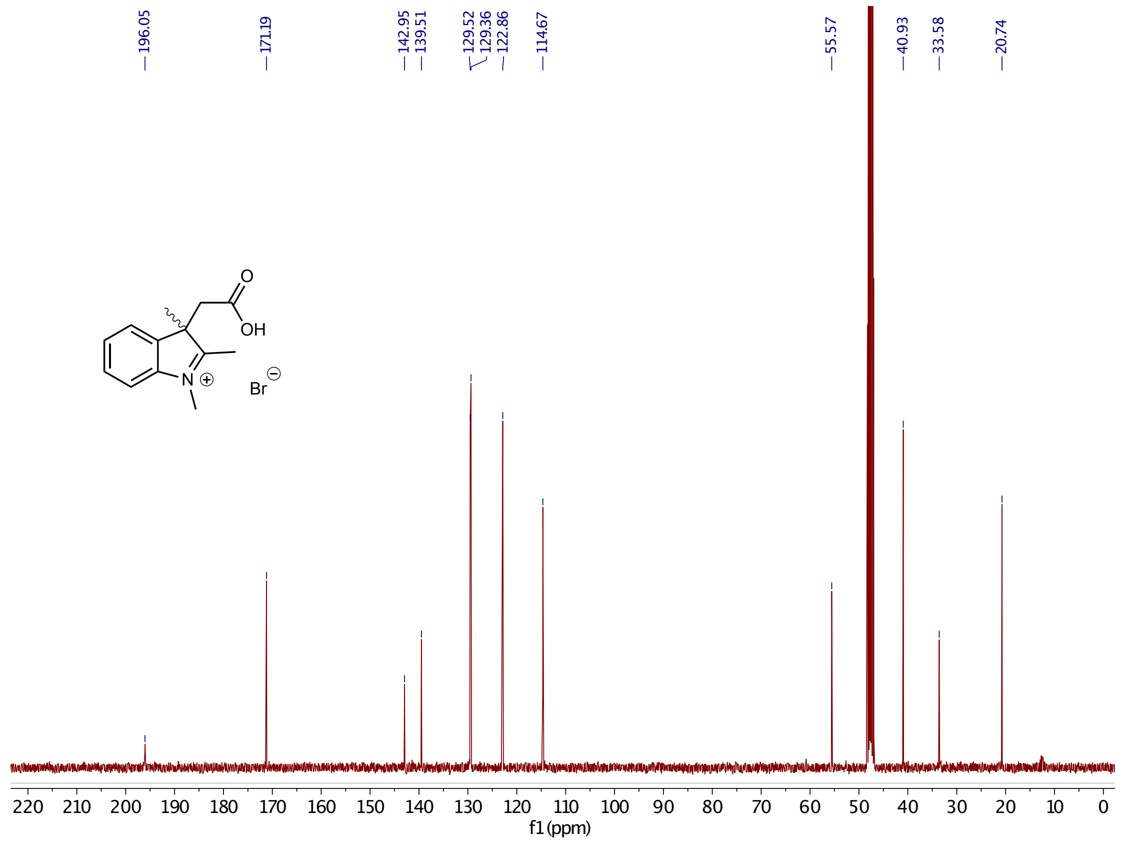

Figure 7. ^{13}C-NMR of **6**.

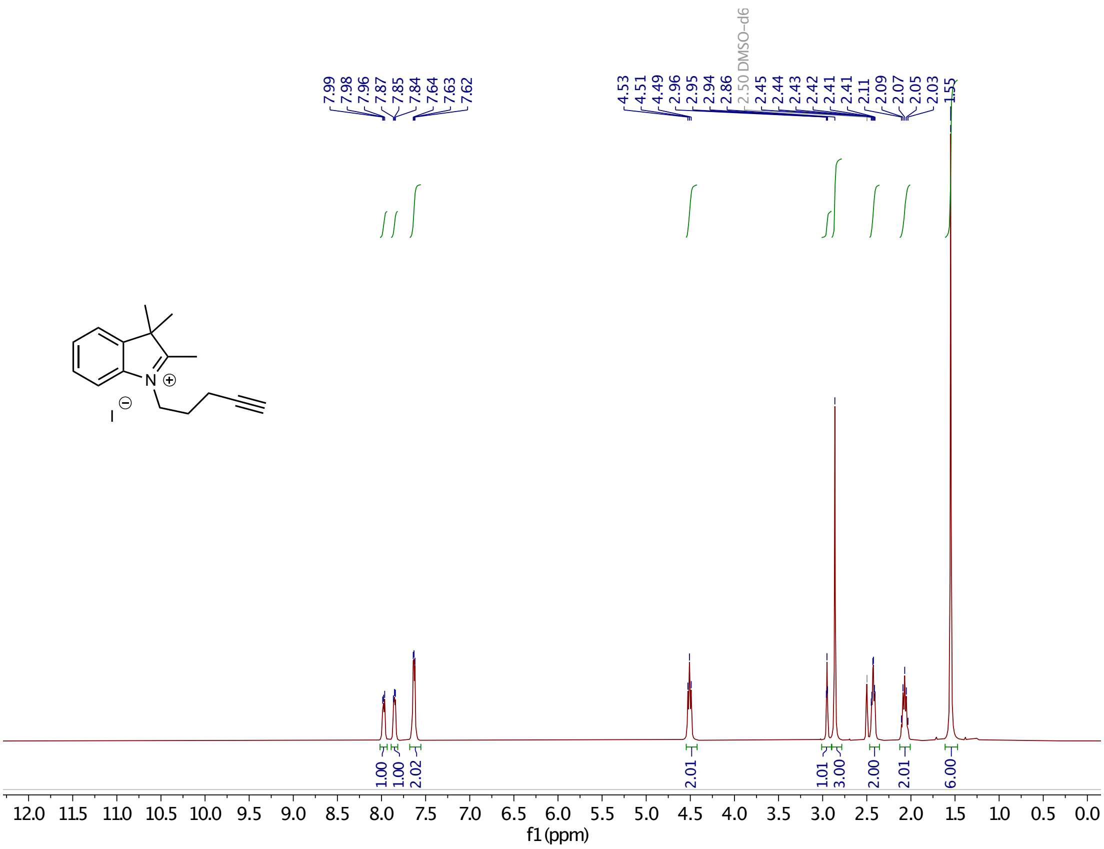

Figure 8. ^{1}H-NMR of **7c**.

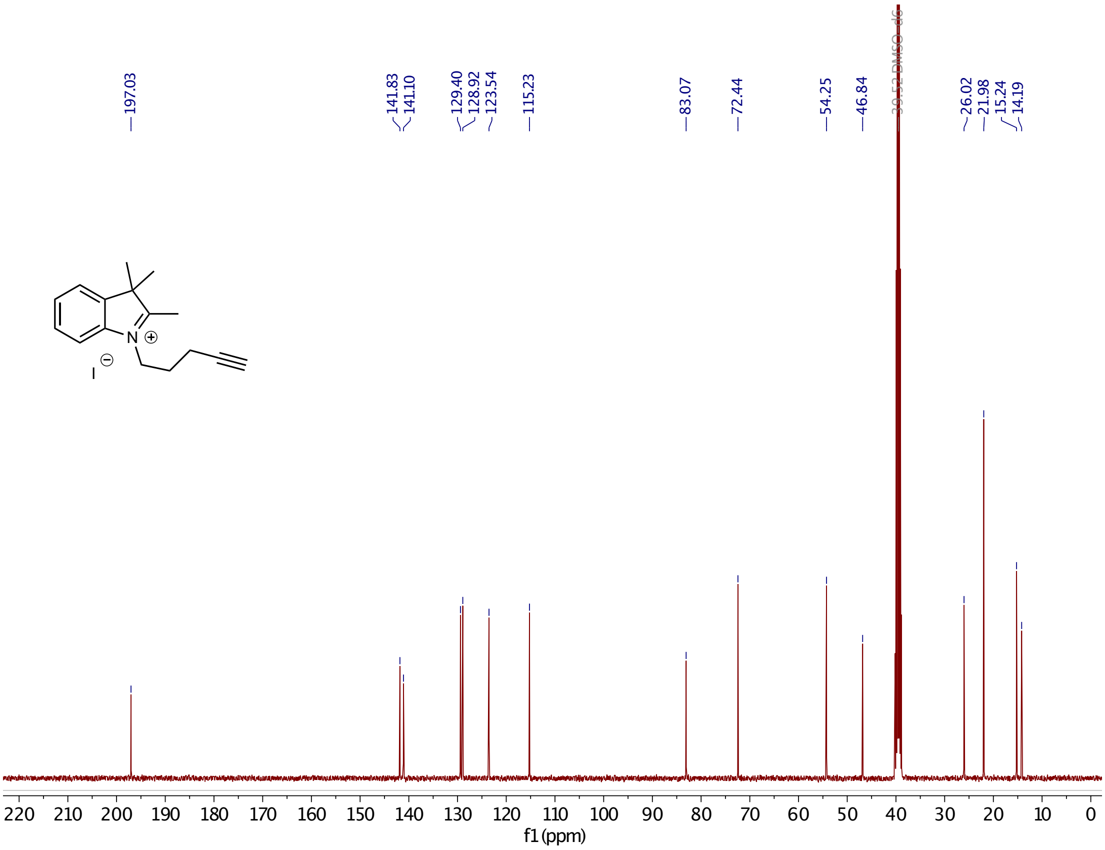

Figure 9. ^{13}C-NMR of **7c**.

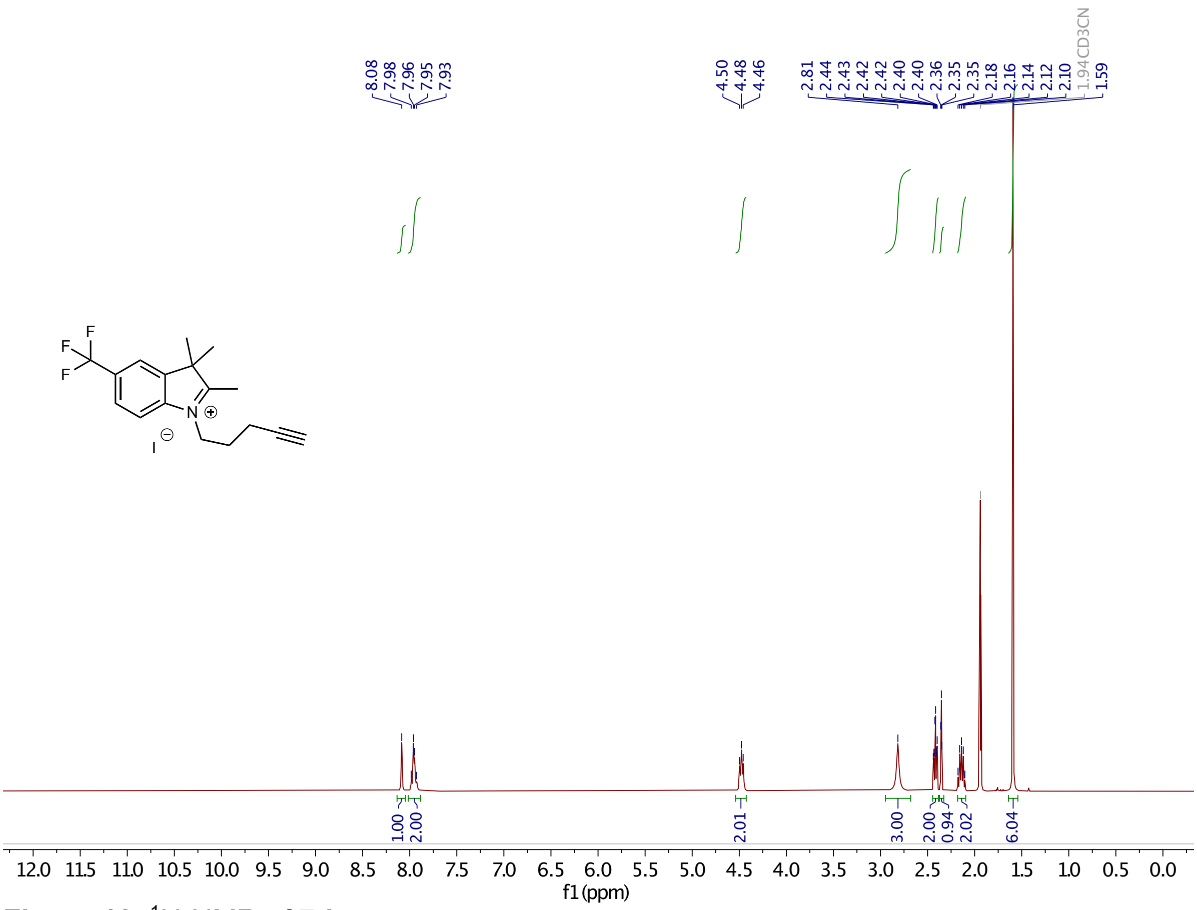

Figure 10. ^{1}H-NMR of **7d**.

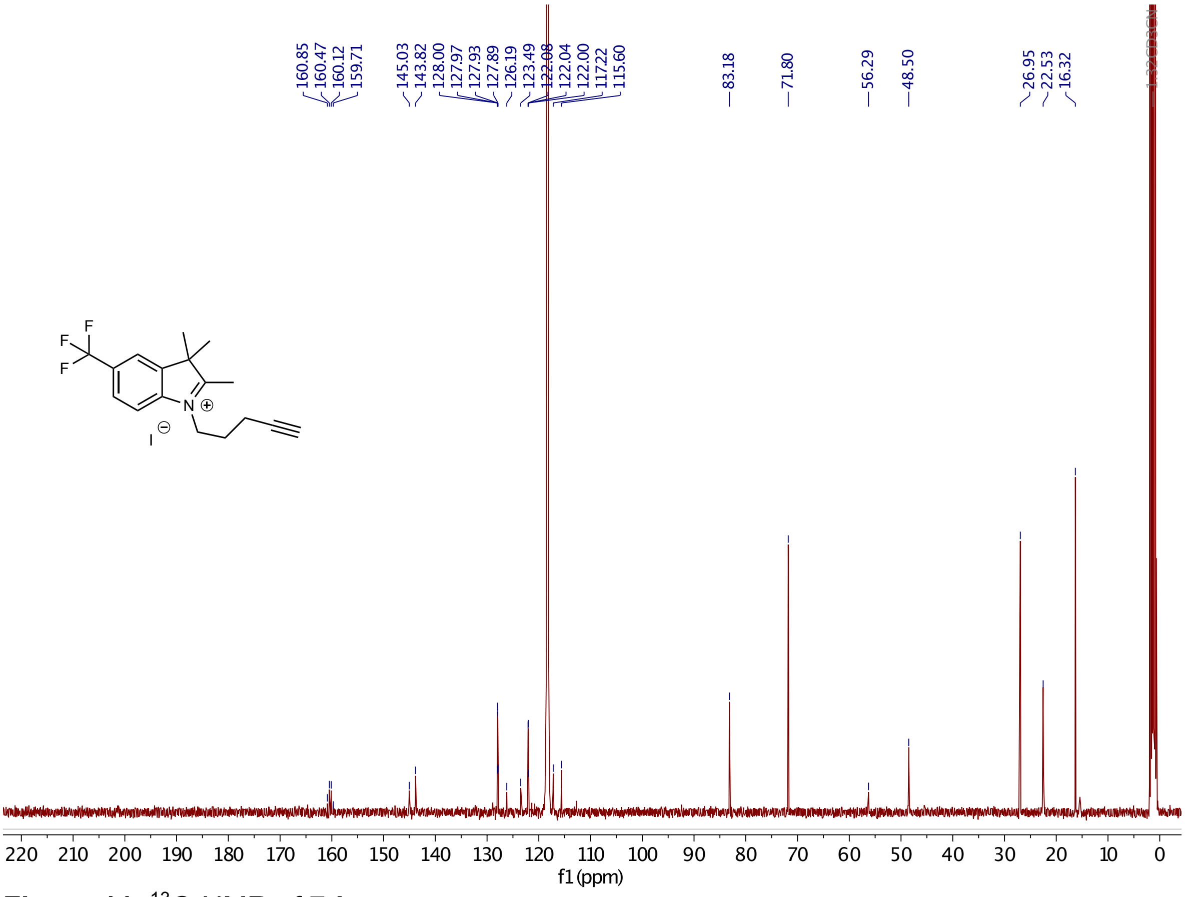

Figure 11. ^{13}C-NMR of **7d**.

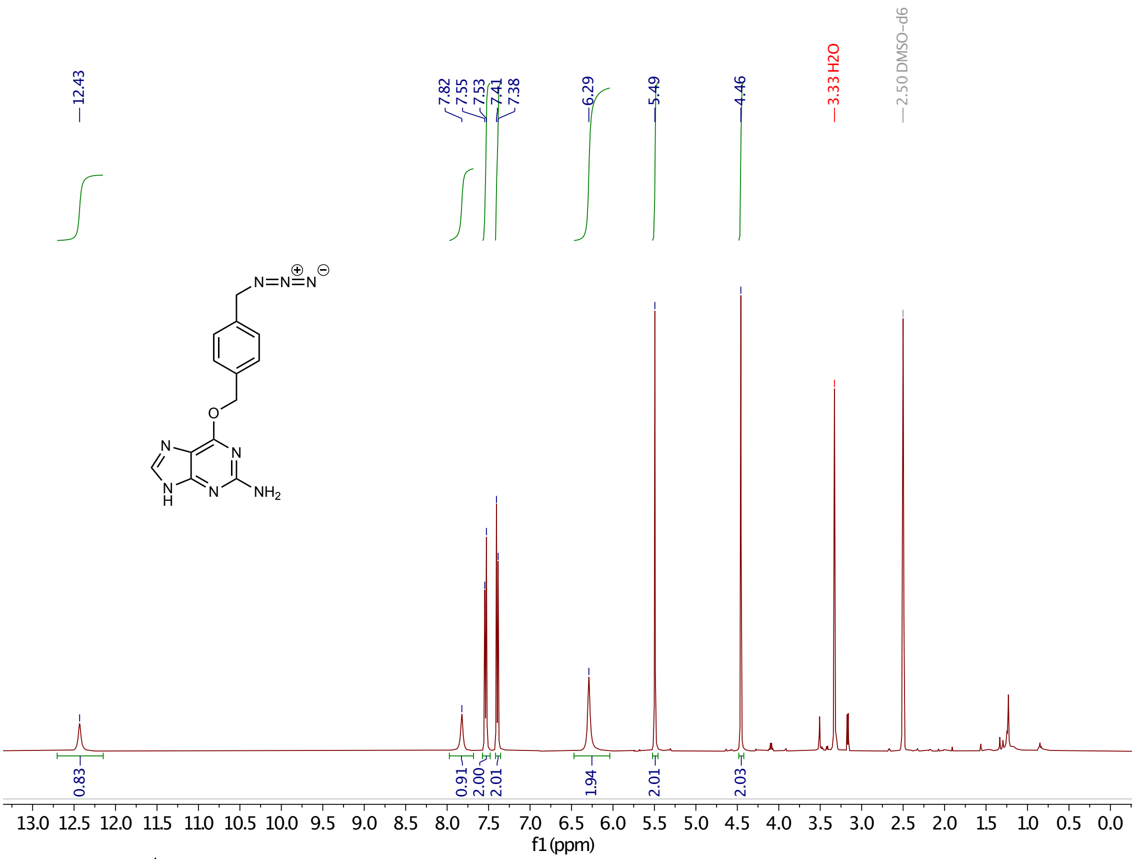

Figure 12. ^{1}H-NMR of **10**.

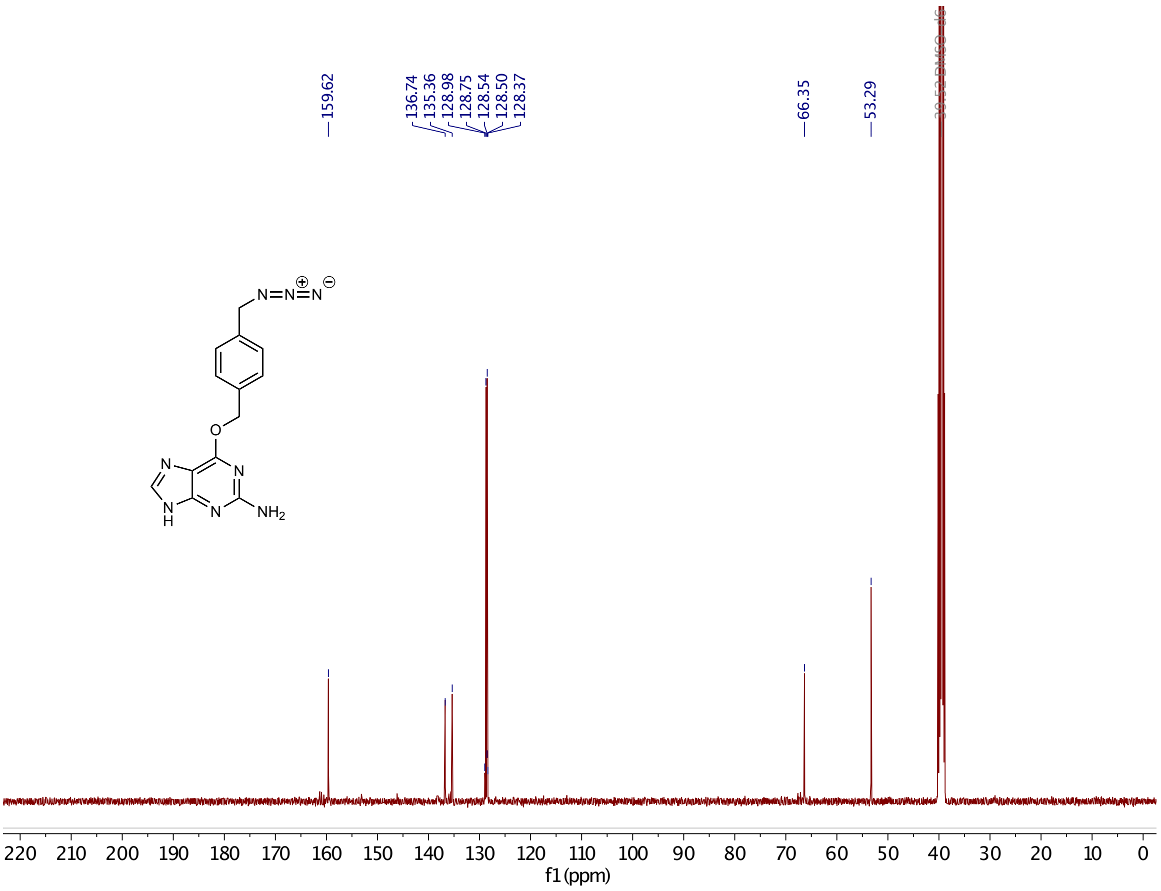

Figure 13. ^{13}C-NMR of **10**.

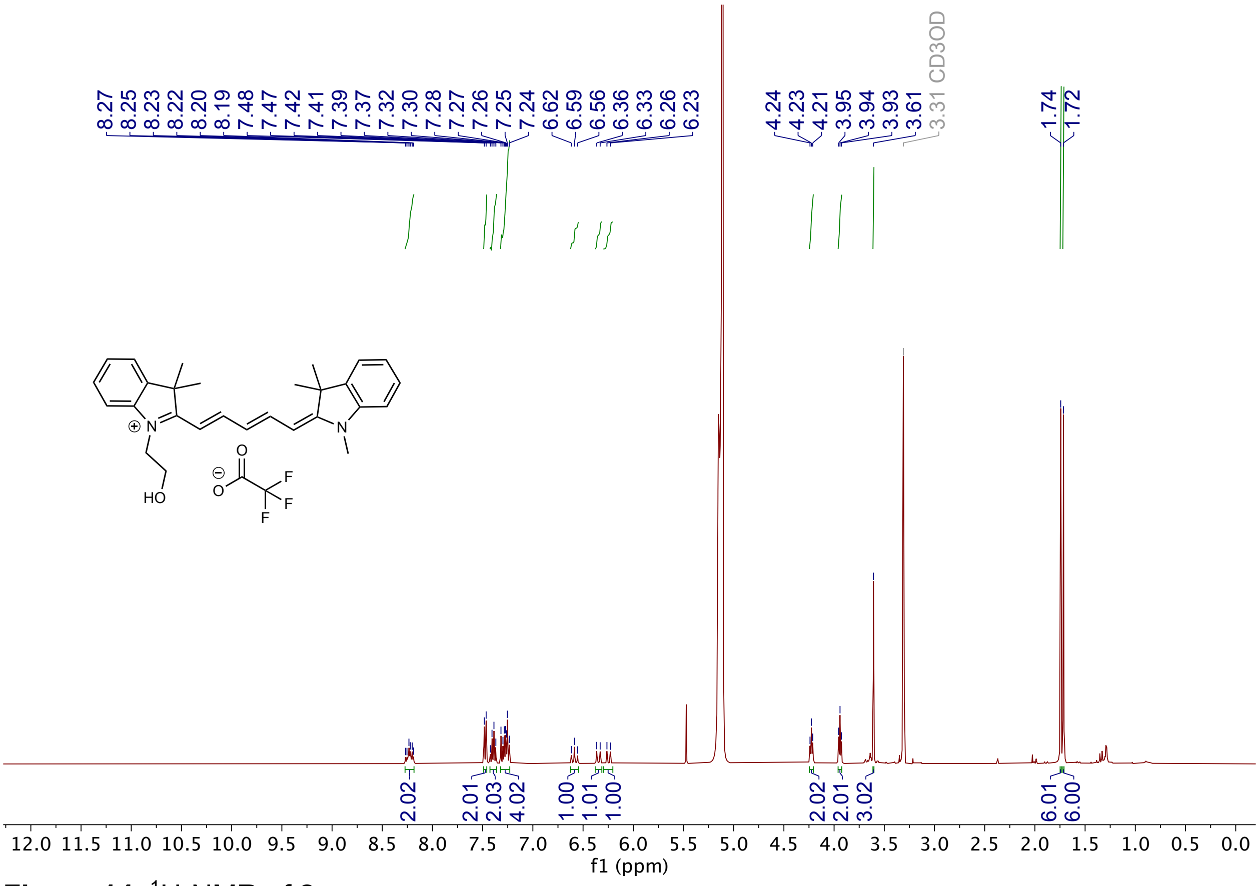

Figure 14. ^{1}H-NMR of **2a**.

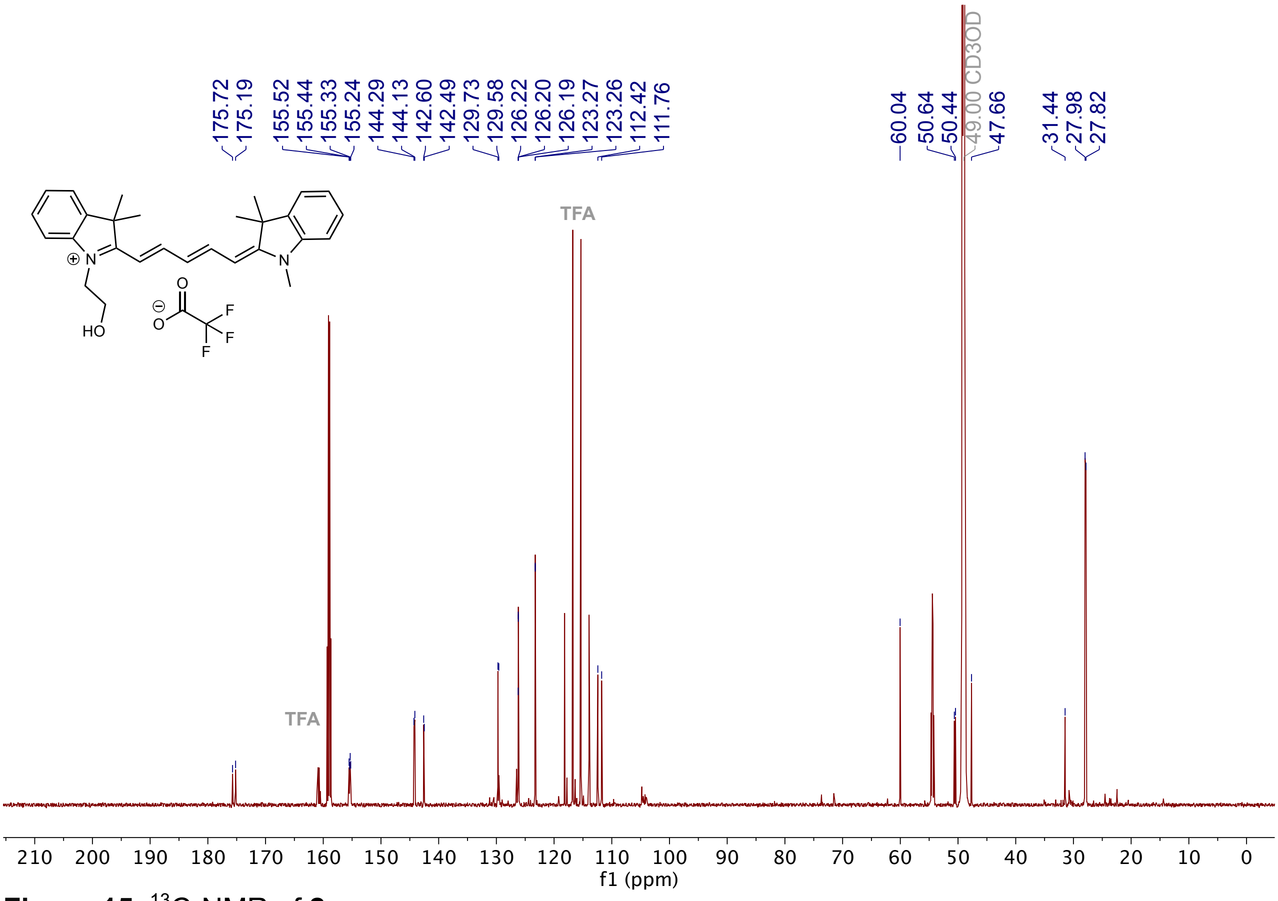

Figure 15. ^{13}C-NMR of **2a**.

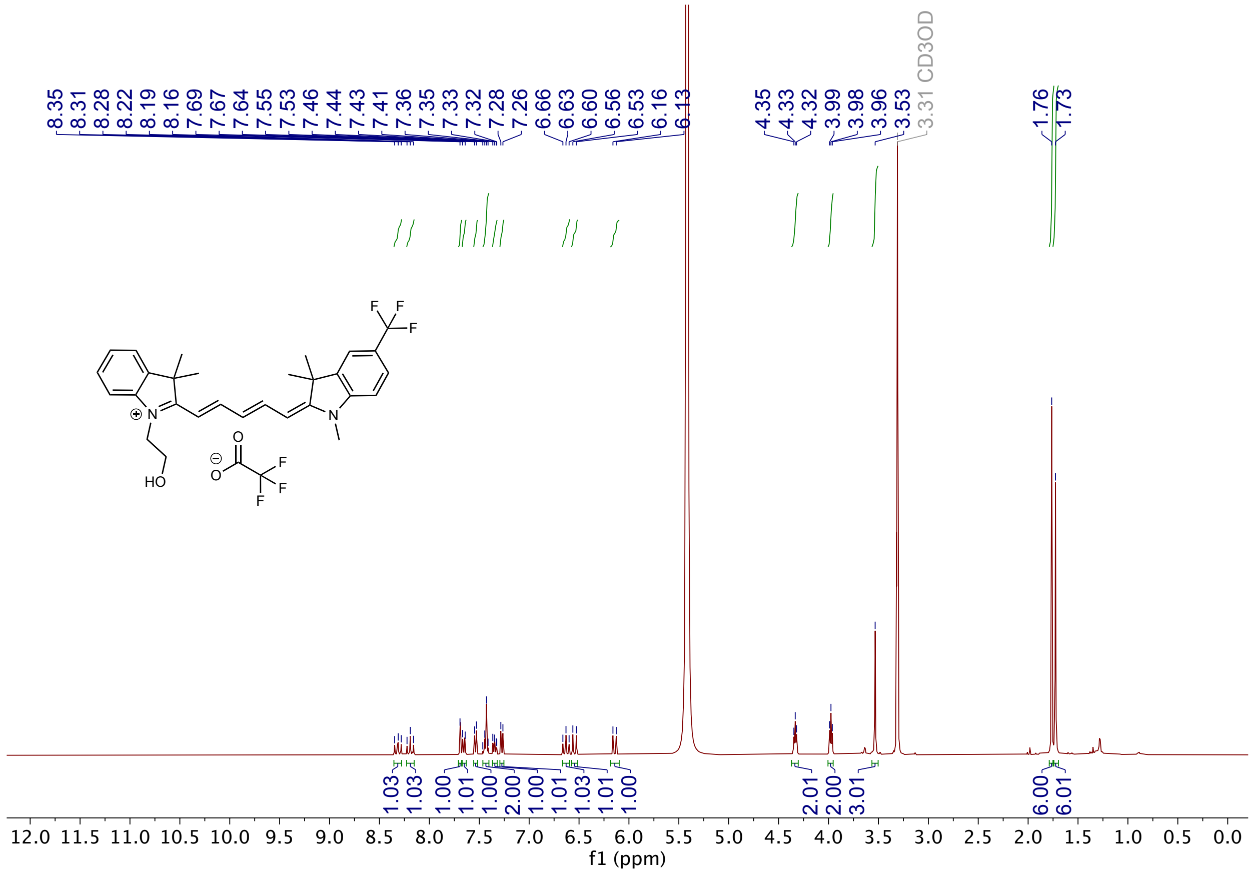

Figure 16. ^{1}H-NMR of **2b**.

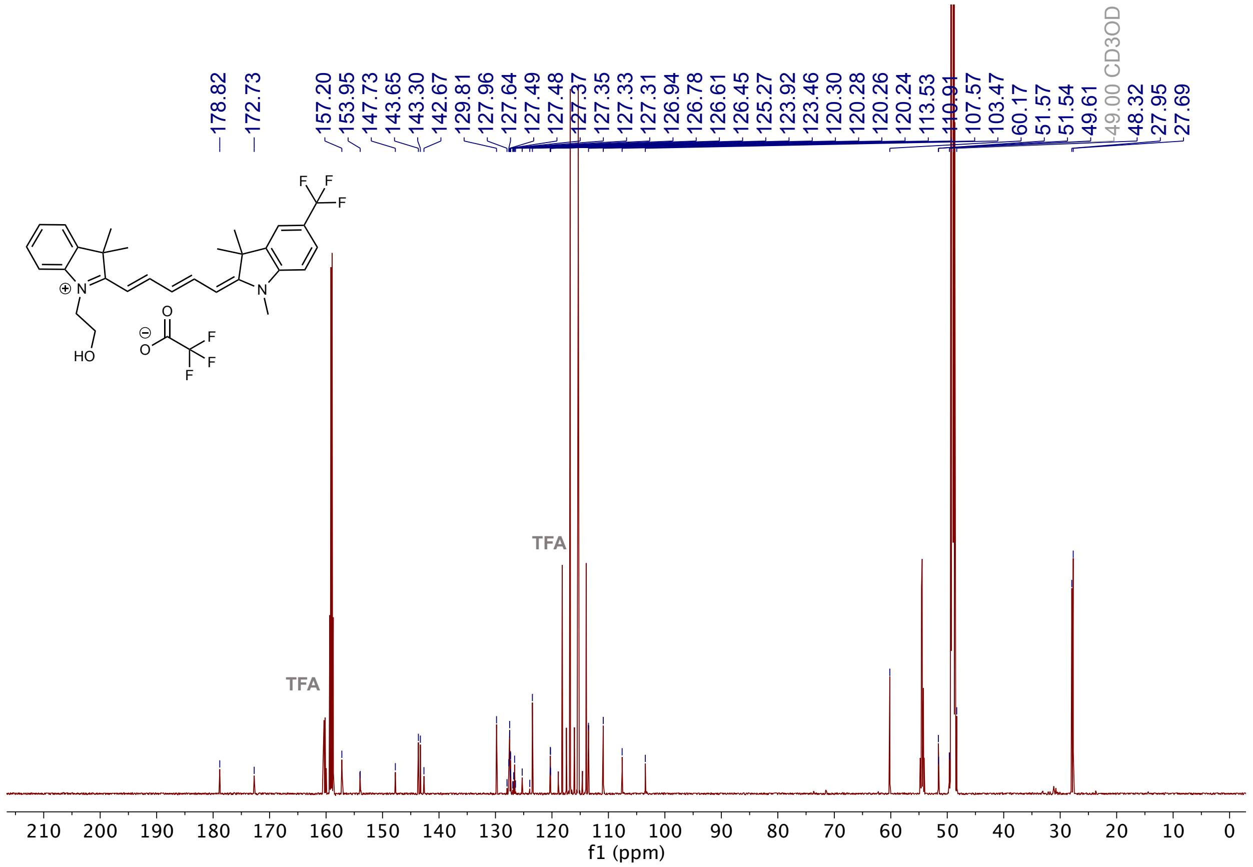

Figure 17. ^{13}C-NMR of **2b**.

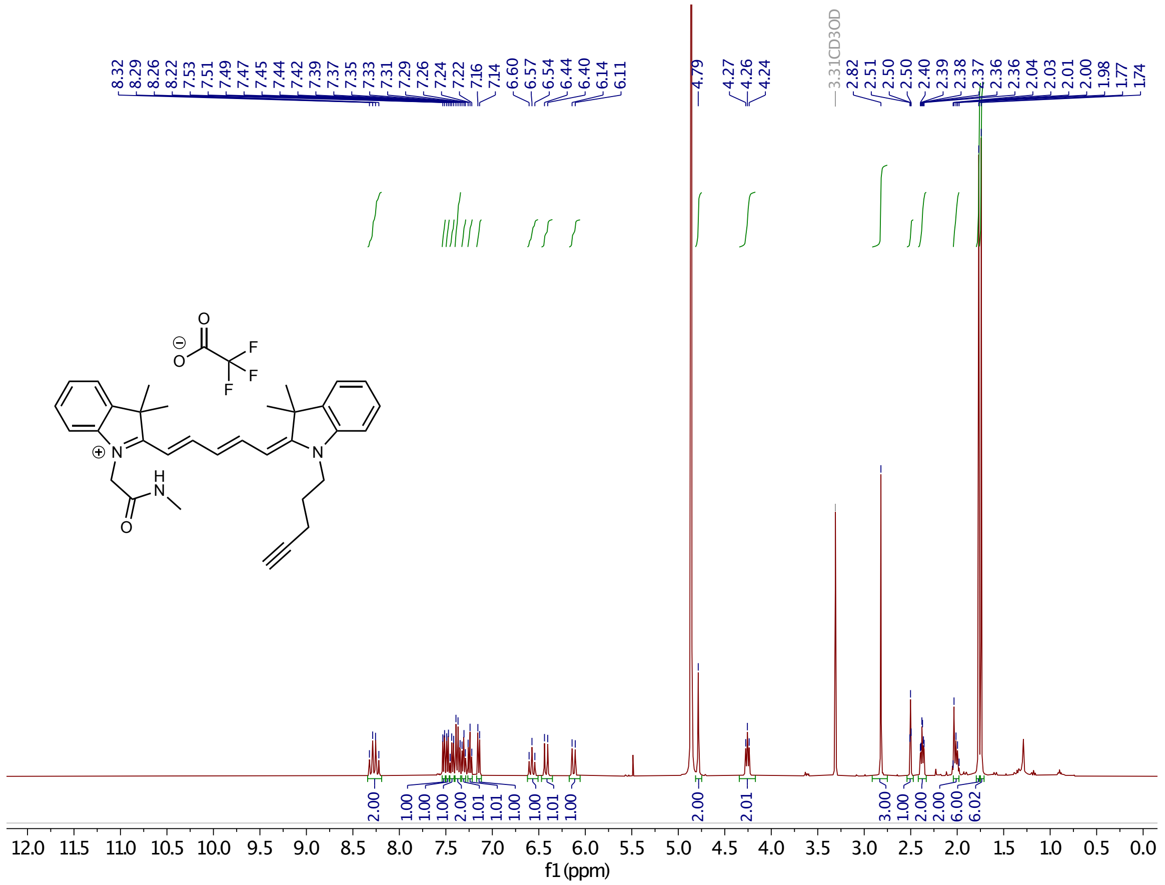

Figure 18. ^{1}H-NMR of **14**.

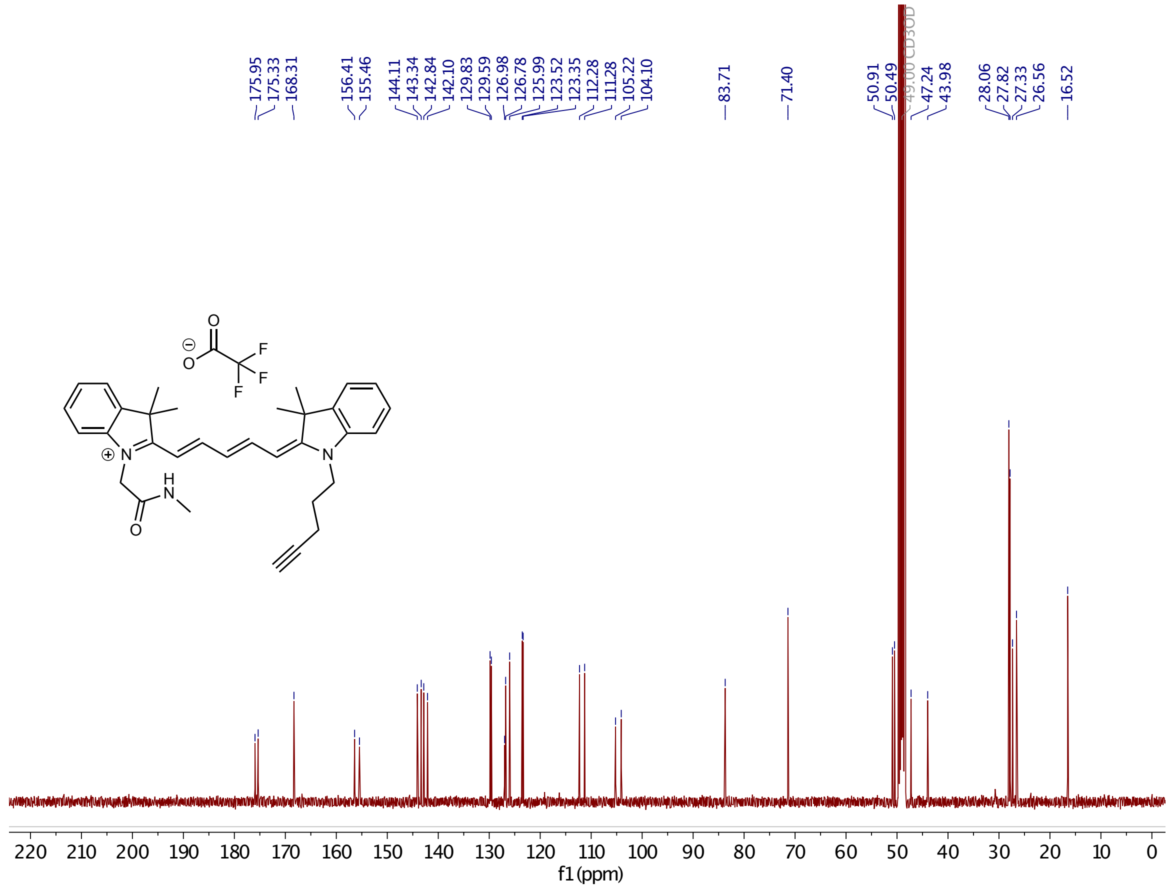

Figure 19. ^{13}C-NMR of **14**.

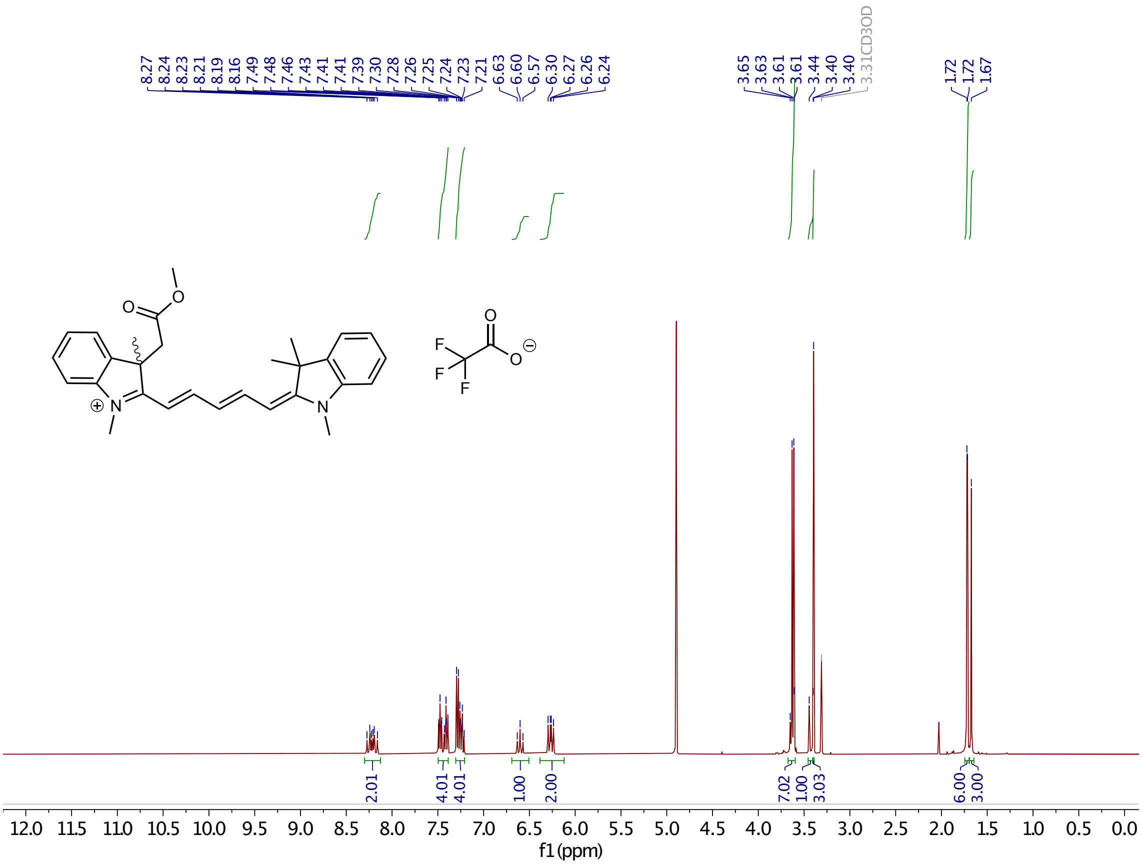

Figure 20. ^{1}H-NMR of **9a**.

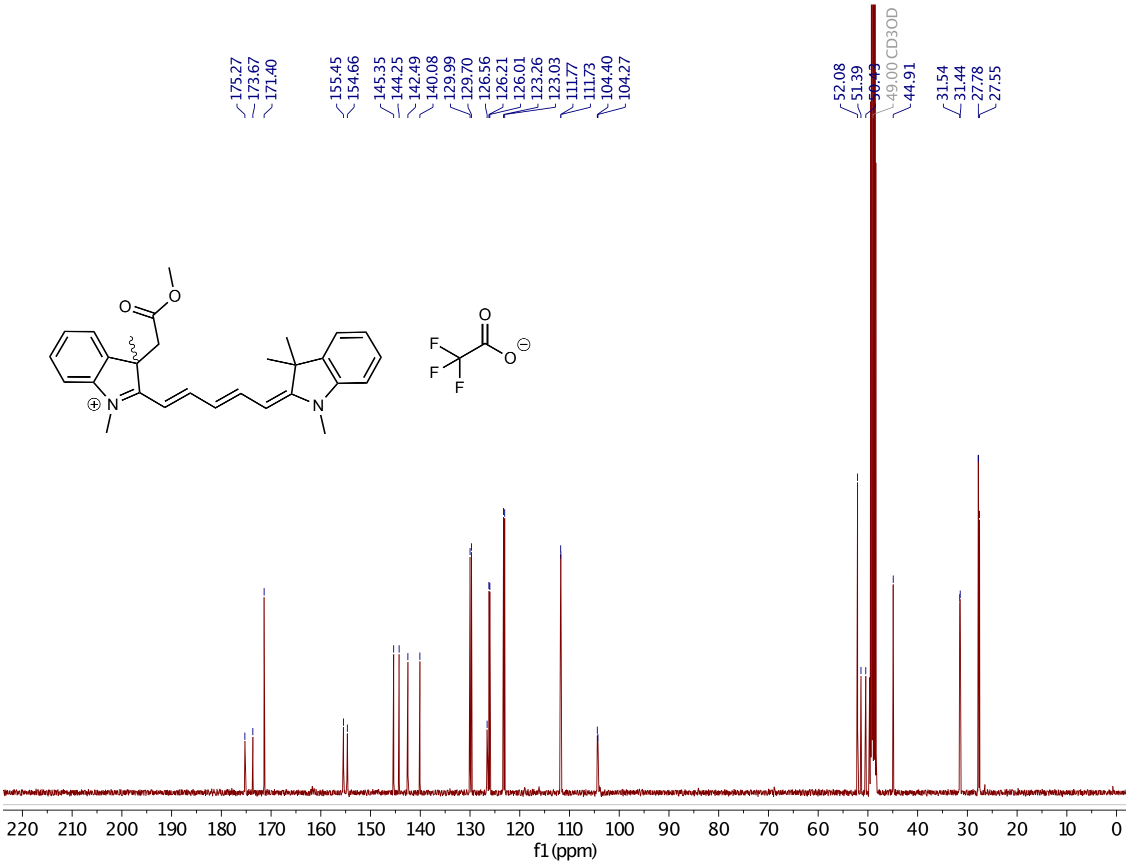

Figure 21. ^{13}C-NMR of **9a**.

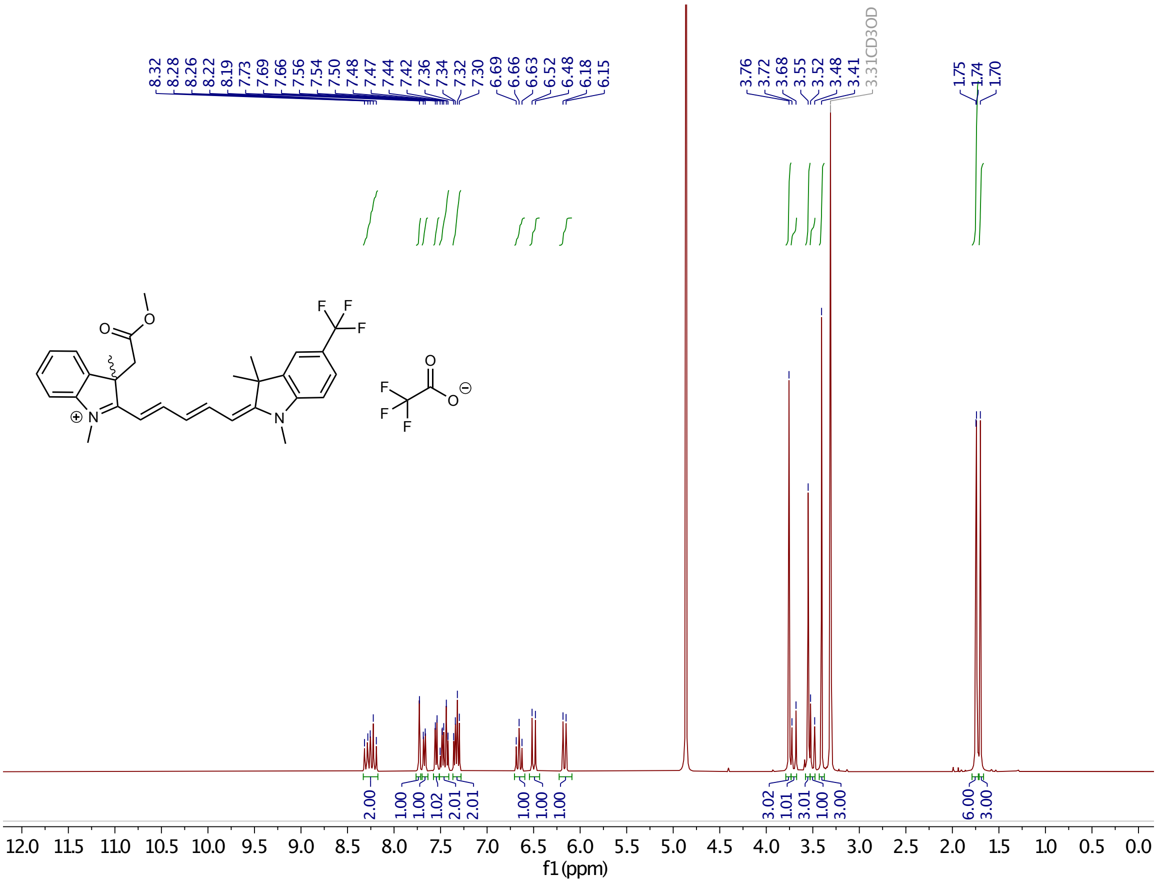

Figure 22. ^{1}H-NMR of **9b**

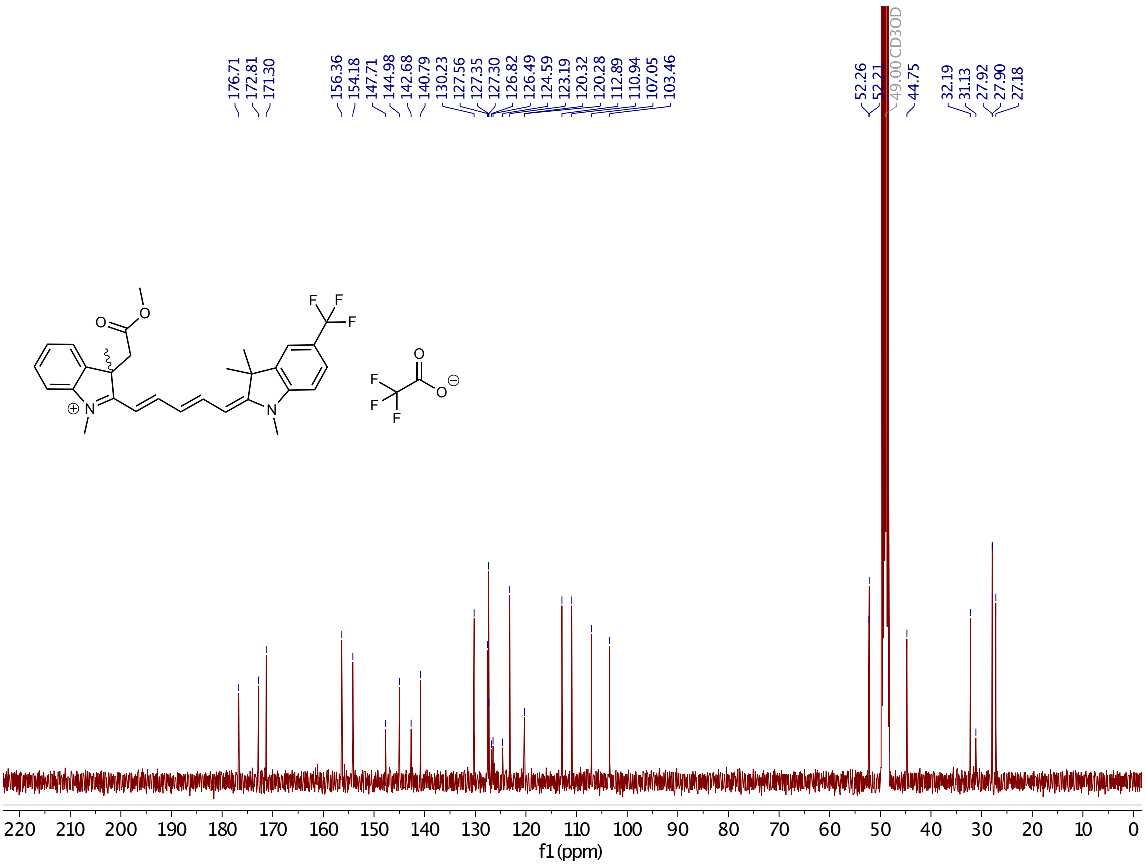

Figure 23. ^{13}C-NMR of **9b**.

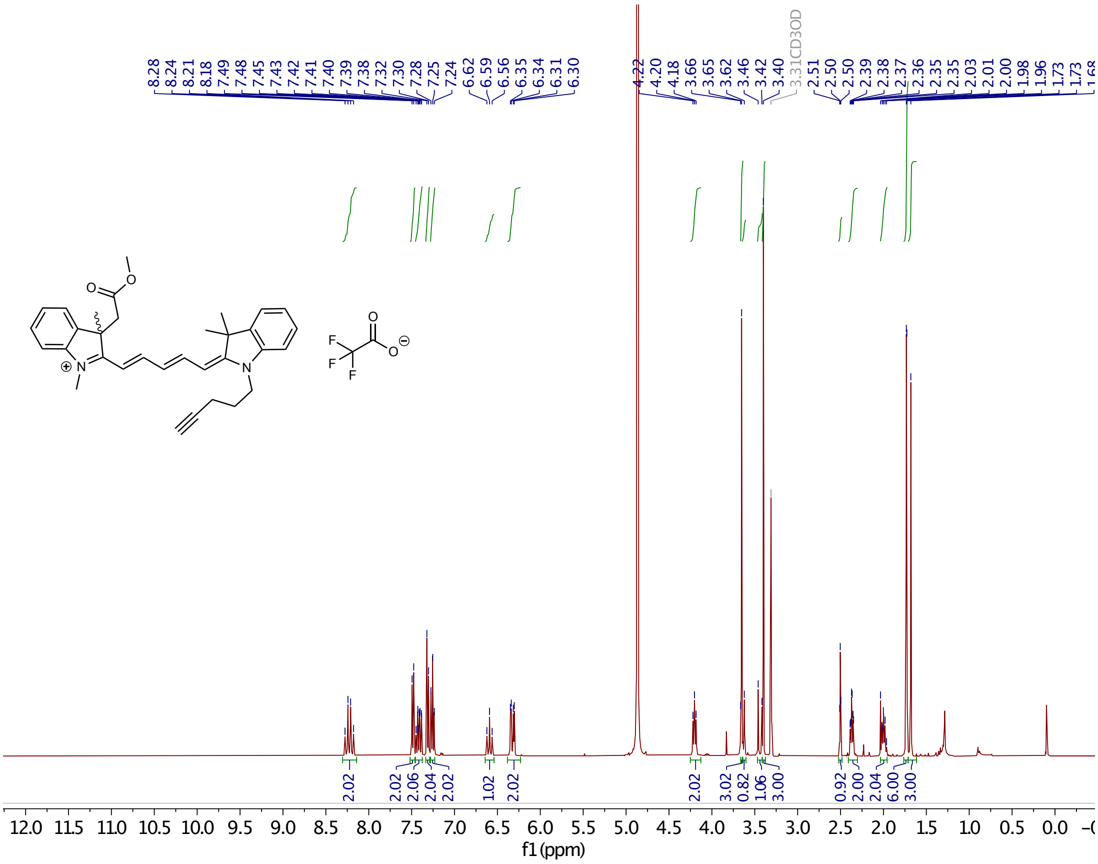

Figure 24. ^{1}H-NMR of **9c**.

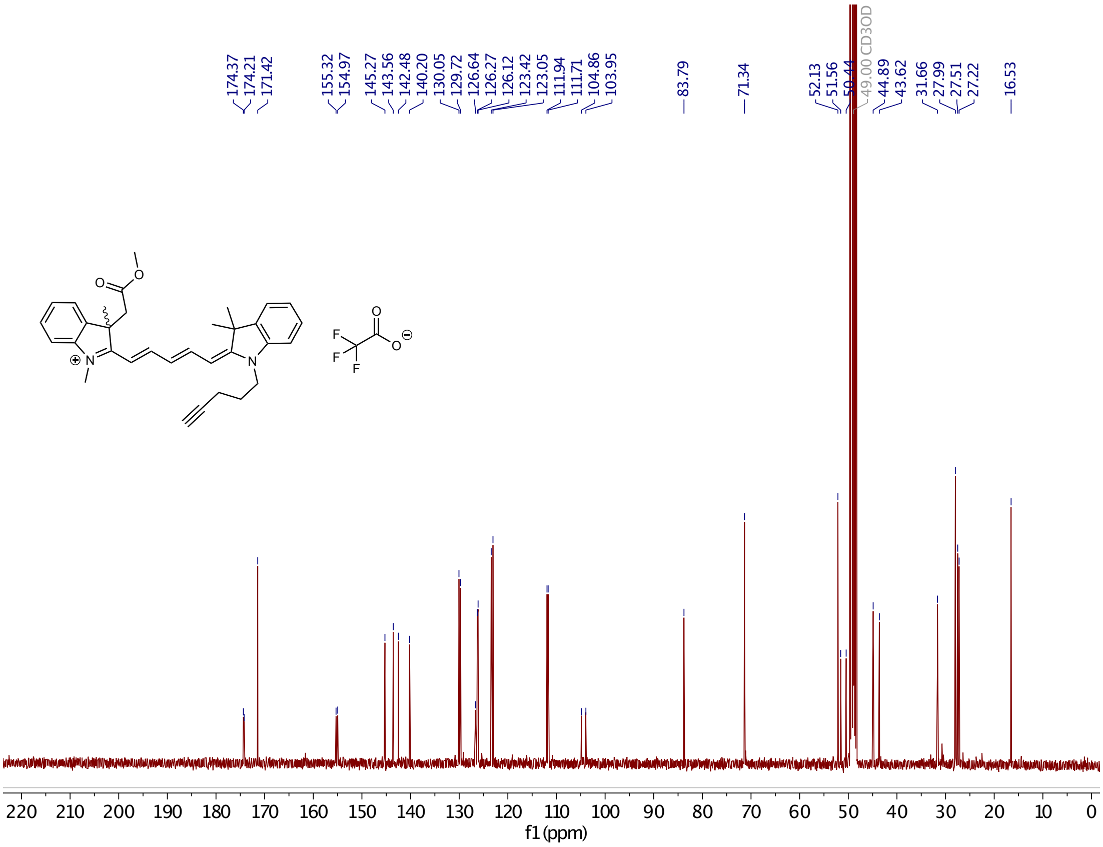

Figure 25. ^{13}C-NMR of **9c**.

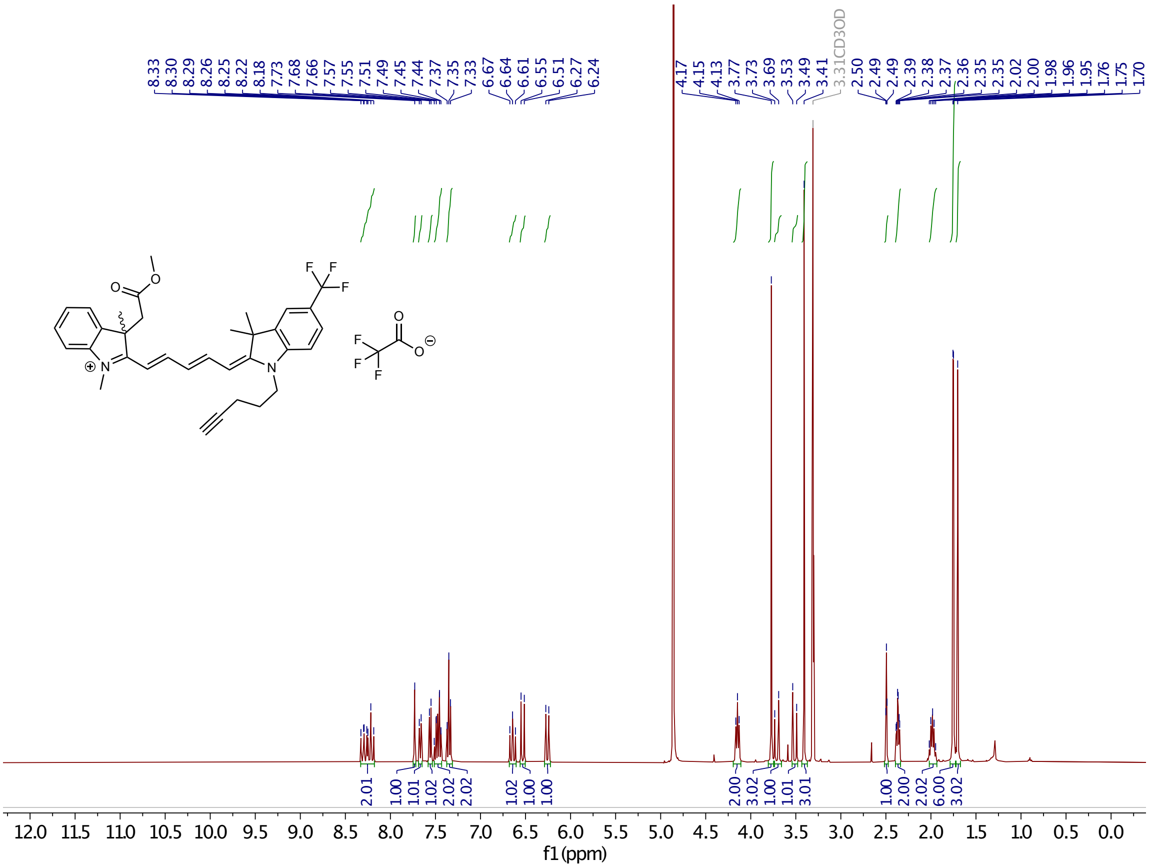

Figure 26. ^{1}H-NMR of **9d**.

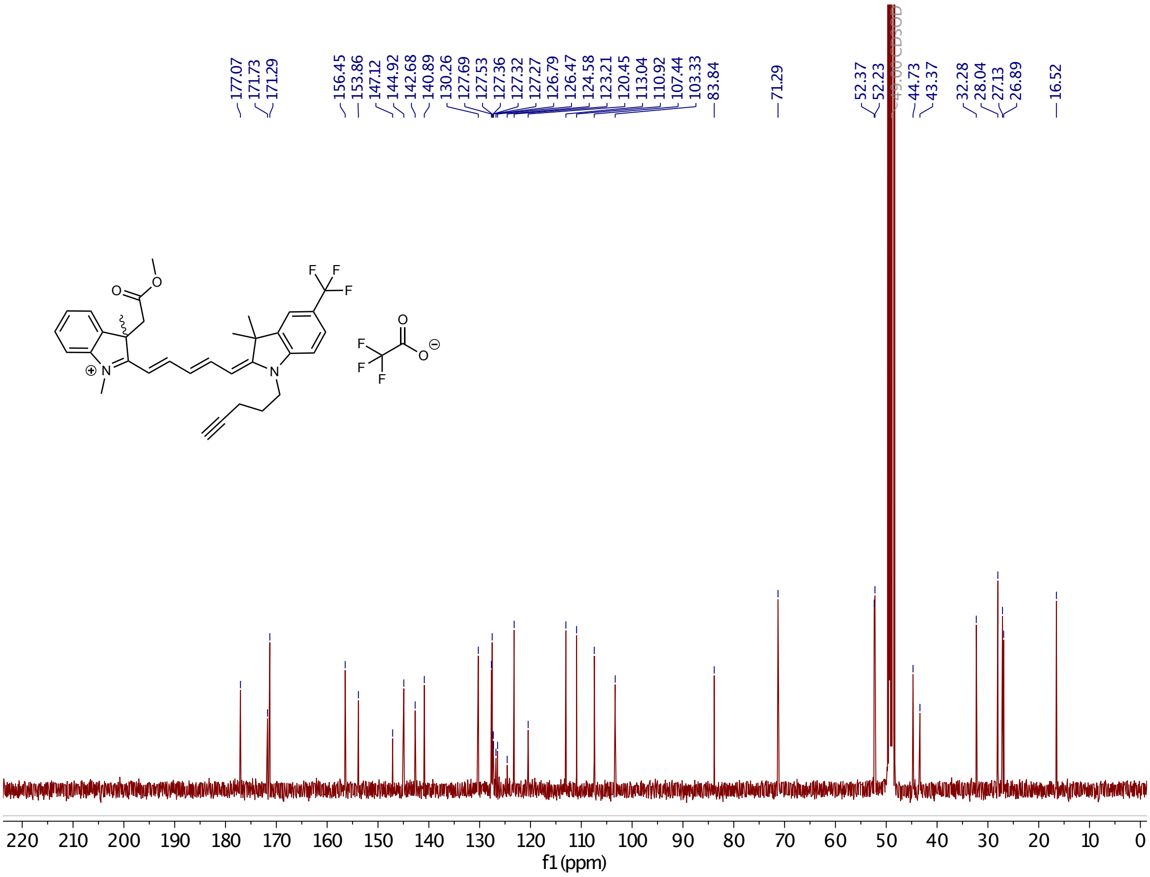

Figure 27. ^{13}C-NMR of **9d**.

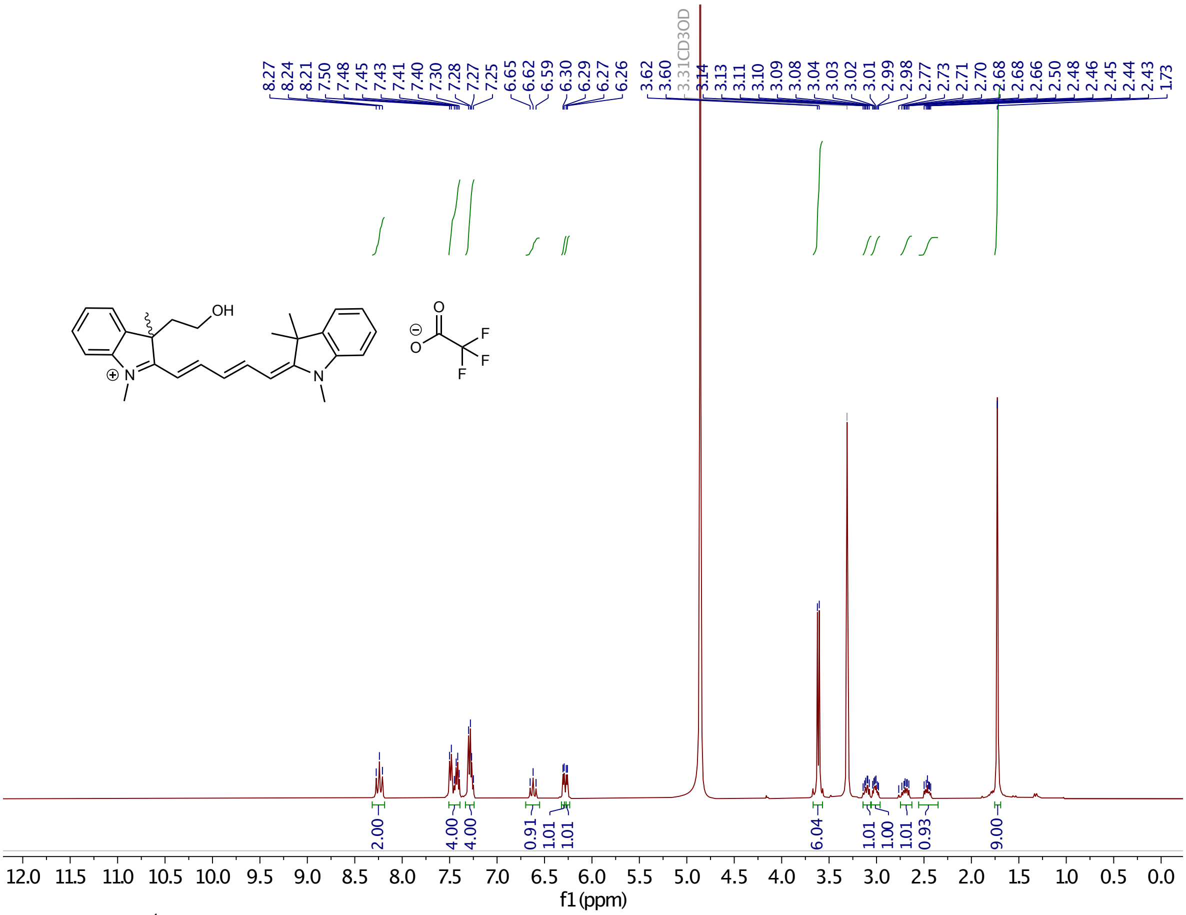

Figure 28. ^{1}H-NMR o **4a.**

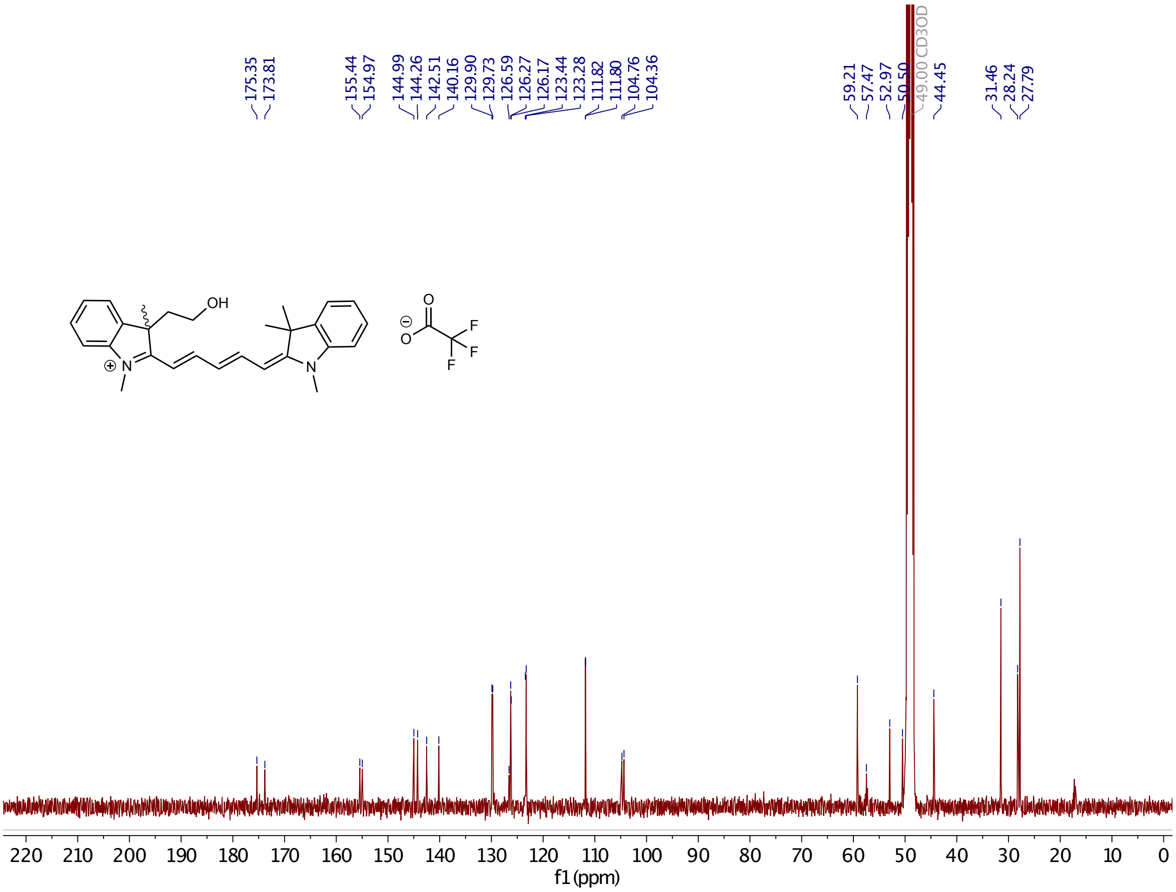

Figure 29. ^{13}C-NMR of **4a**.

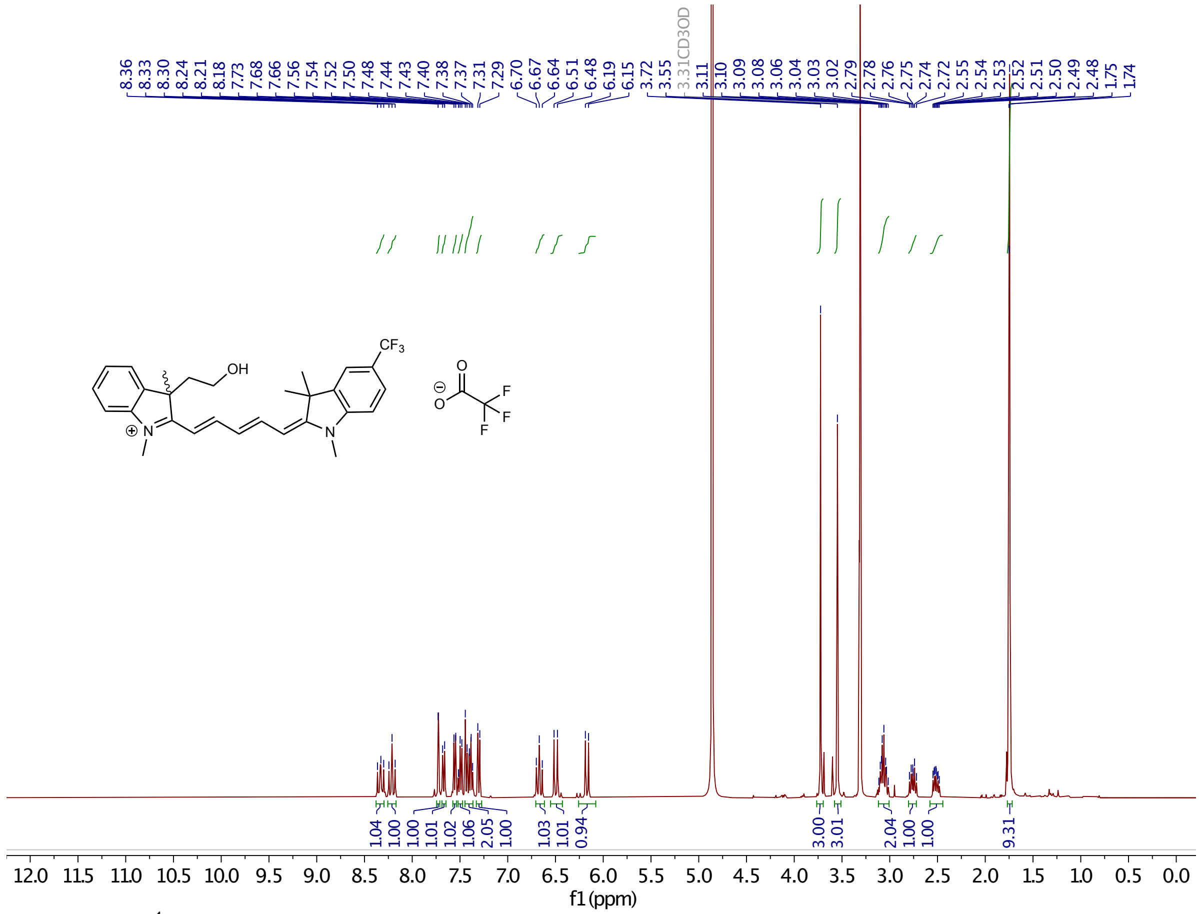

Figure 30. ^{1}H-NMR of **4b.**

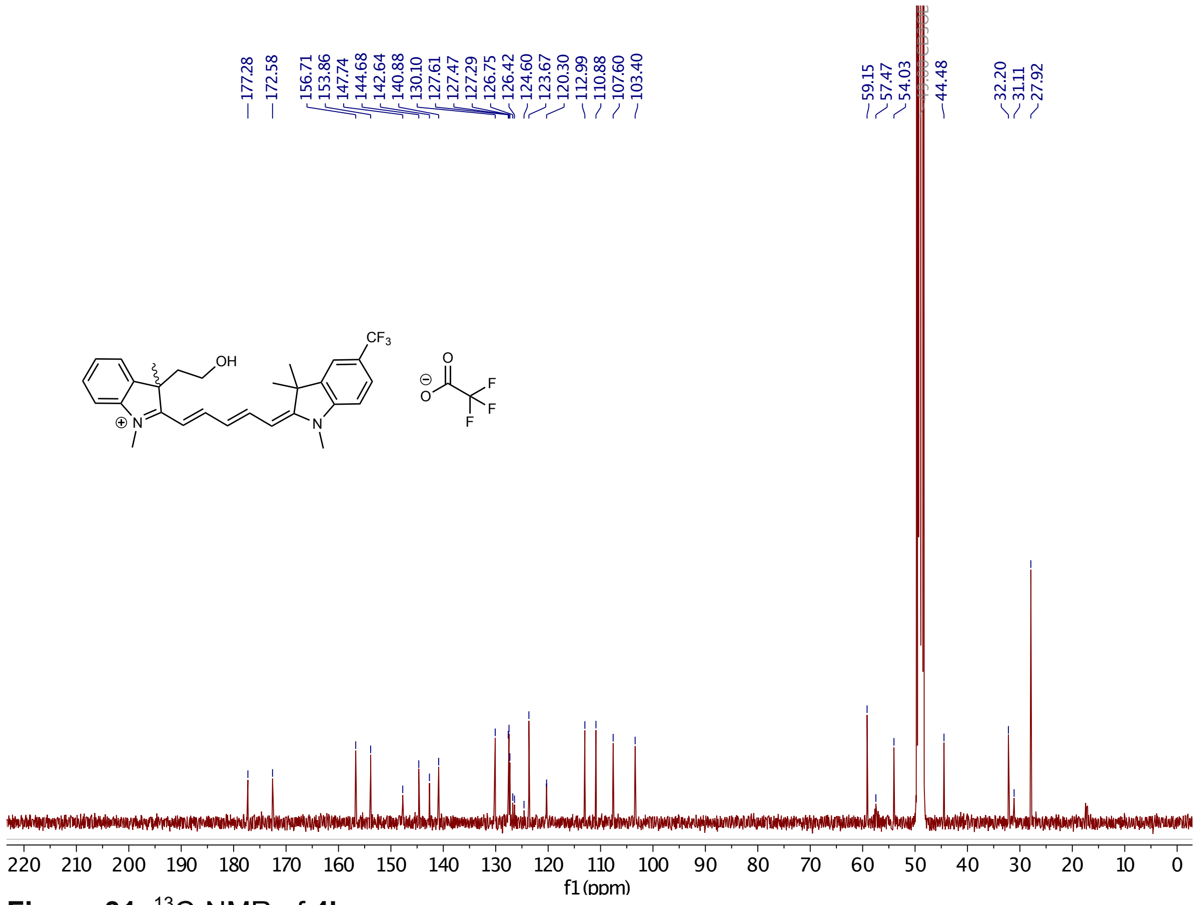

Figure 31. ^{13}C-NMR of **4b**.

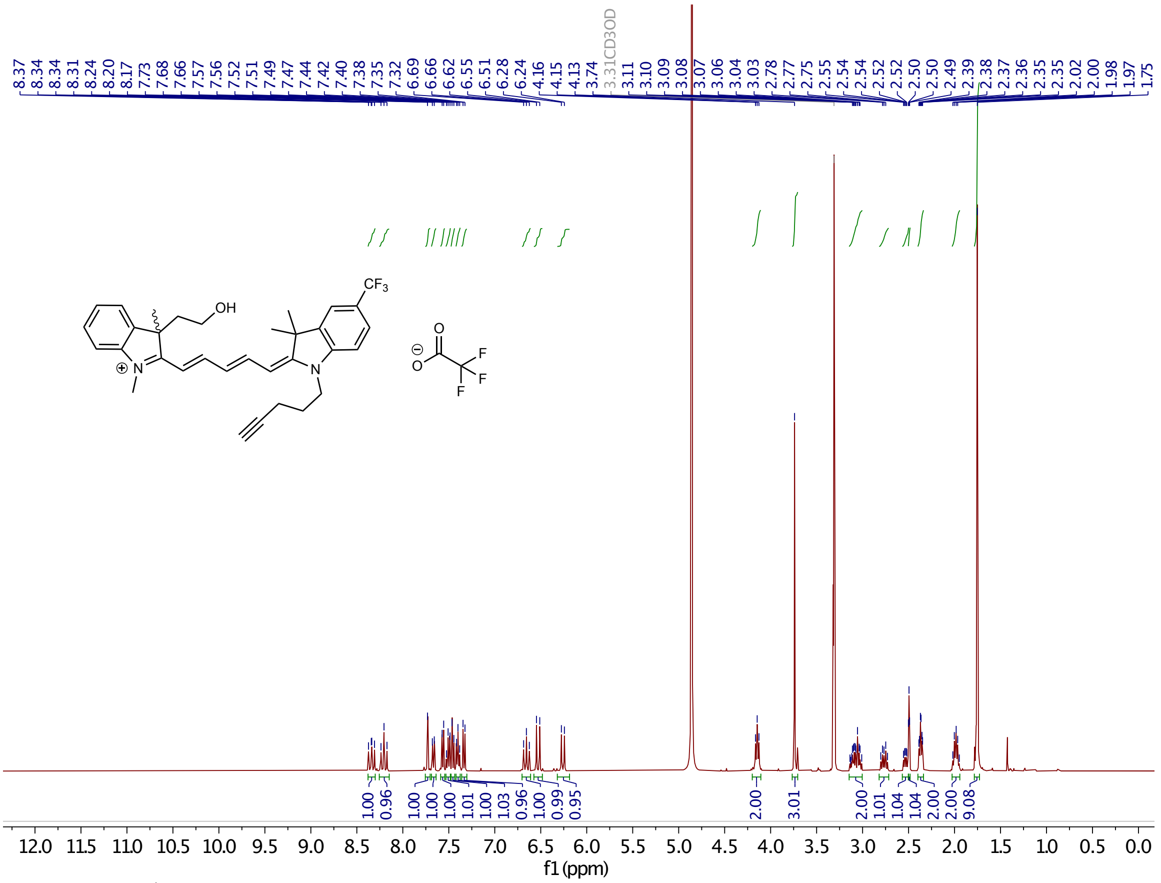

Figure 32. ^{1}H-NMR of **4c.**

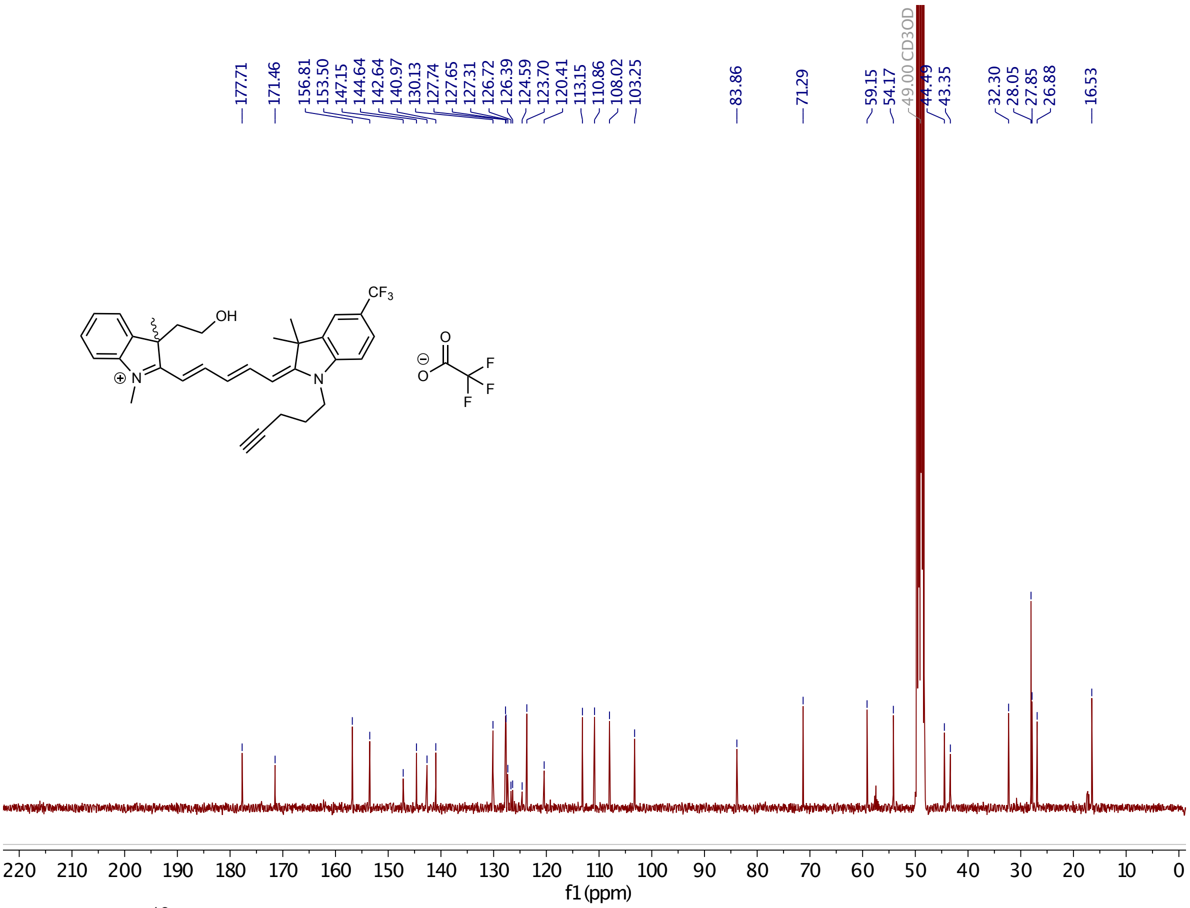

Figure 33. ^{13}C-NMR of **4c**.

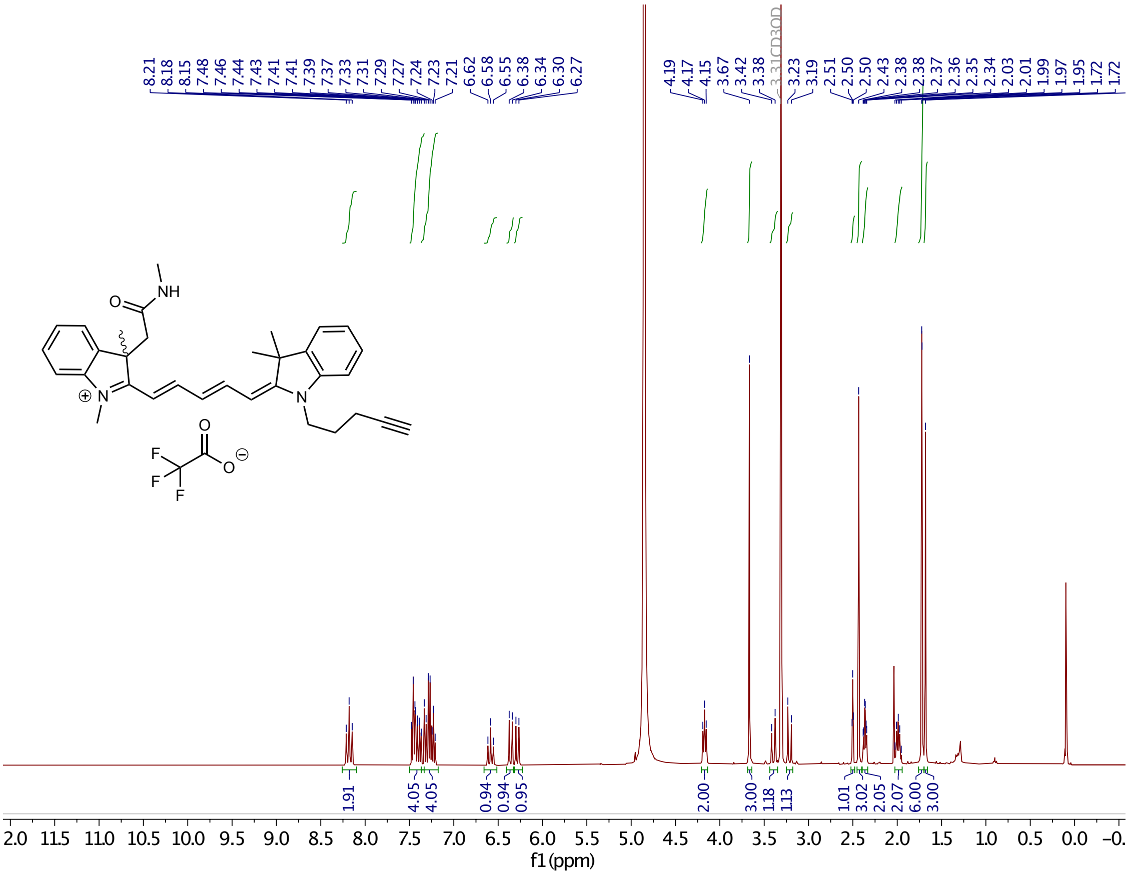

Figure 34. ^{1}H-NMR of **12**.

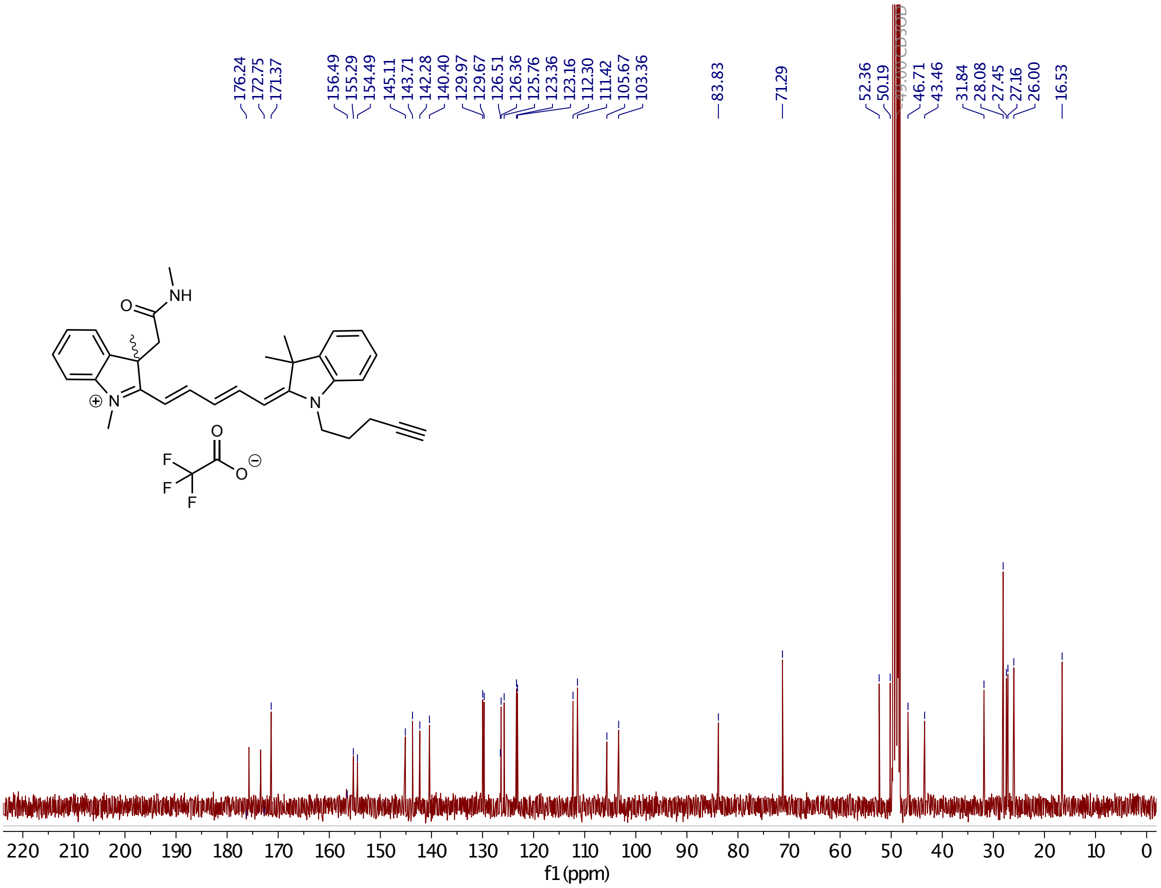

Figure 35. ^{13}C-NMR of **12**.

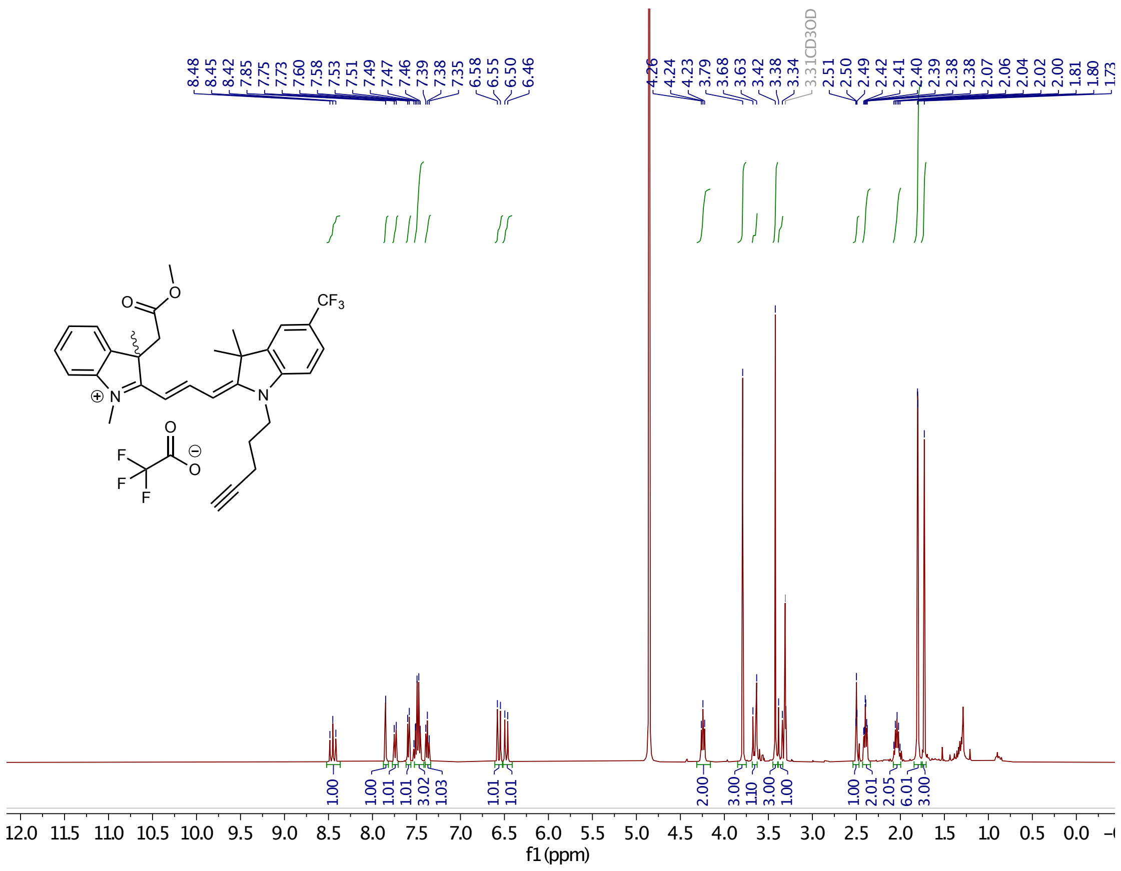

Figure 36. ^{1}H-NMR of **21**.

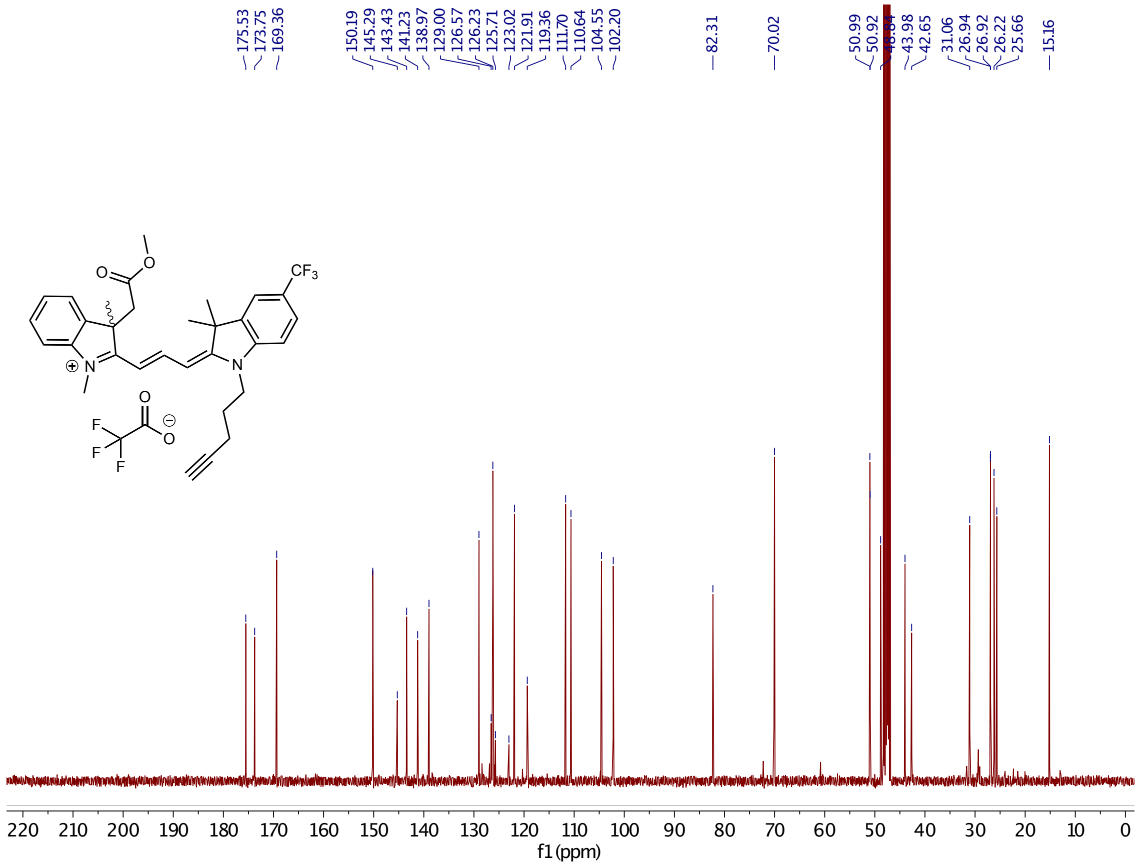

Figure 37. ^{13}C-NMR of **21**.

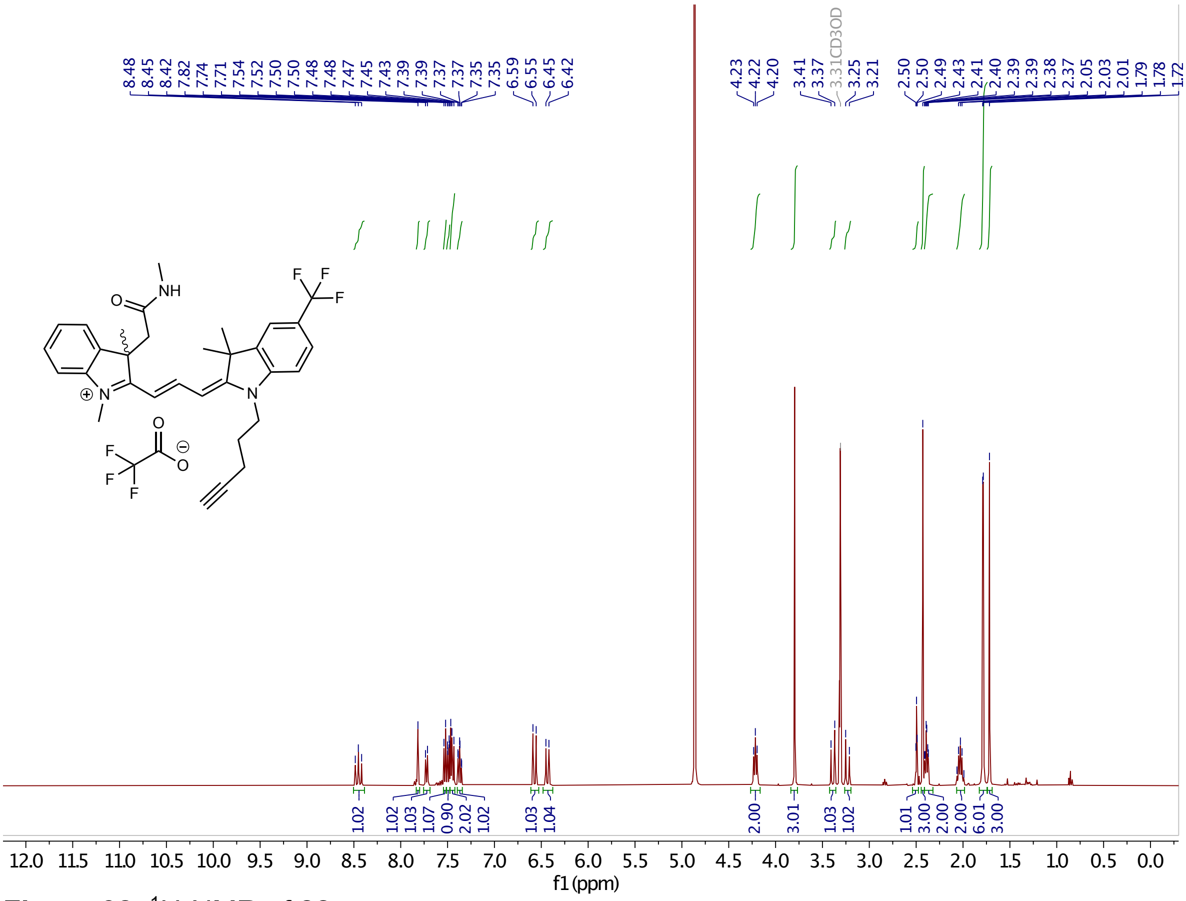

Figure 38. ^{1}H-NMR of **22**.

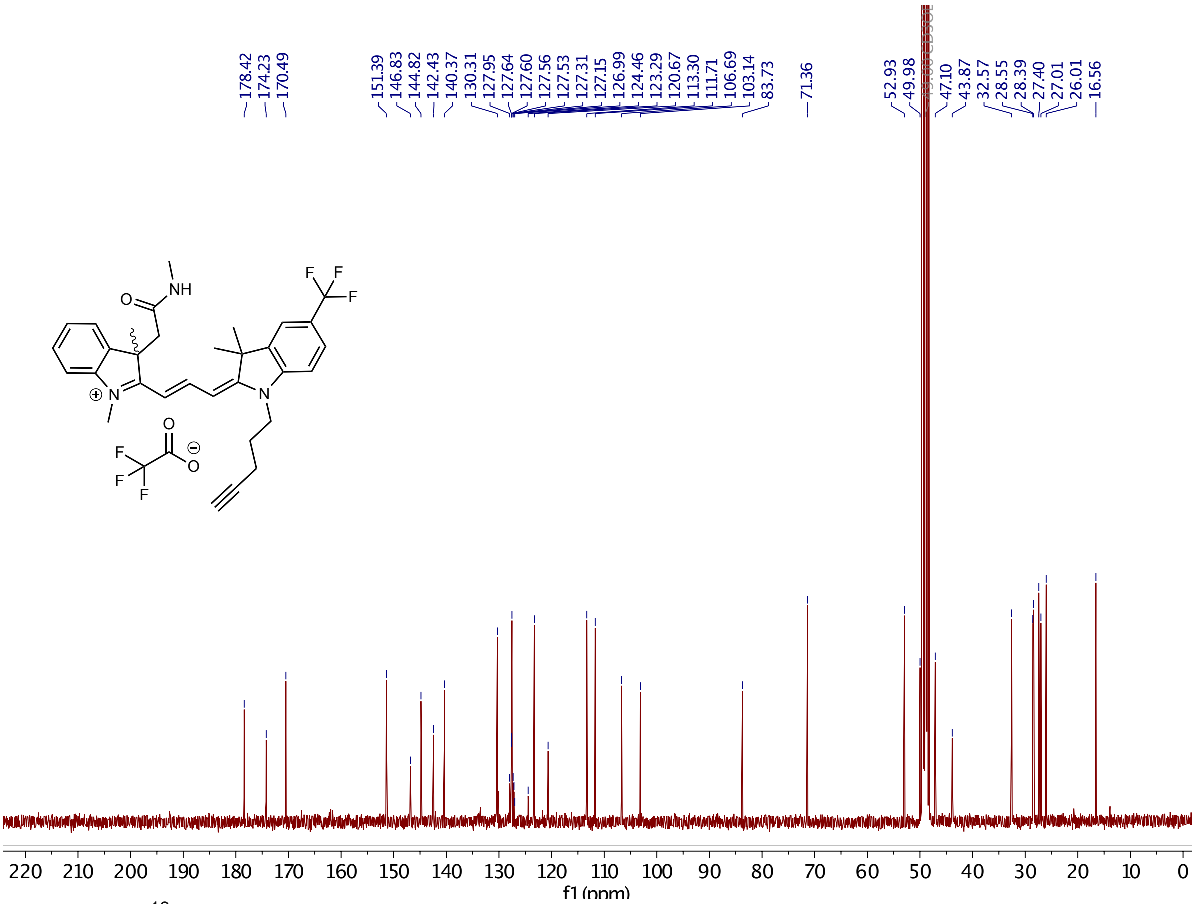

Figure 39. ^{13}C-NMR of **22**.

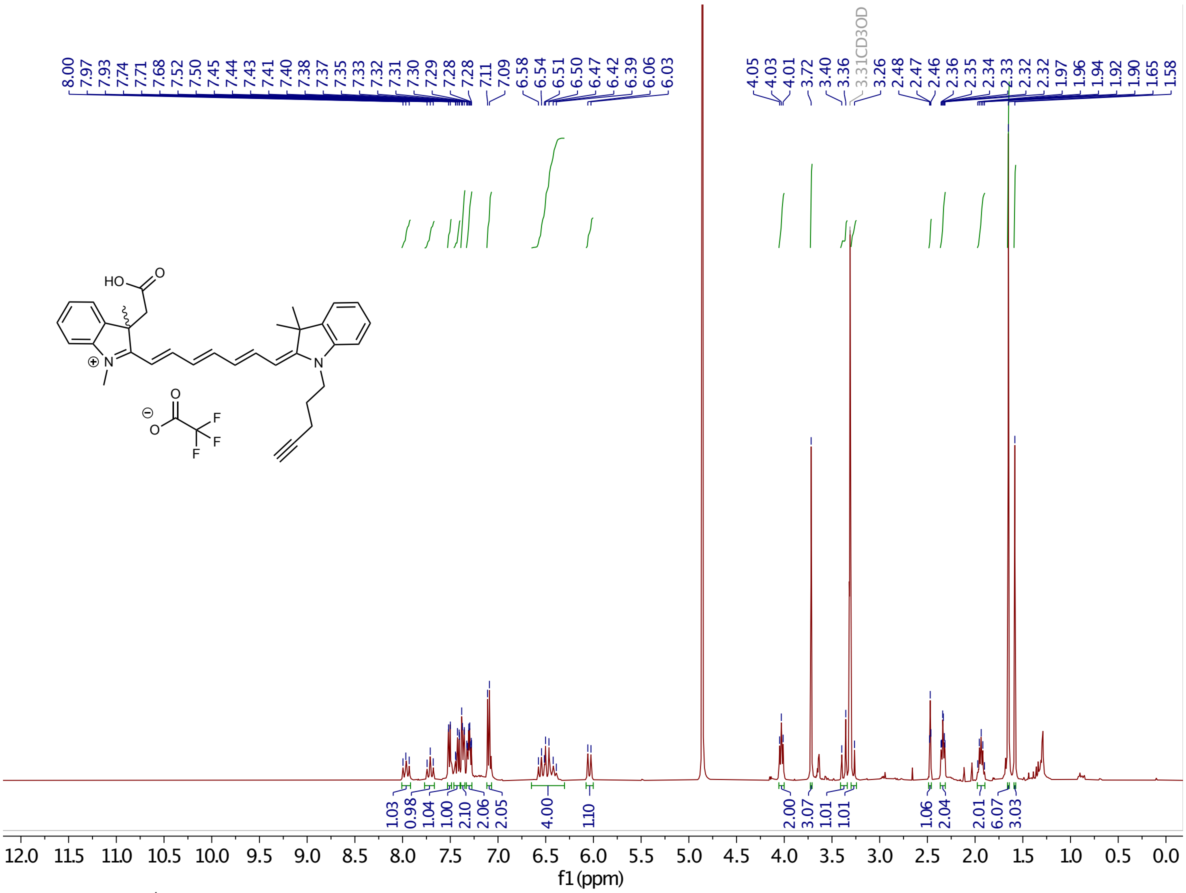

Figure 40. ¹H-NMR of **23**.

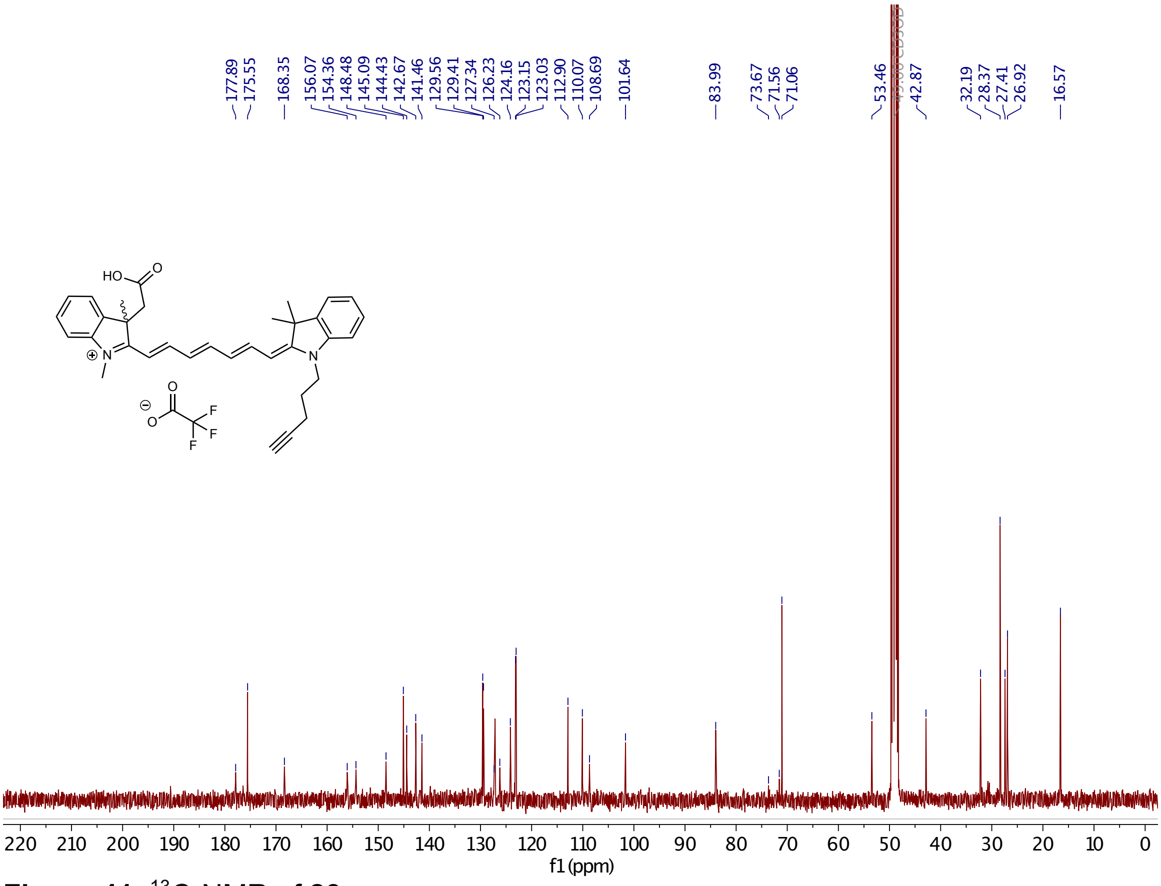

Figure 41. ¹³C-NMR of **23**.

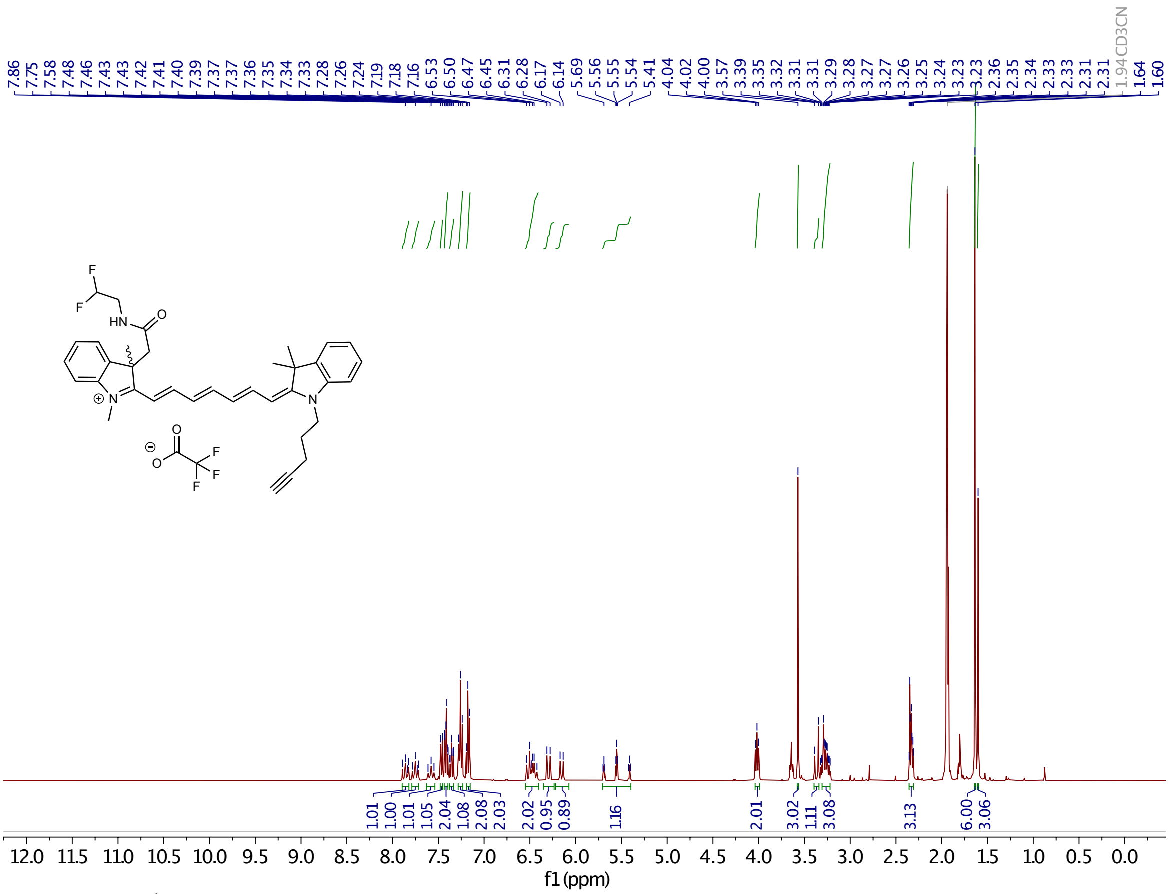

Figure 42. ^{1}H-NMR of **24**.

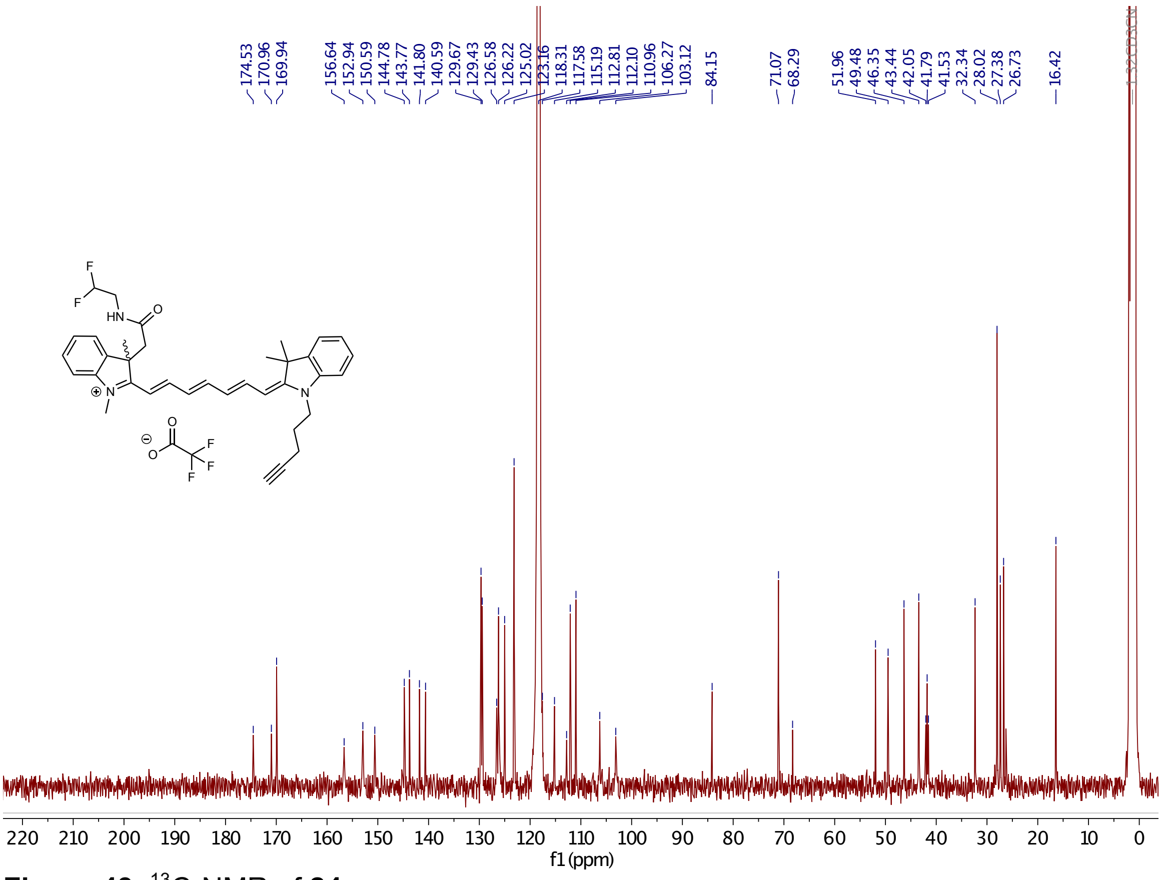

Figure 43. ^{13}C-NMR of **24**.

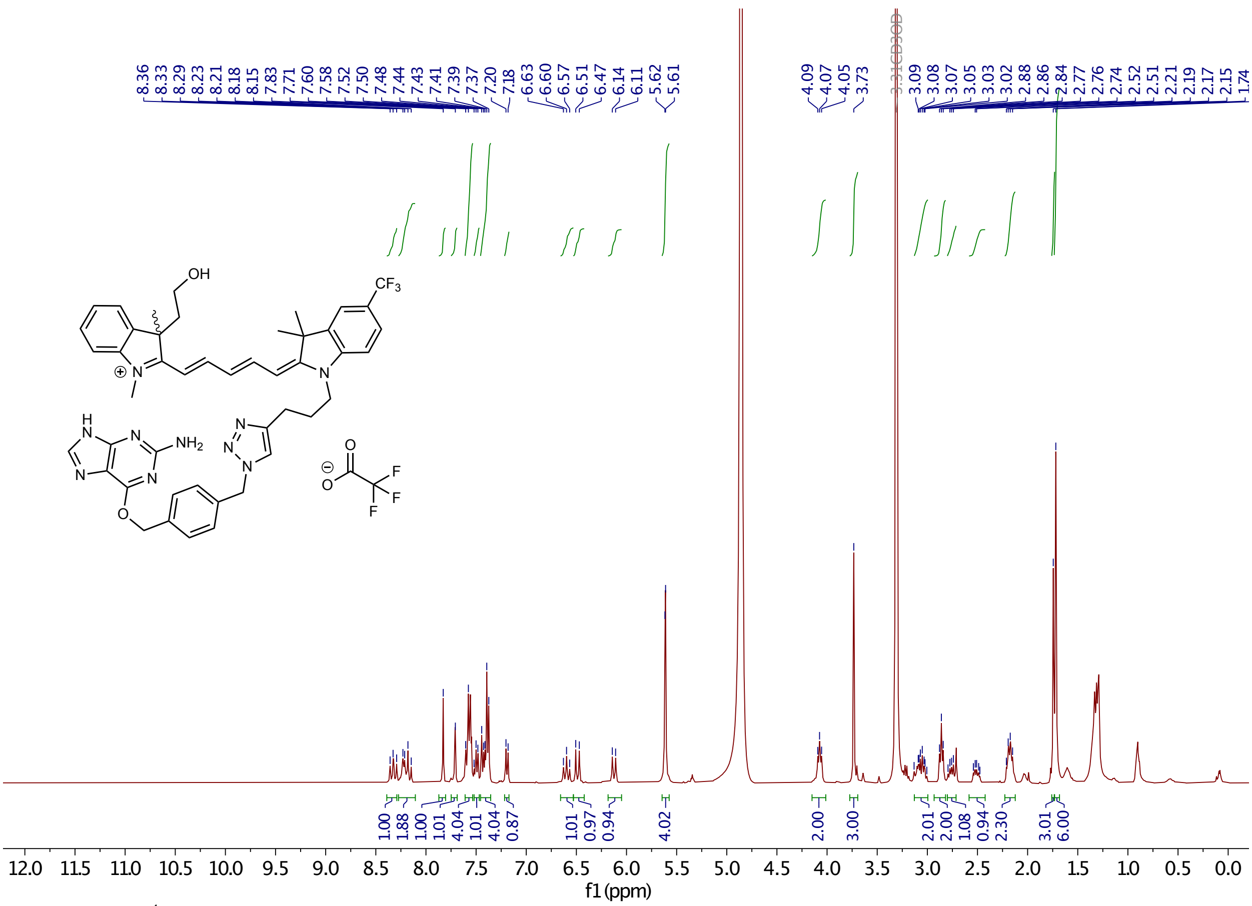

Figure 44. ^{1}H-NMR of **11**.

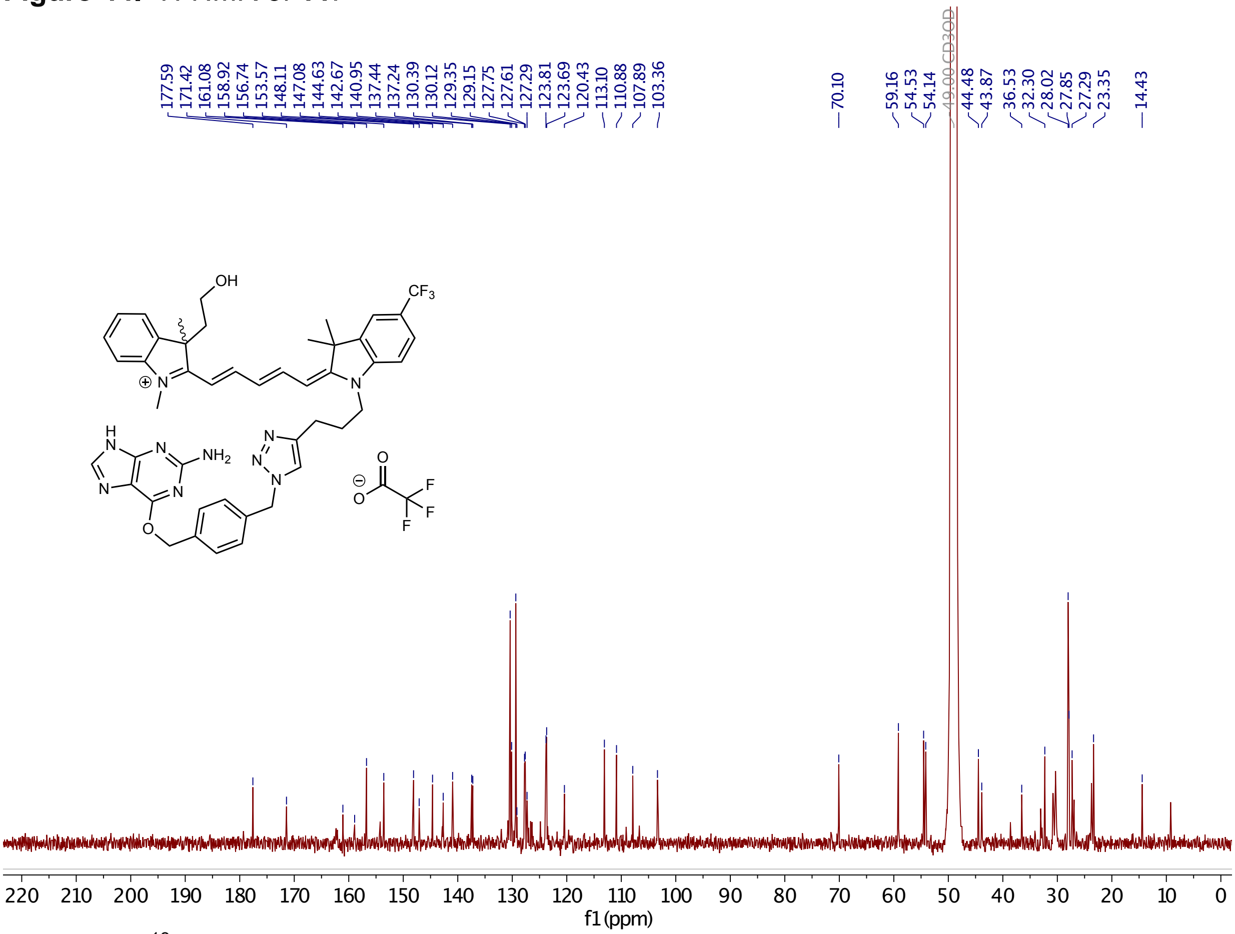

Figure 45. ^{13}C-NMR of **11**.

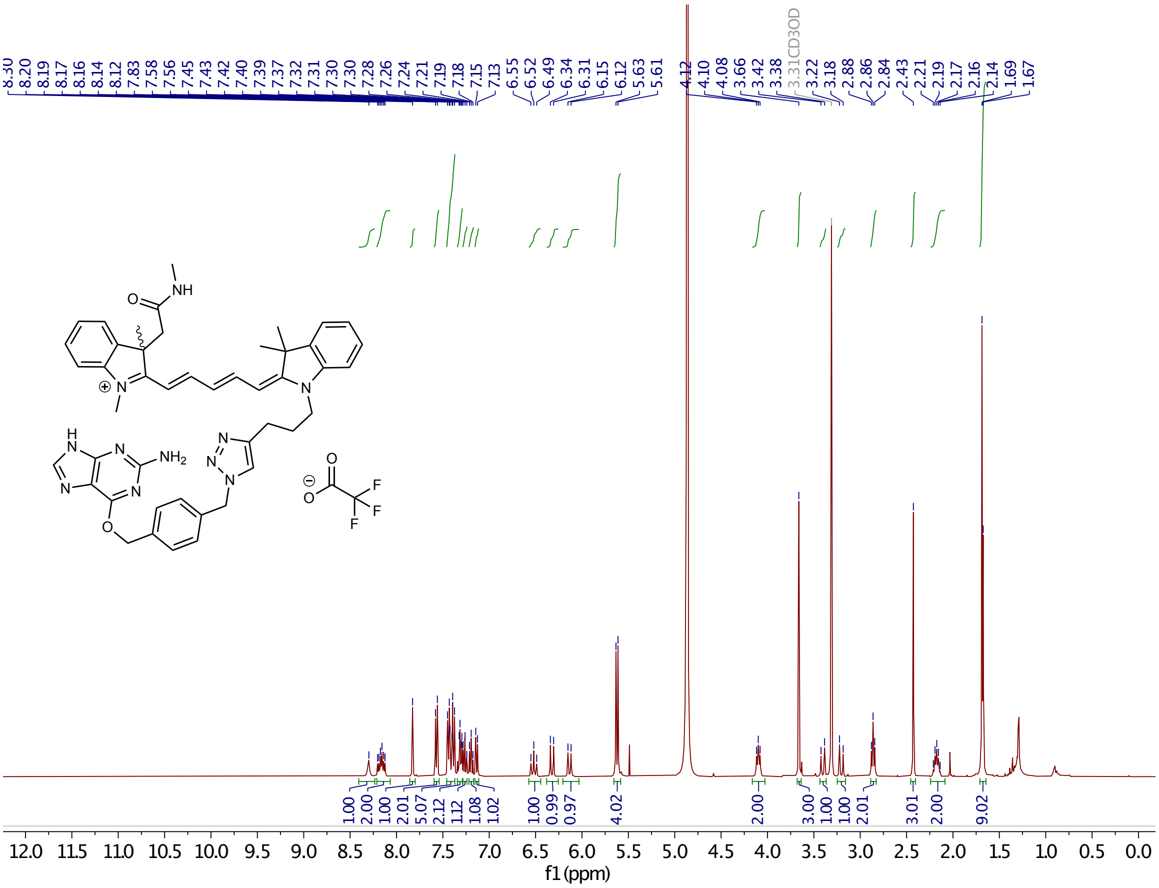

Figure 46. ^{1}H-NMR of **15**.

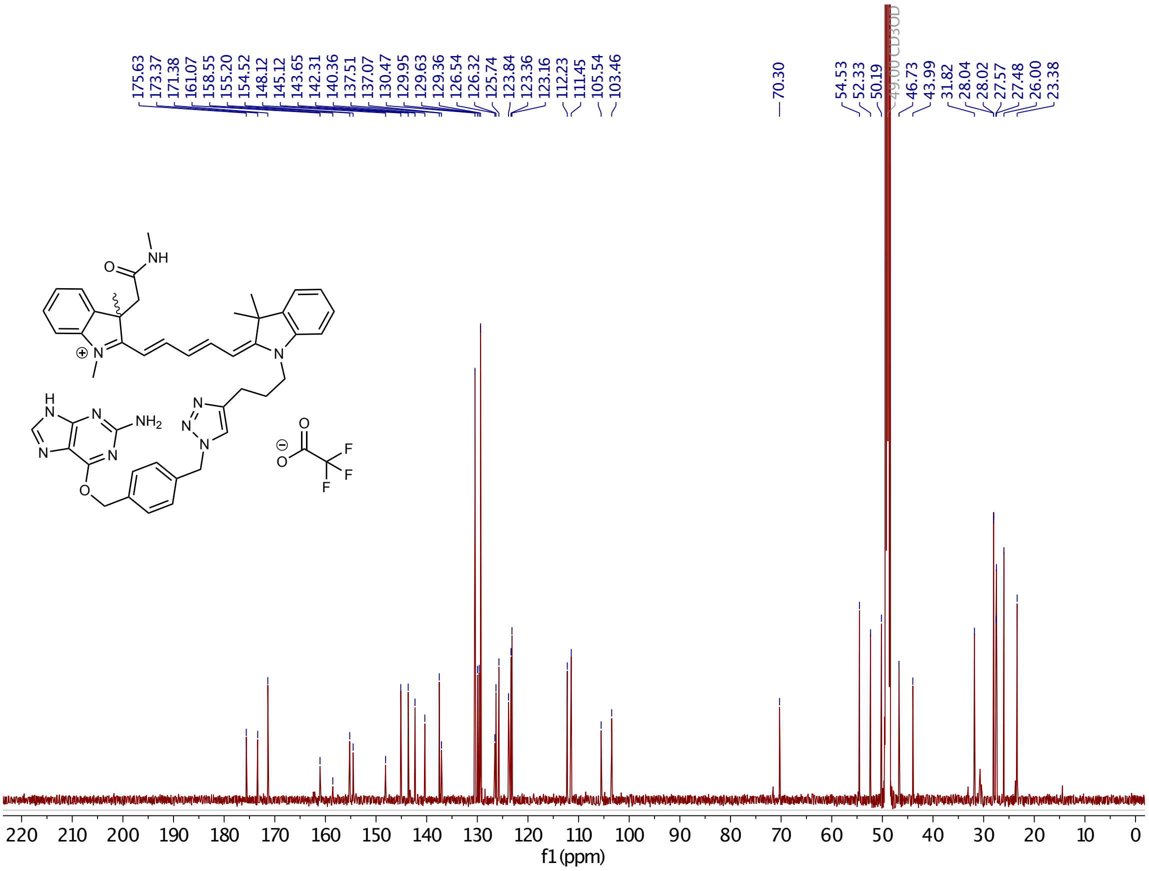

Figure 47. ^{13}C-NMR of **15**.

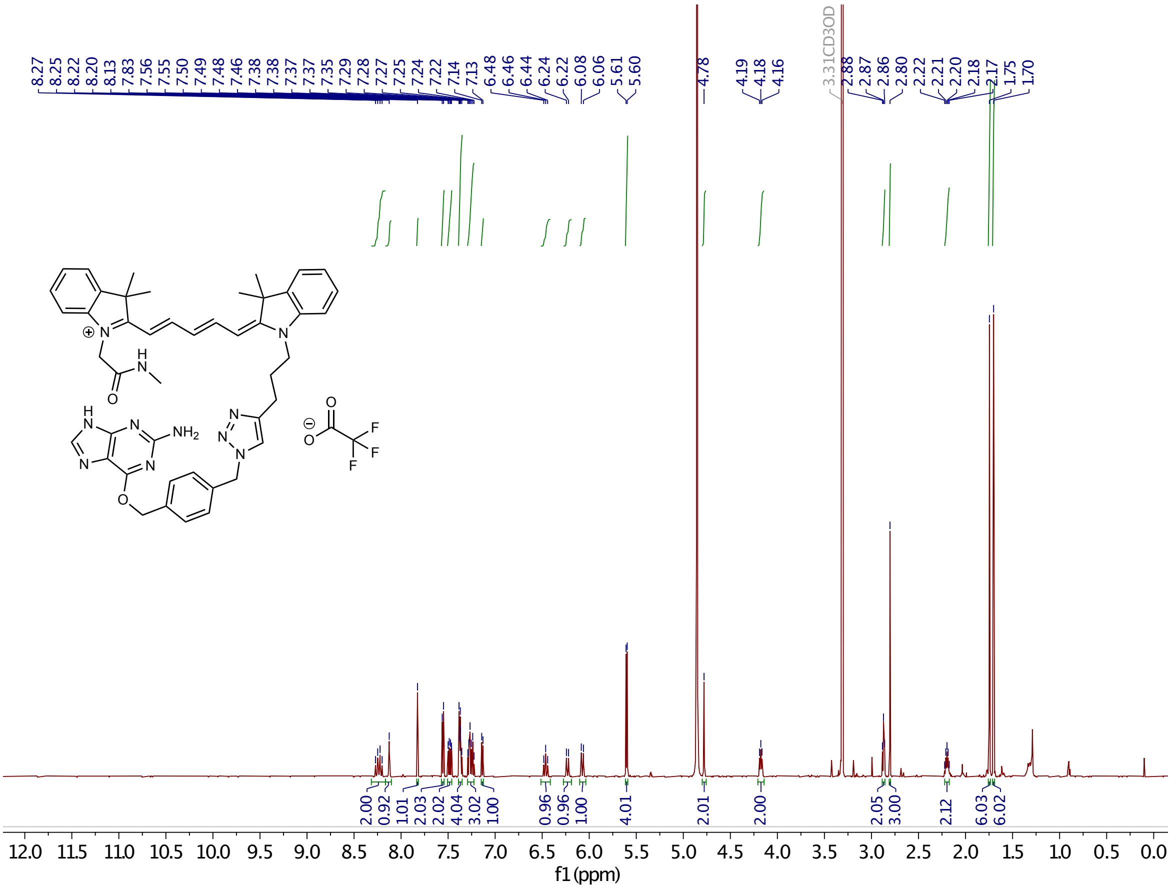

Figure 48. ^{1}H-NMR of **16**.

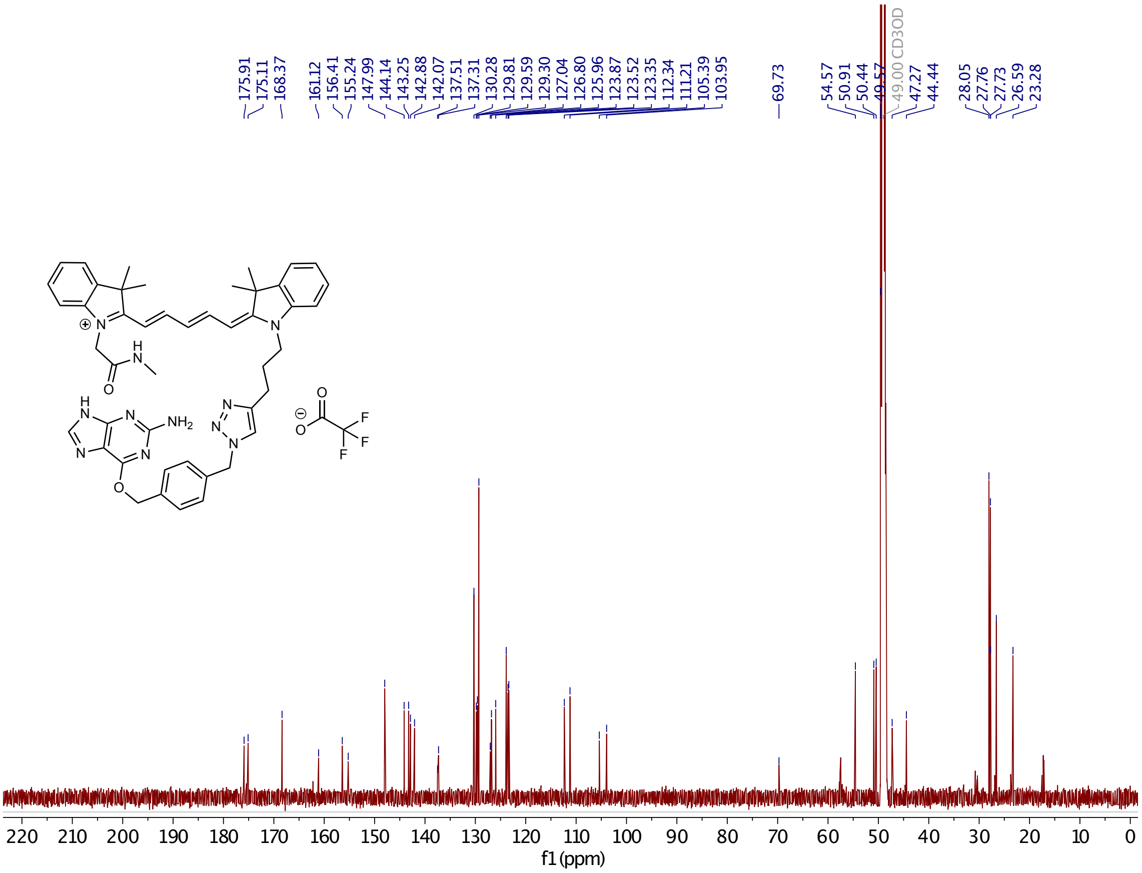

Figure 49. ^{13}C-NMR of **16**.

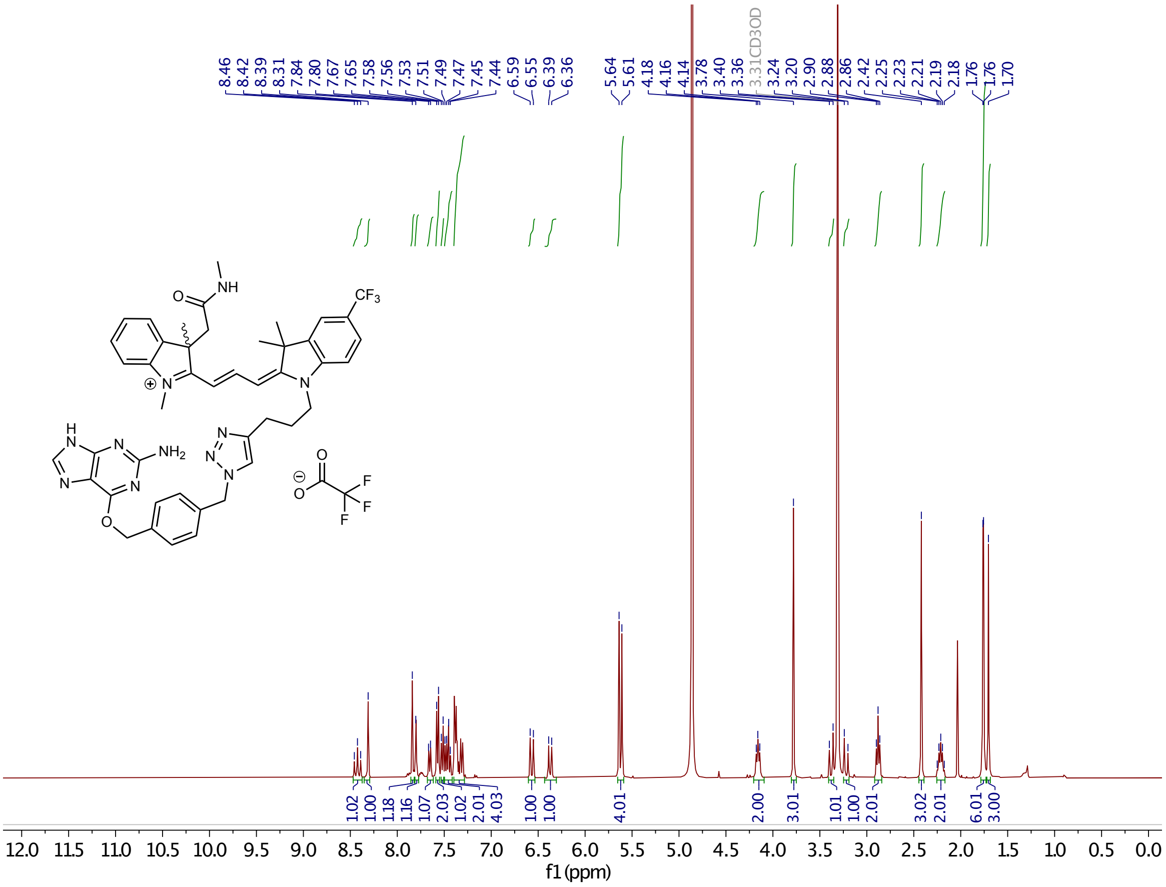

Figure 50. ^{1}H-NMR of **19**.

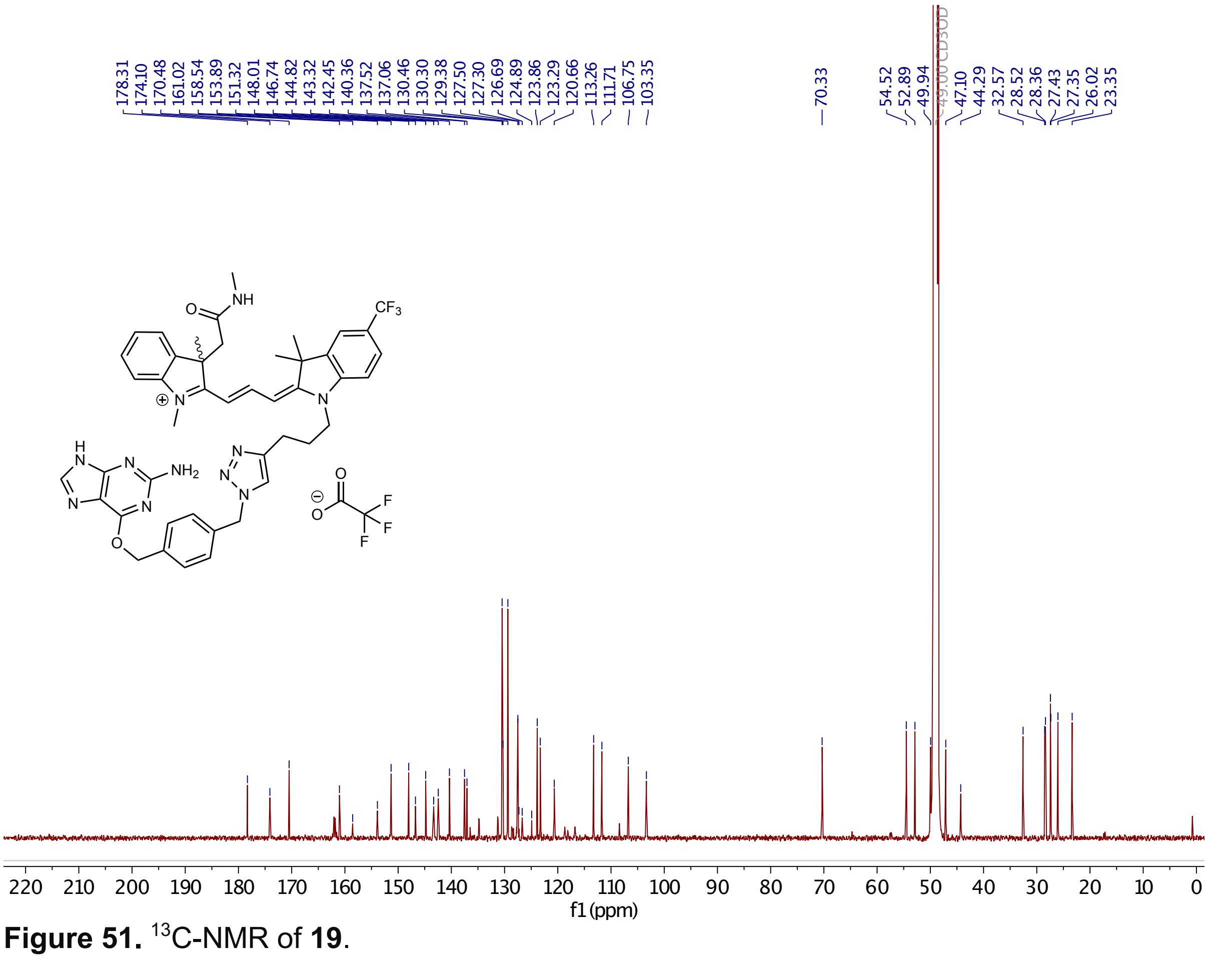

Figure 51. ^{13}C-NMR of **19**.

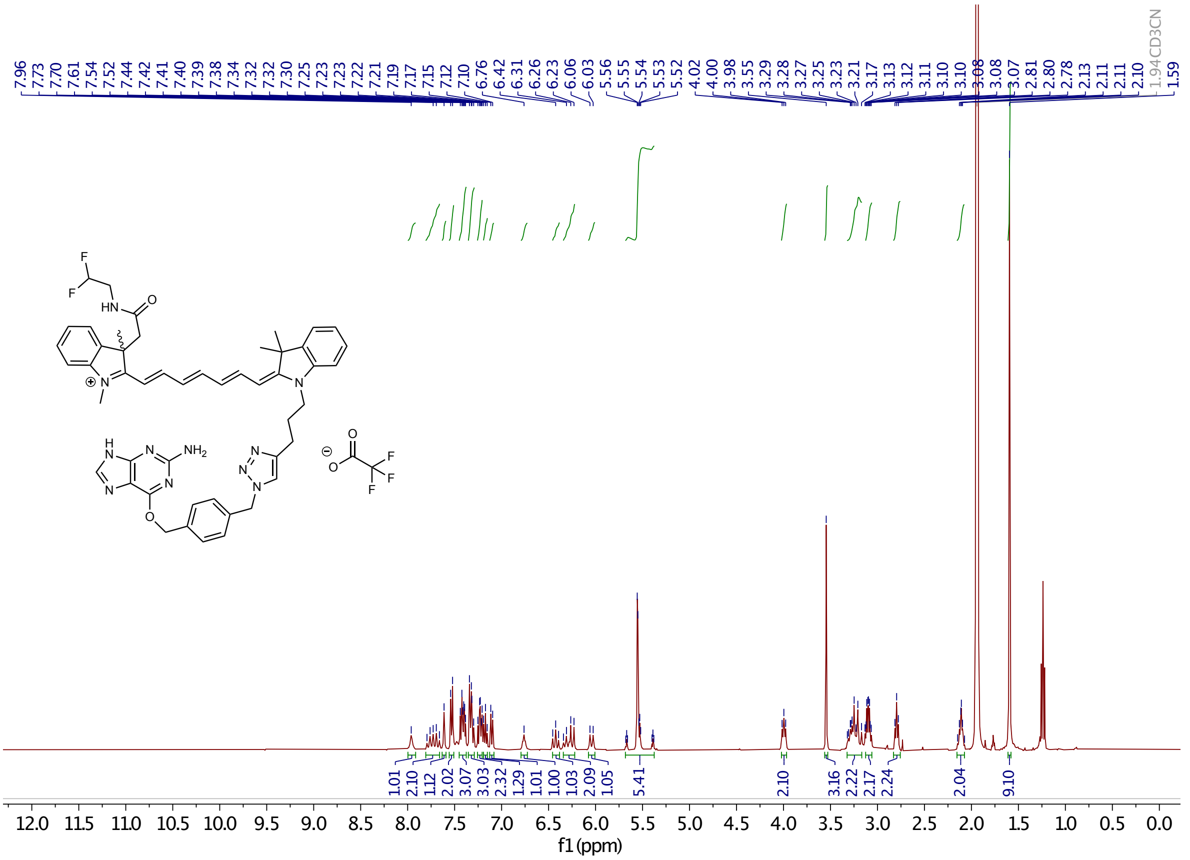

Figure 52. ^{1}H-NMR of **20**.

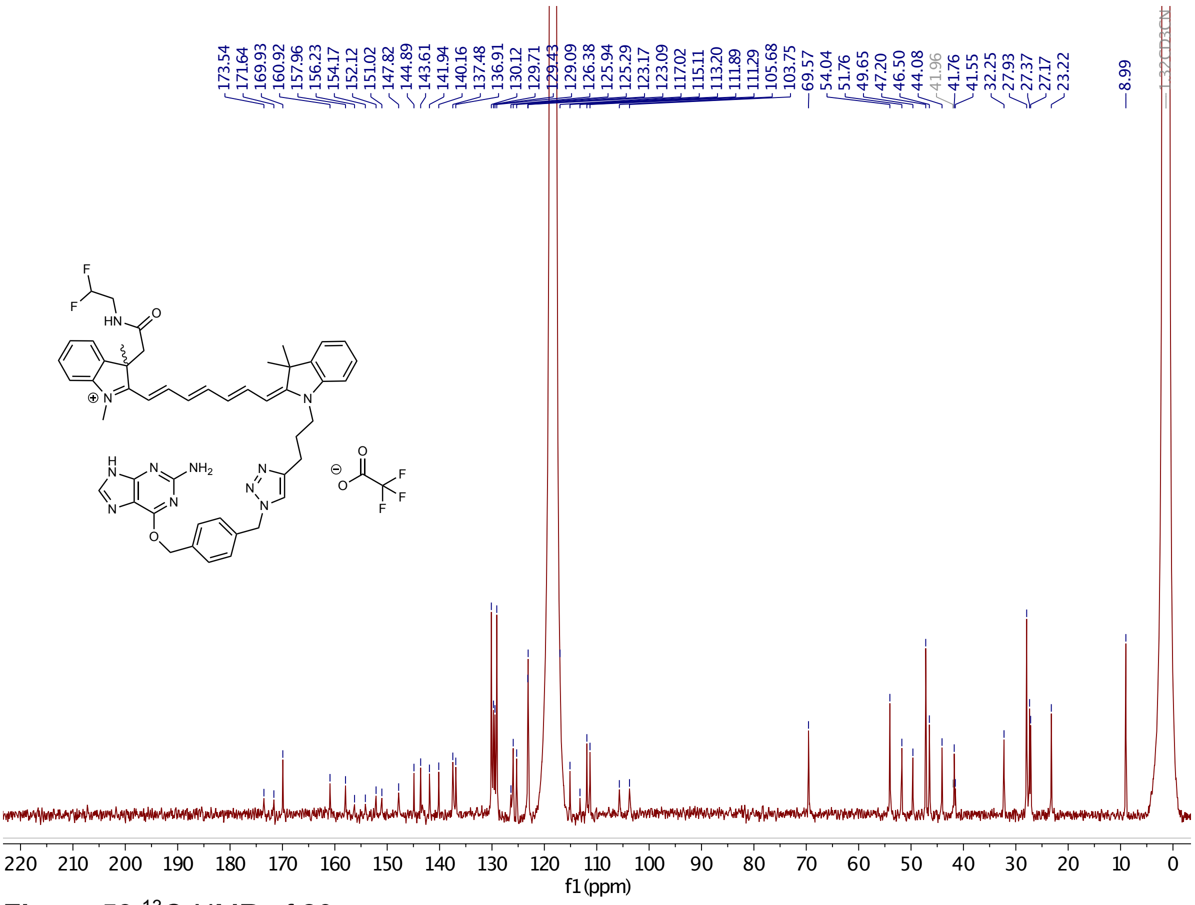

Figure 53 ^{13}C-NMR of **20**.

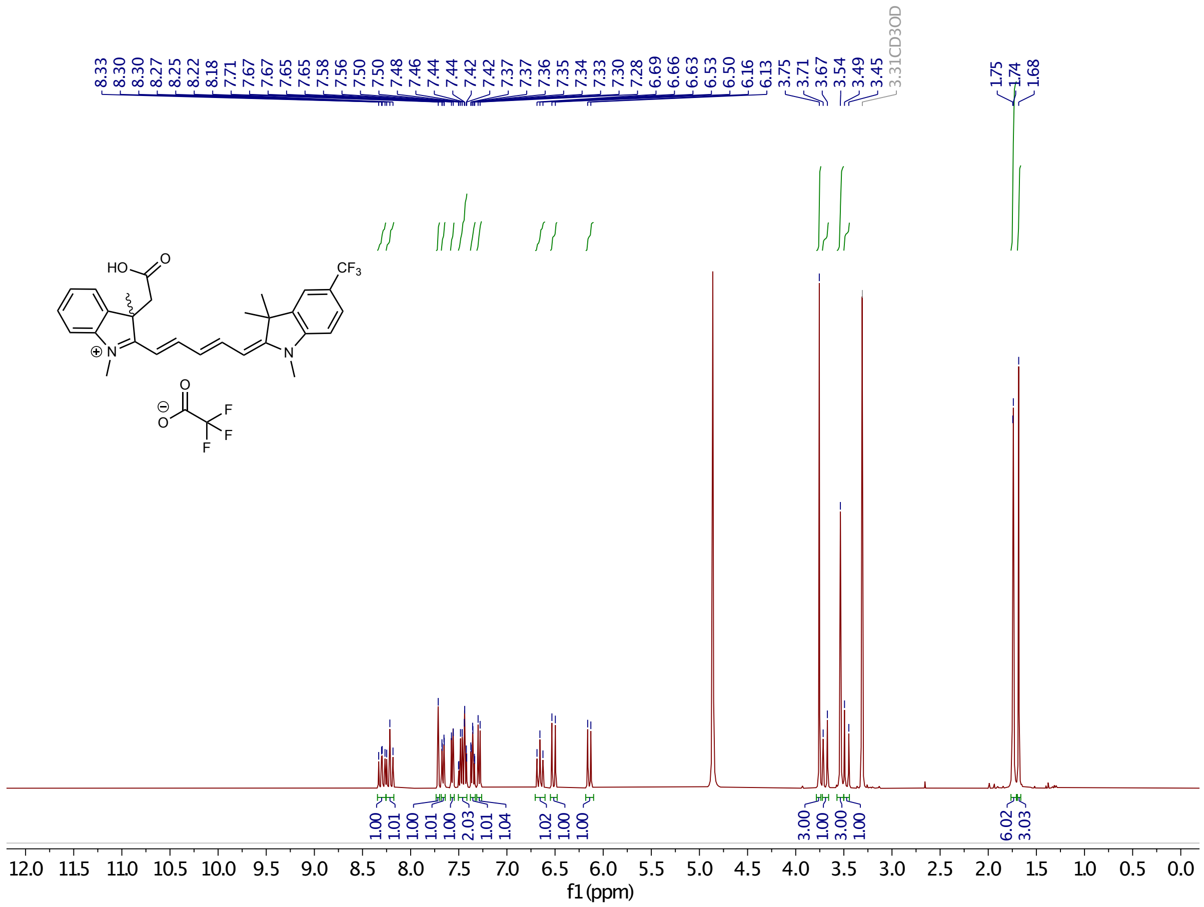

Figure 54. ^{1}H-NMR of **S1**.

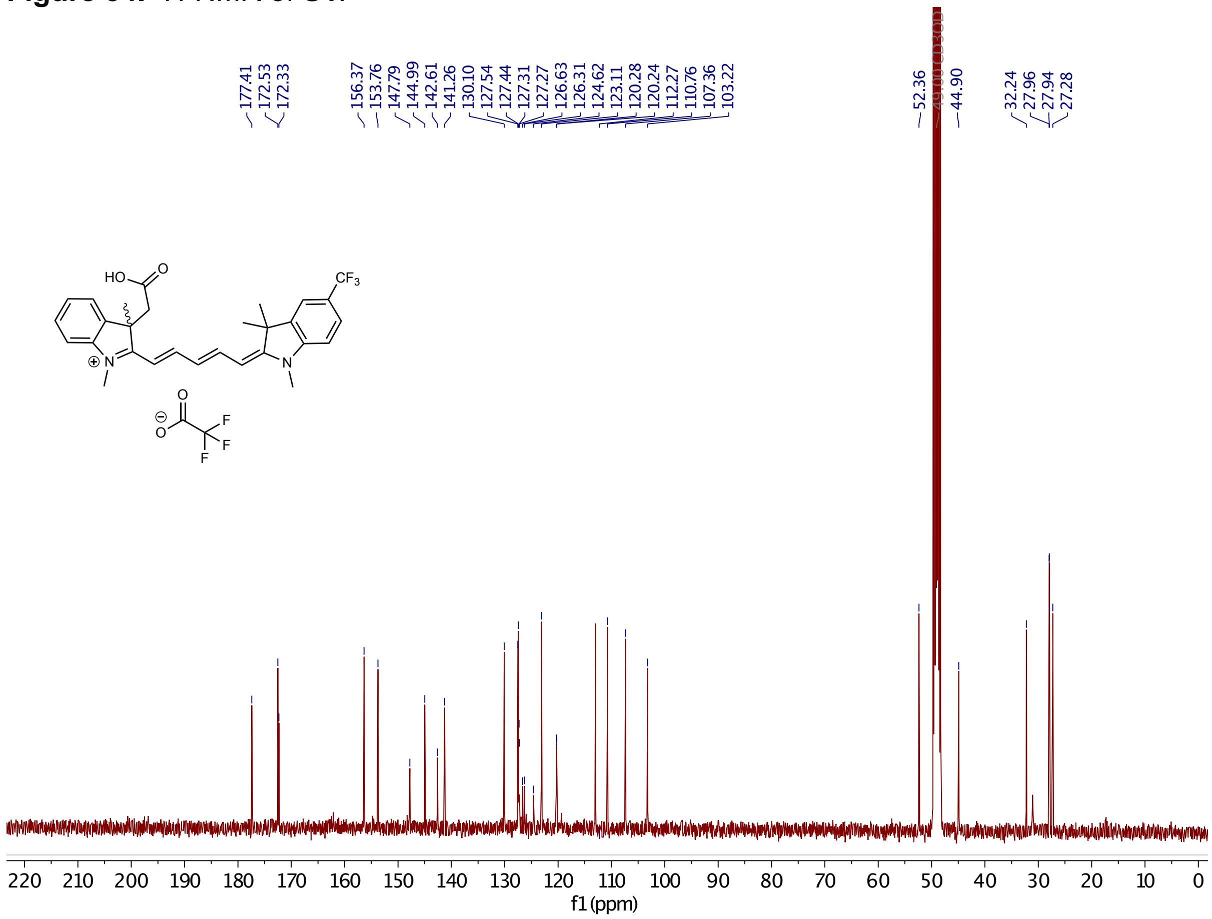

Figure 55. ^{13}C-NMR of **S1**.

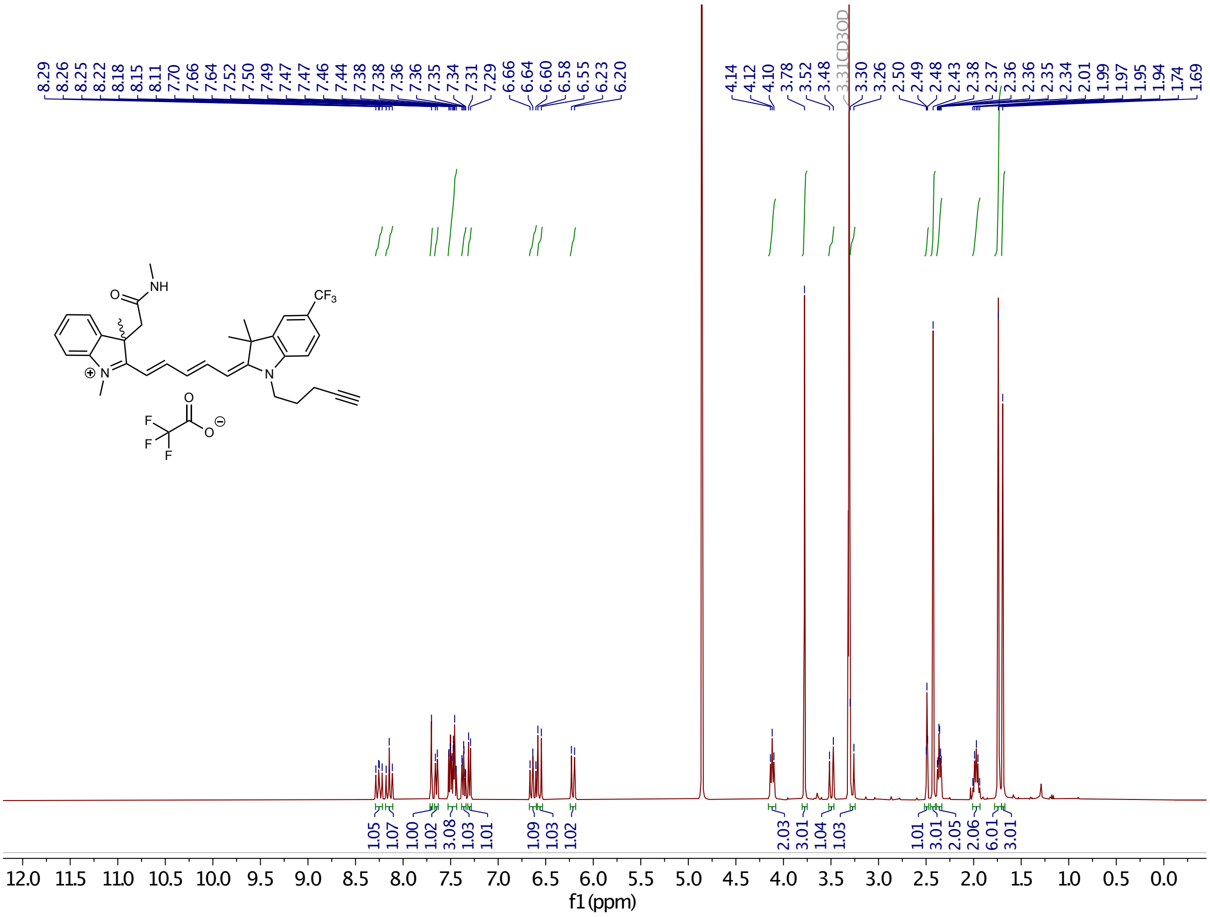

Figure 56. ^{1}H-NMR of **S2**.

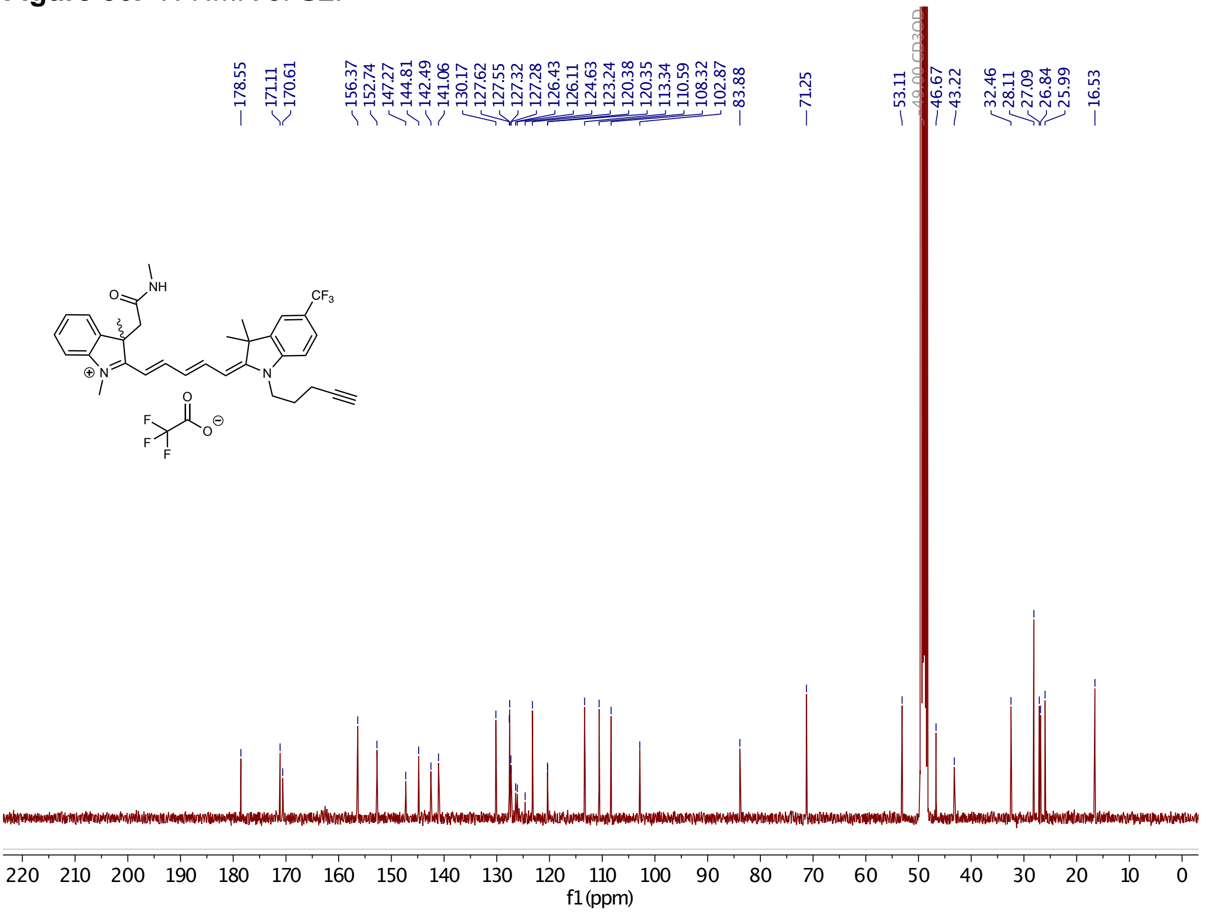

Figure 57 ^{13}C-NMR of **S2**

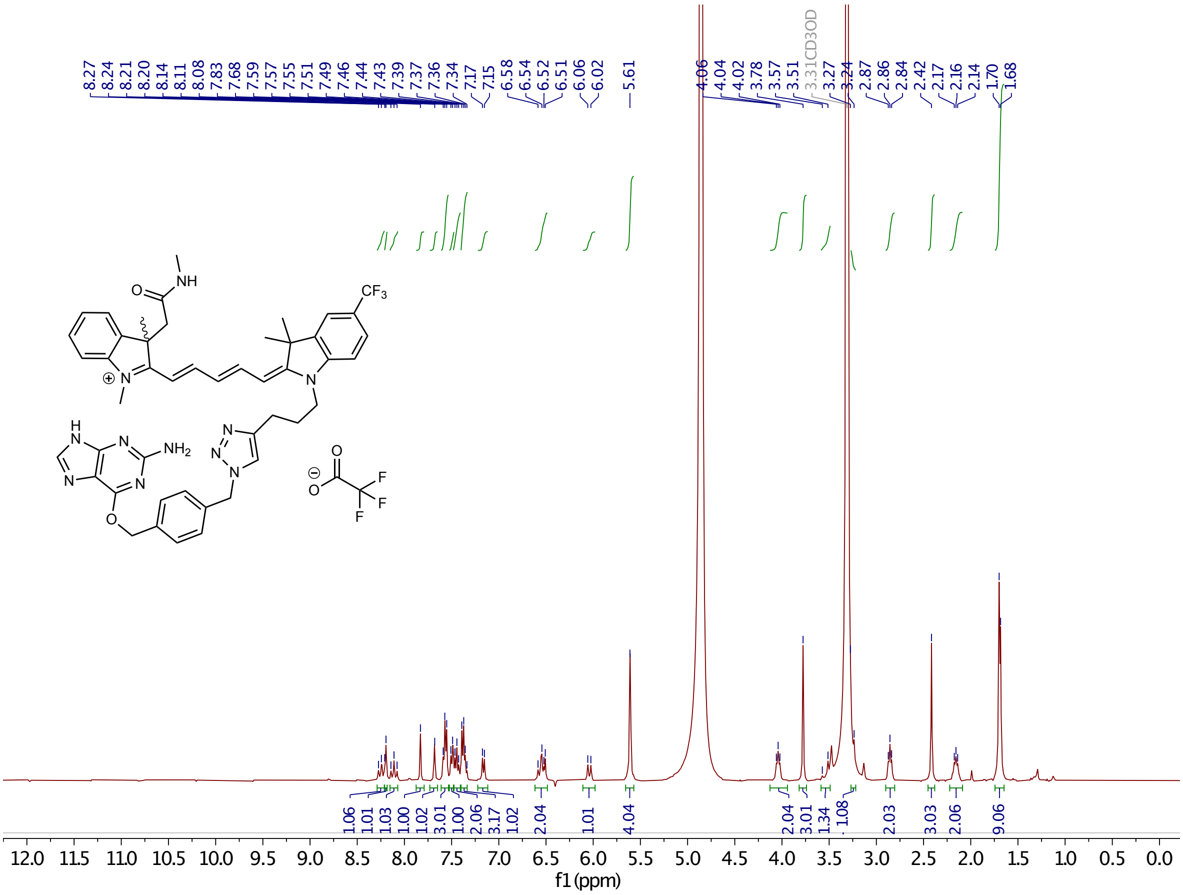

Figure 58. ^{1}H-NMR of **S3**.

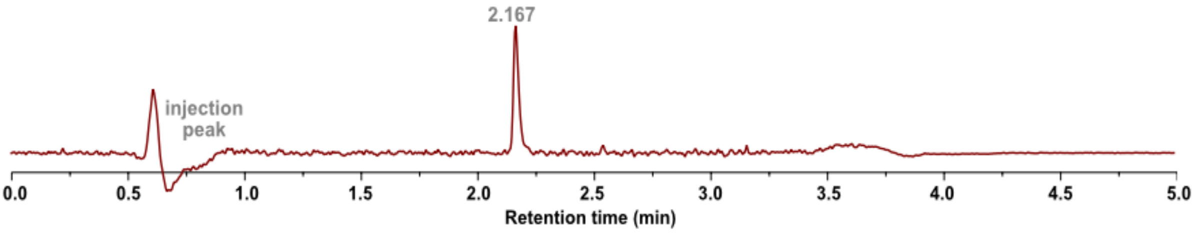

Figure 59. Integrated absorbance signal (190-300 nm) of analytical LC-MS run (10 → 95% CH_3CN in ddH_2O + 0.02% TFA + 0.04% FA over 3 min, then additional 2 min at 95%) of **S3**.

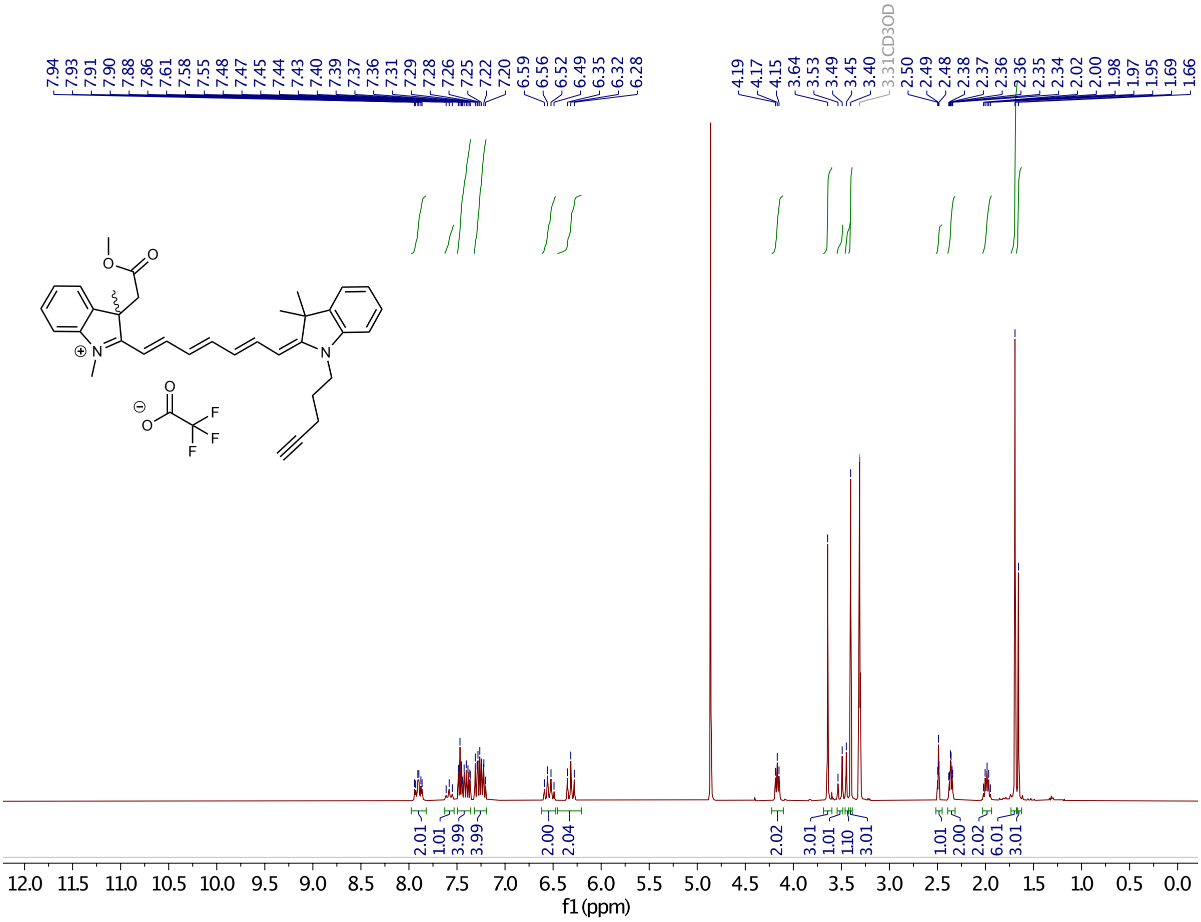

Figure 60. ^{1}H-NMR of **S4**.

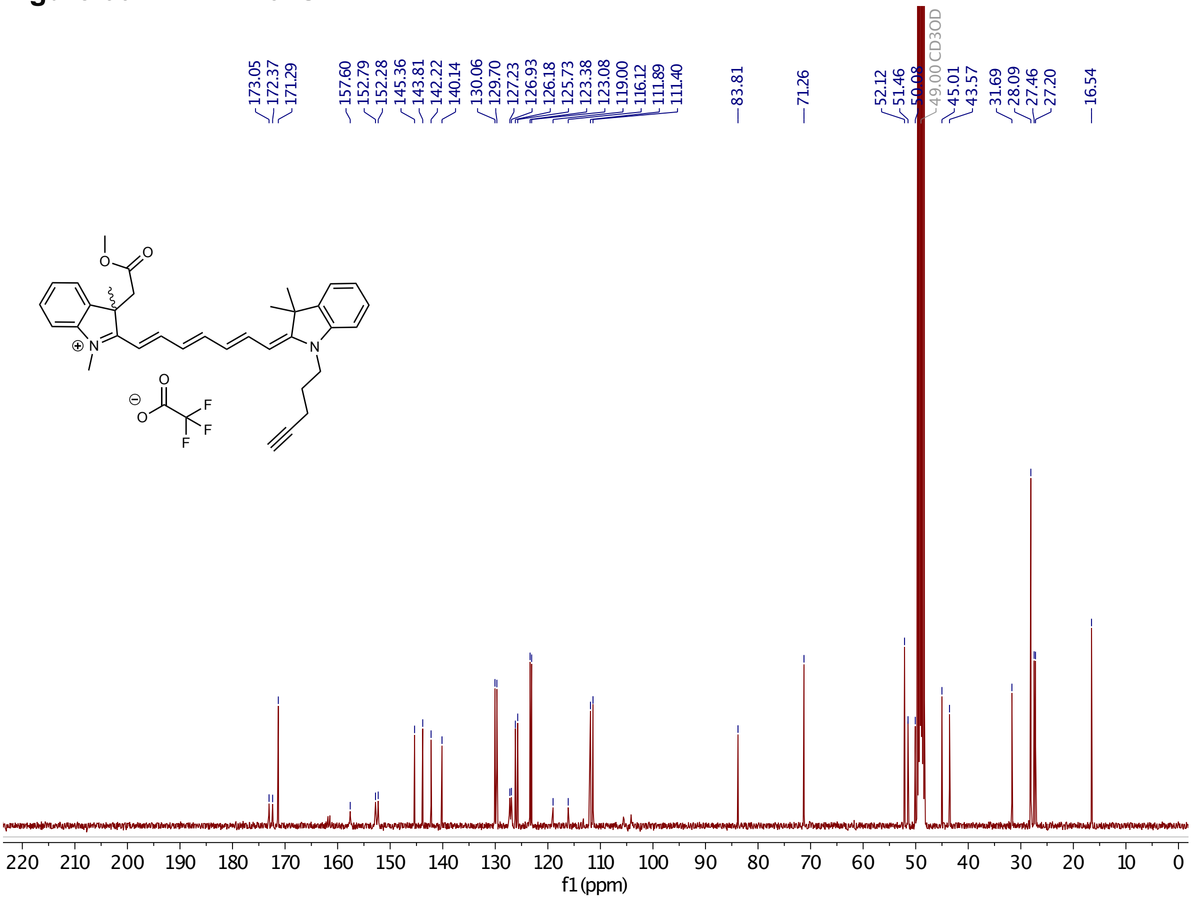

Figure 61. ^{13}C-NMR of **S4**

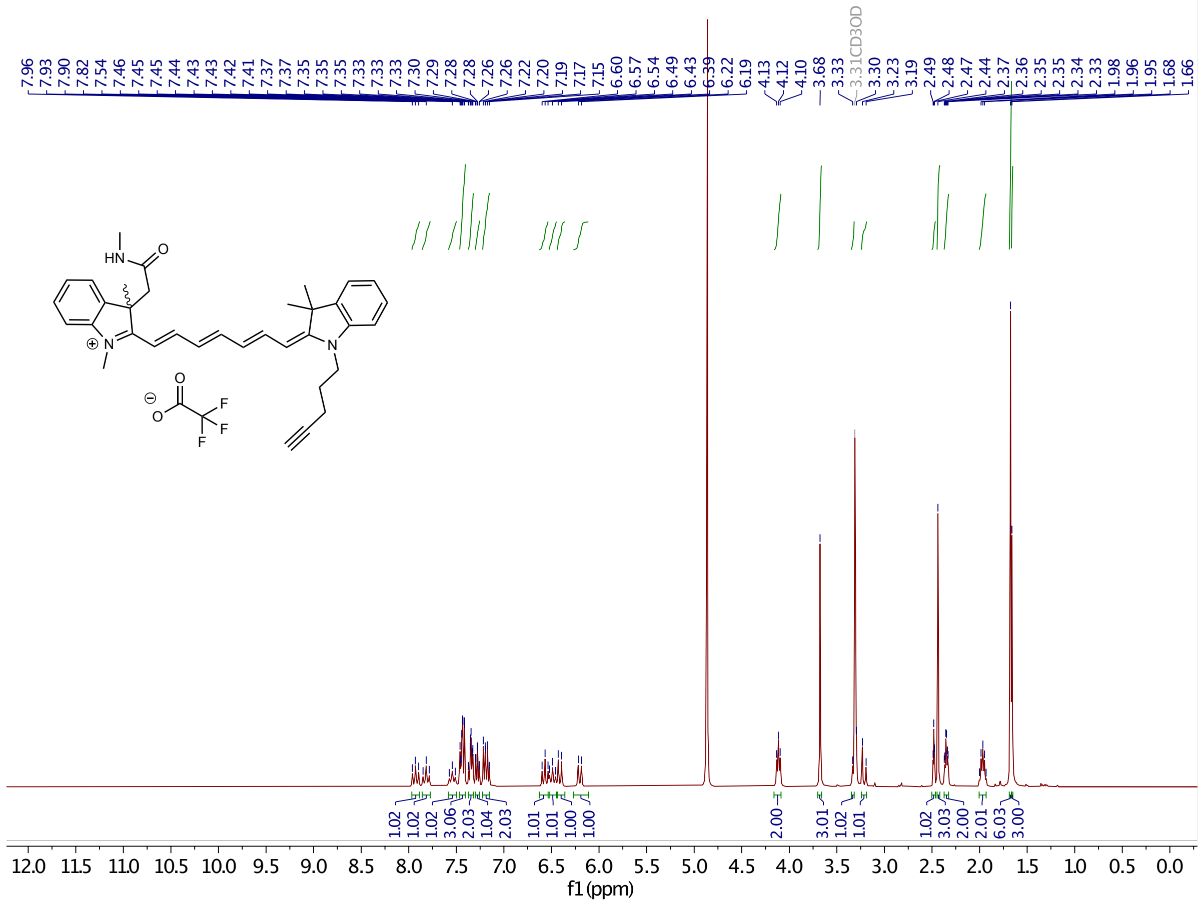

Figure 62. ^{1}H-NMR of **S5**.

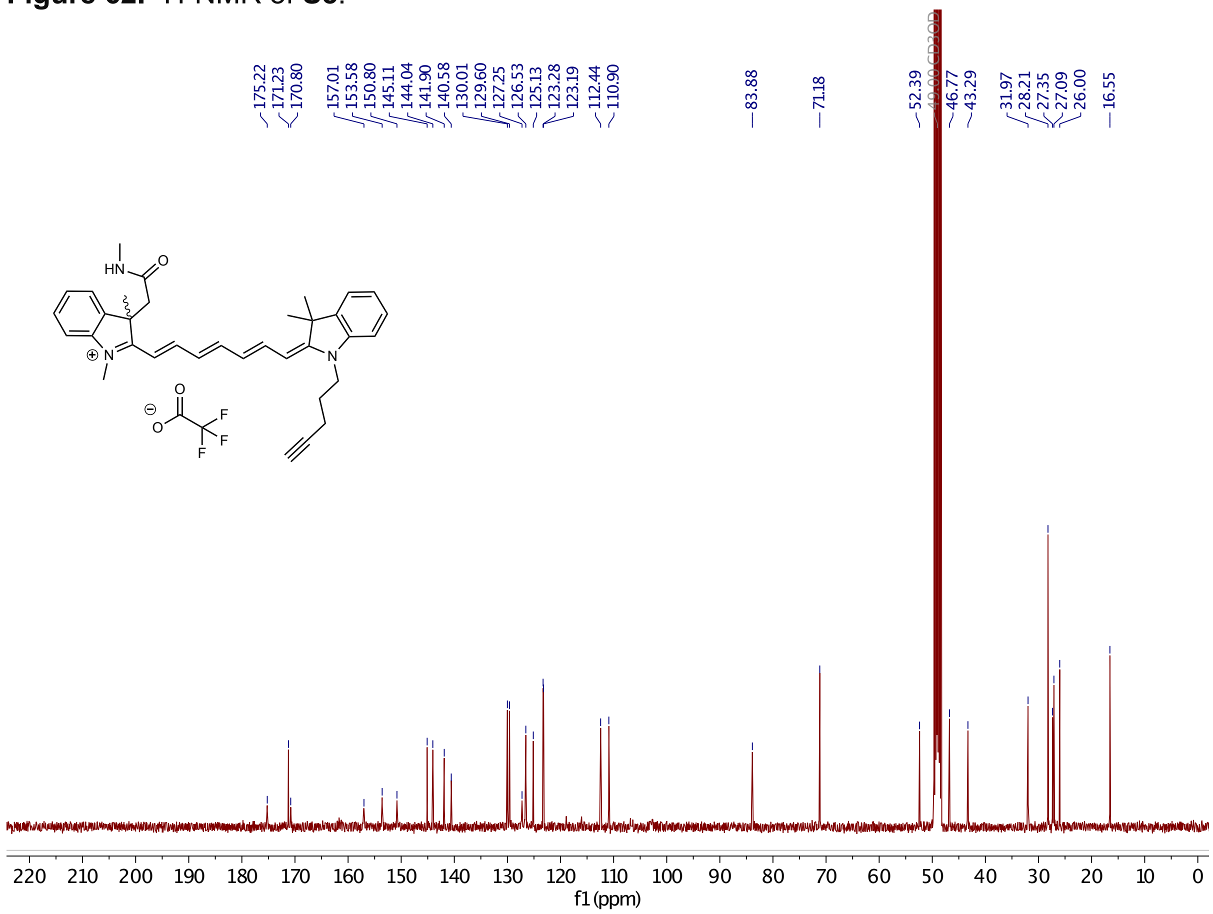

Figure 63. ^{13}C-NMR of **S5**.

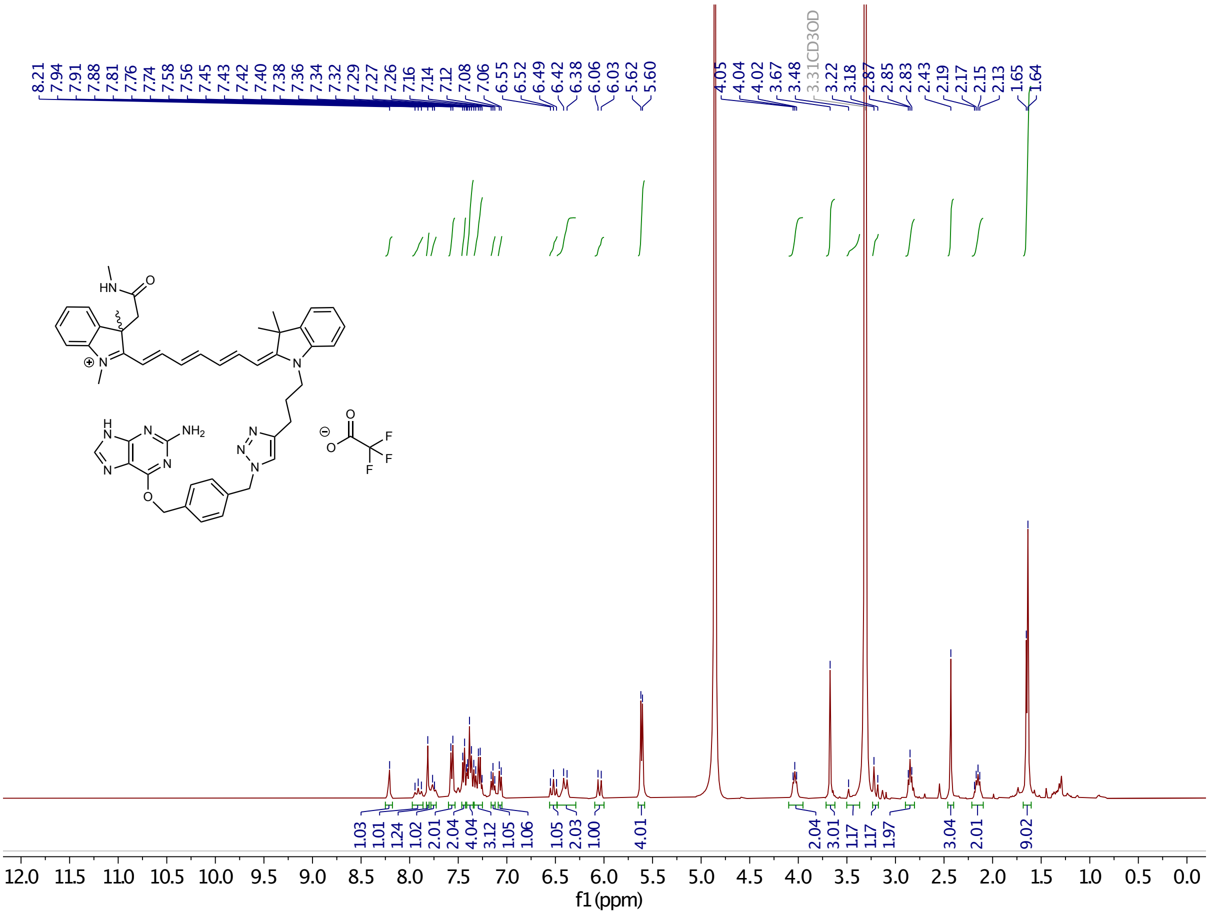

Figure 64. ^{1}H-NMR of **S6**.

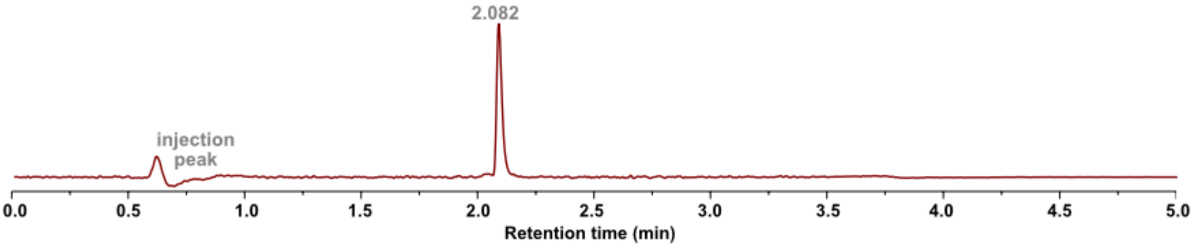

Figure 65. Integrated absorbance signal (190-300 nm) of analytical LC-MS run (10 → 95% CH_3CN in ddH_2O + 0.02% TFA + 0.04% FA over 3 min, then additional 2 min at 95%) of **S6**.

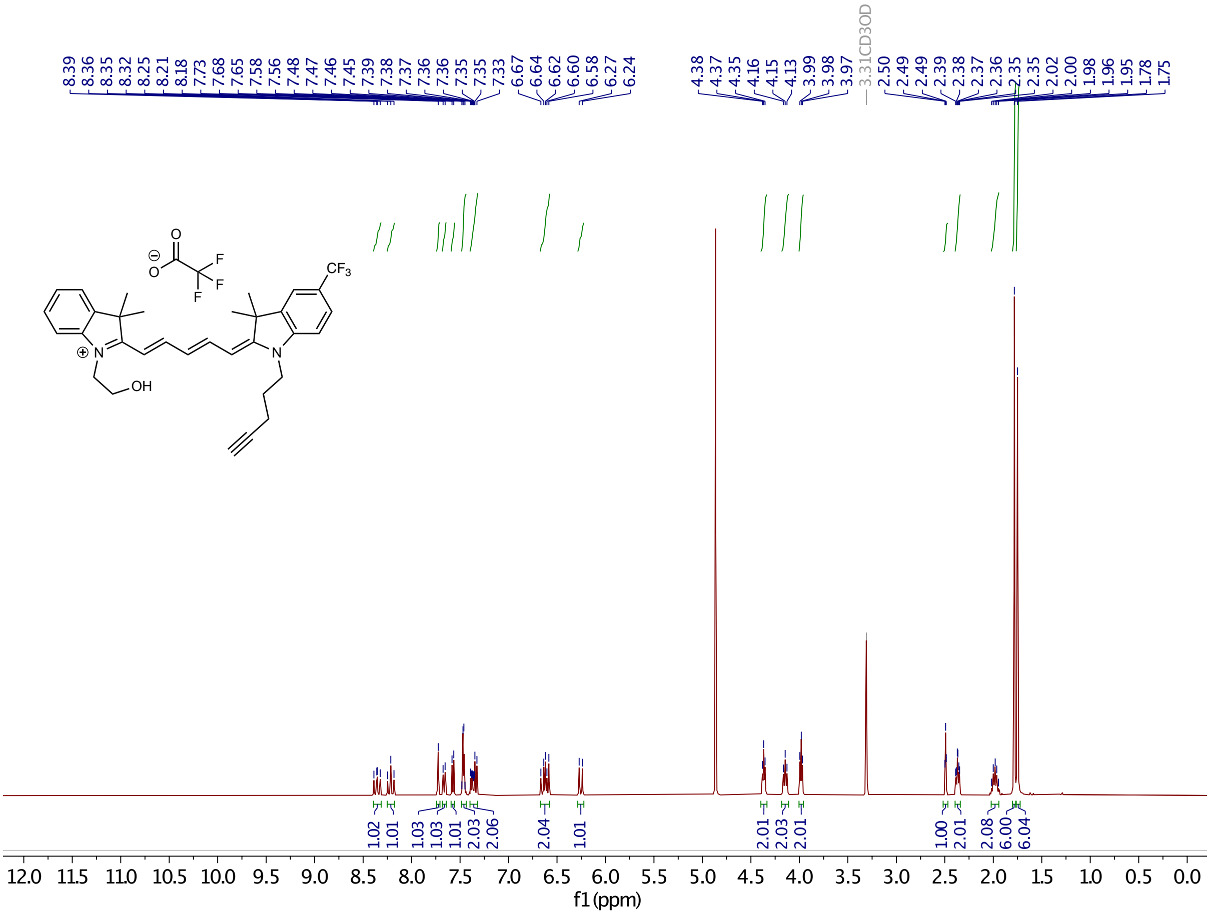

Figure 66. ^{1}H-NMR of **S7**.

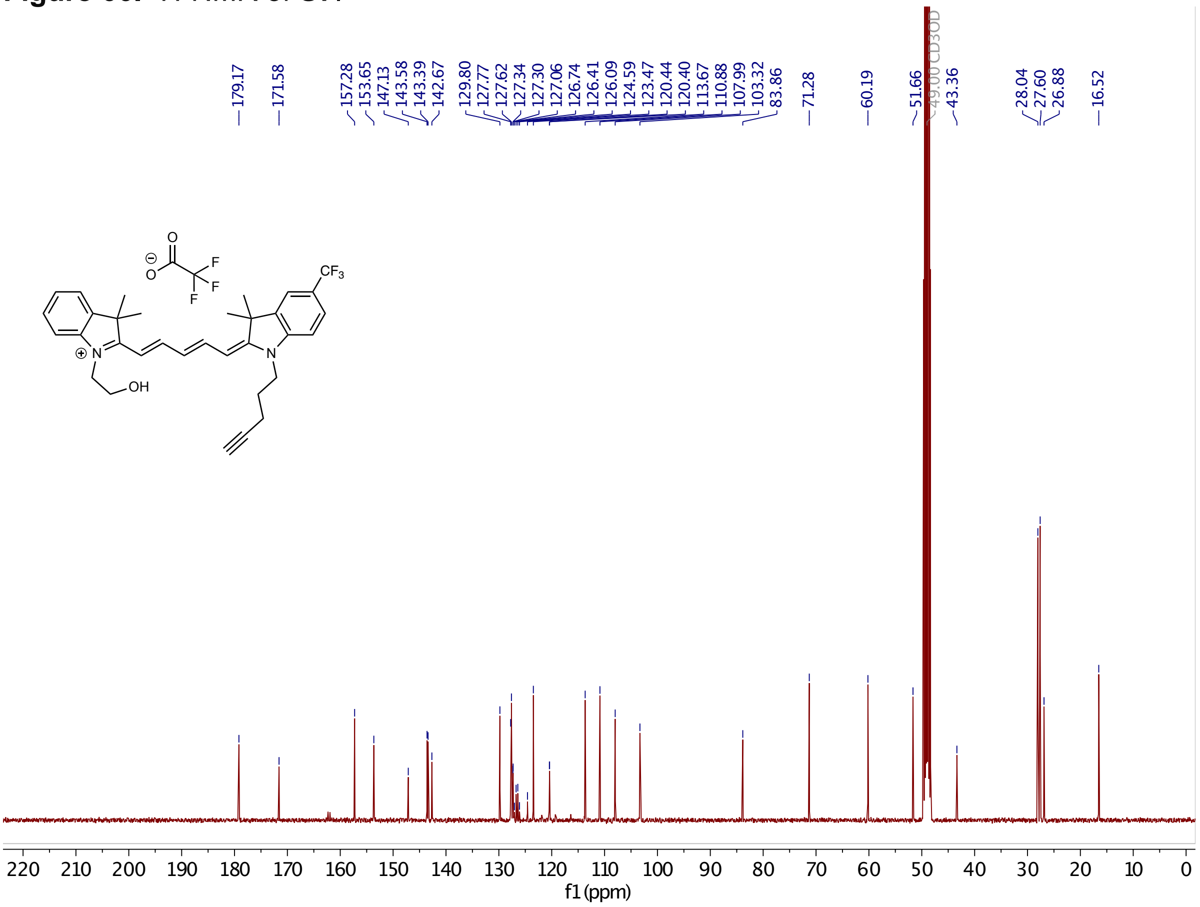

Figure 67. ^{13}C-NMR of **S7**.

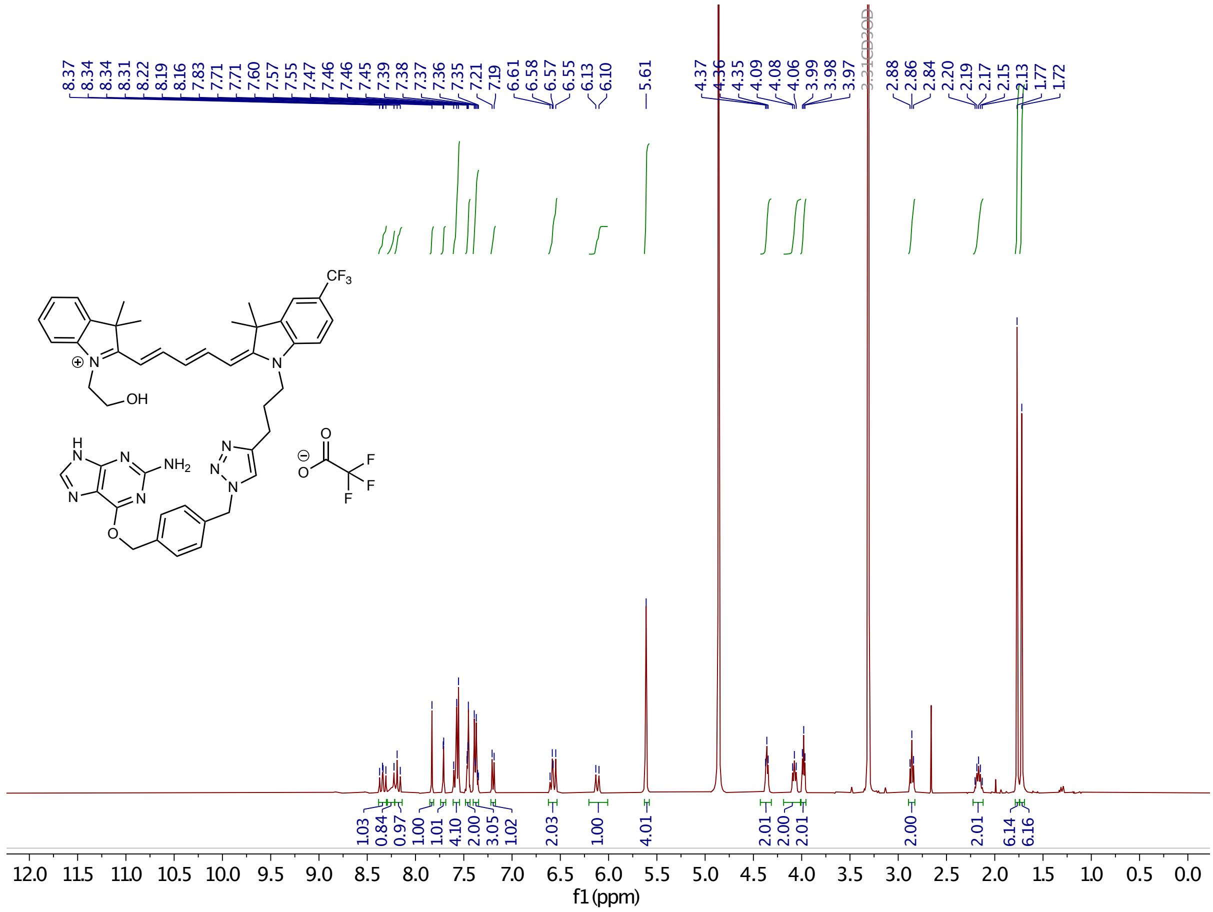

Figure 68. ^{1}H-NMR of **S8**.

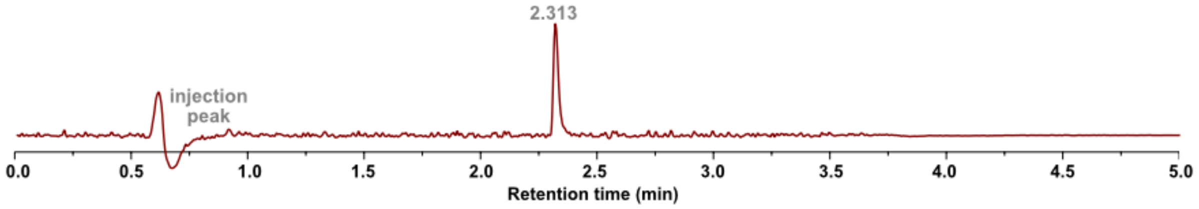

Figure 69. Integrated absorbance signal (190-300 nm) of analytical LC-MS run (10 → 95% CH_3CN in ddH_2O + 0.02% TFA + 0.04% FA over 3 min, then additional 2 min at 95%) of **S8**.

Plasmid maps

H2B-SNAPf-mTurquoise2

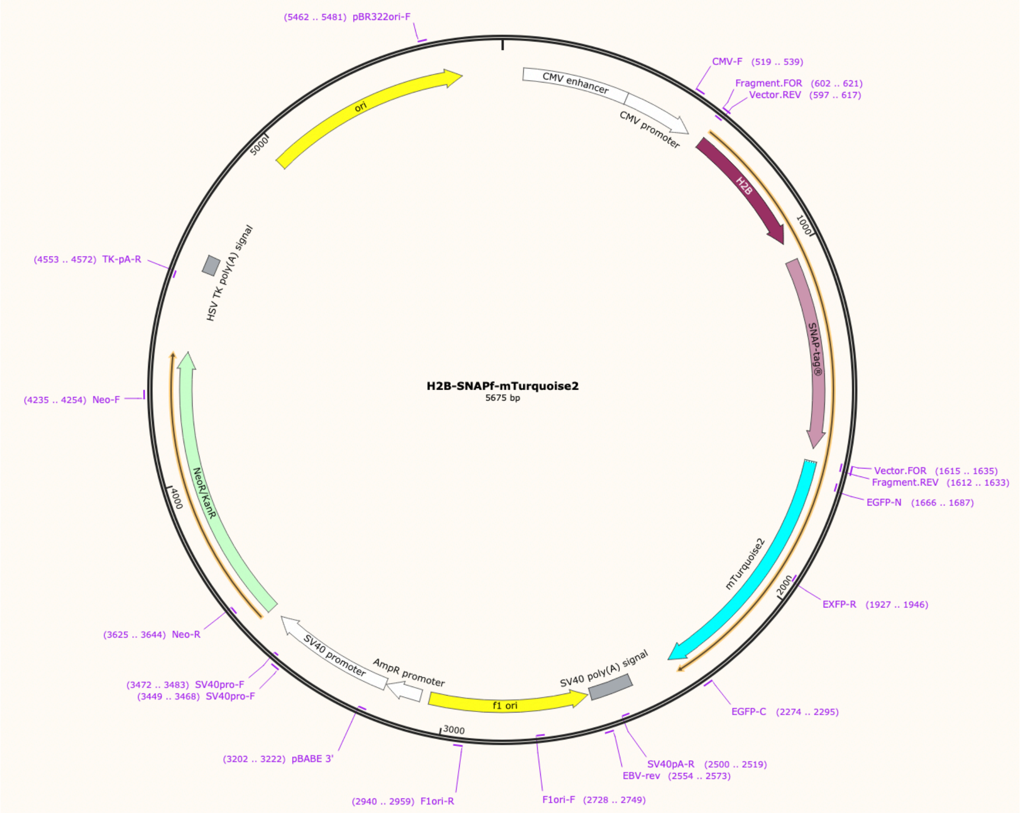

SNAPf-mTurquoise2-KDEL

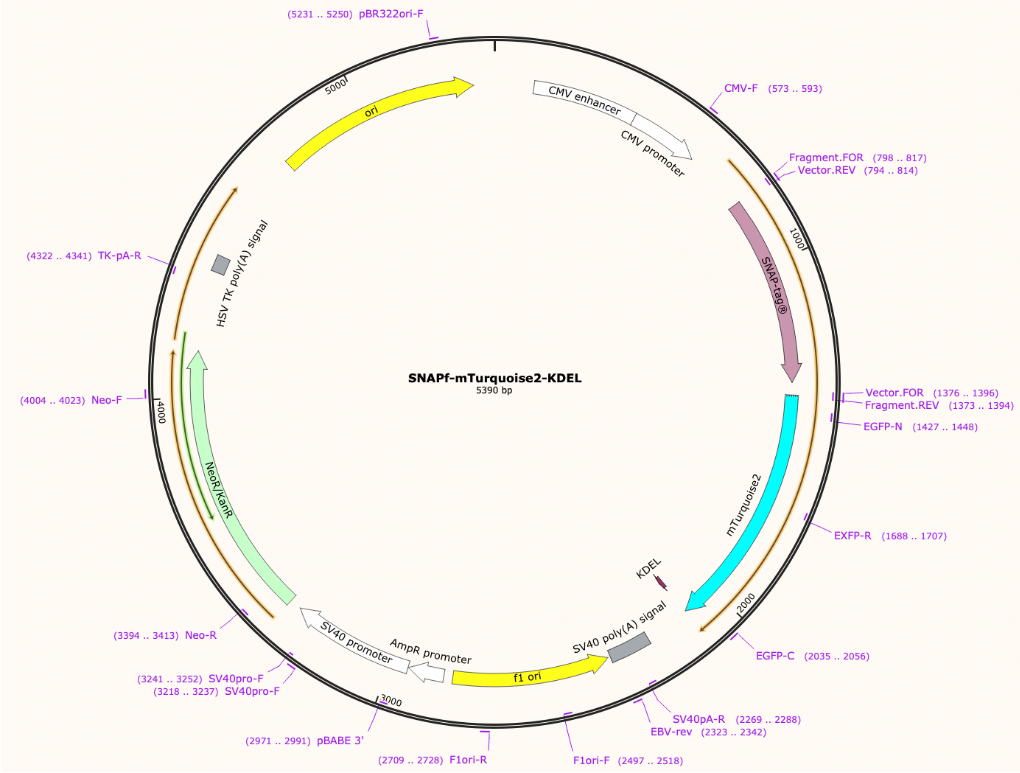

SNAPf-mTurquoise2

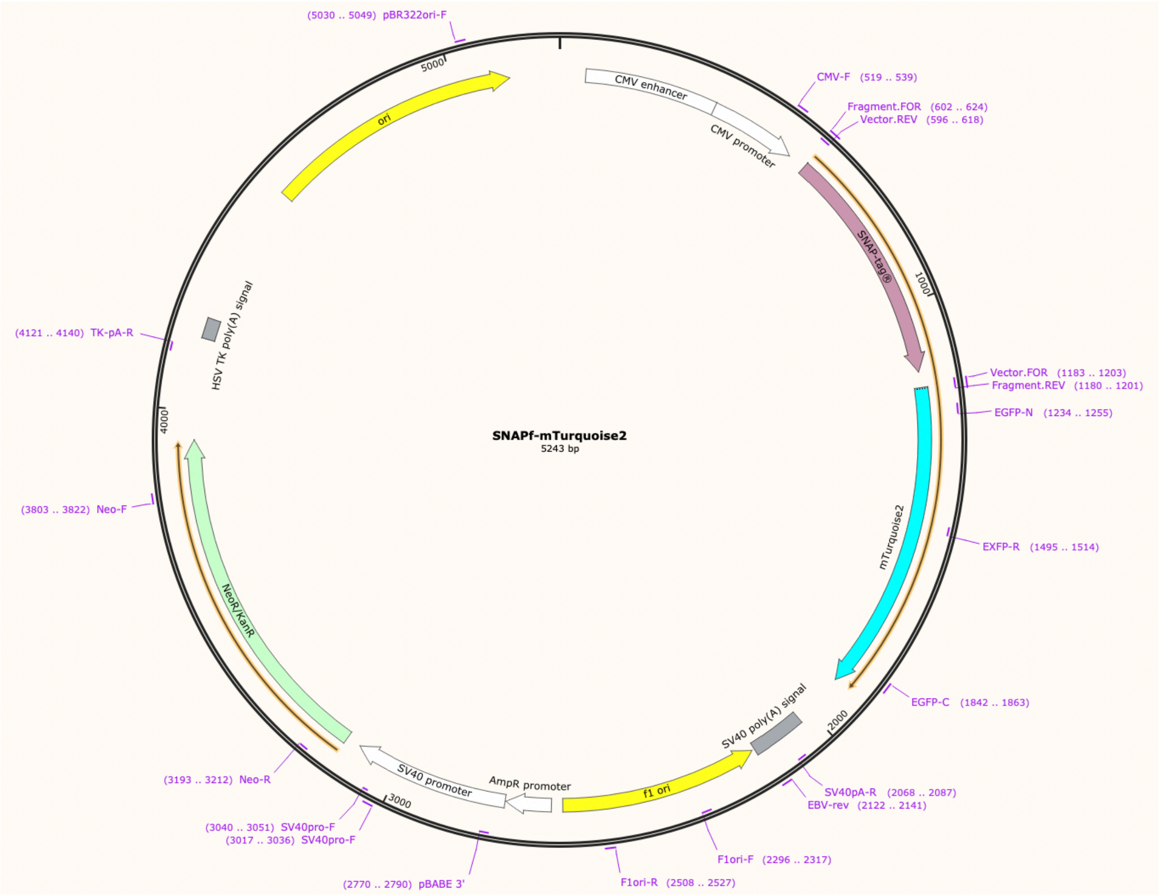

LifeAct-SNAPf-mTurquoise2

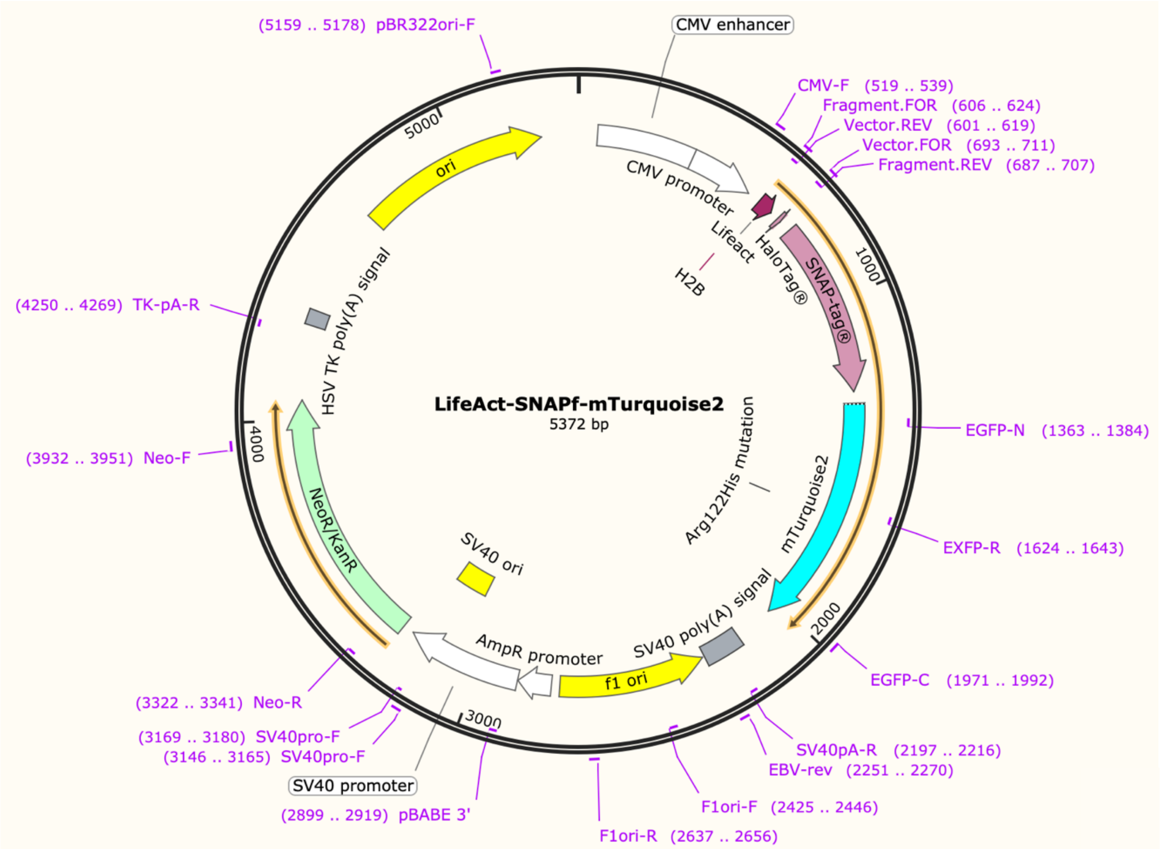

TOM20-SNAPf-mTurquoise2

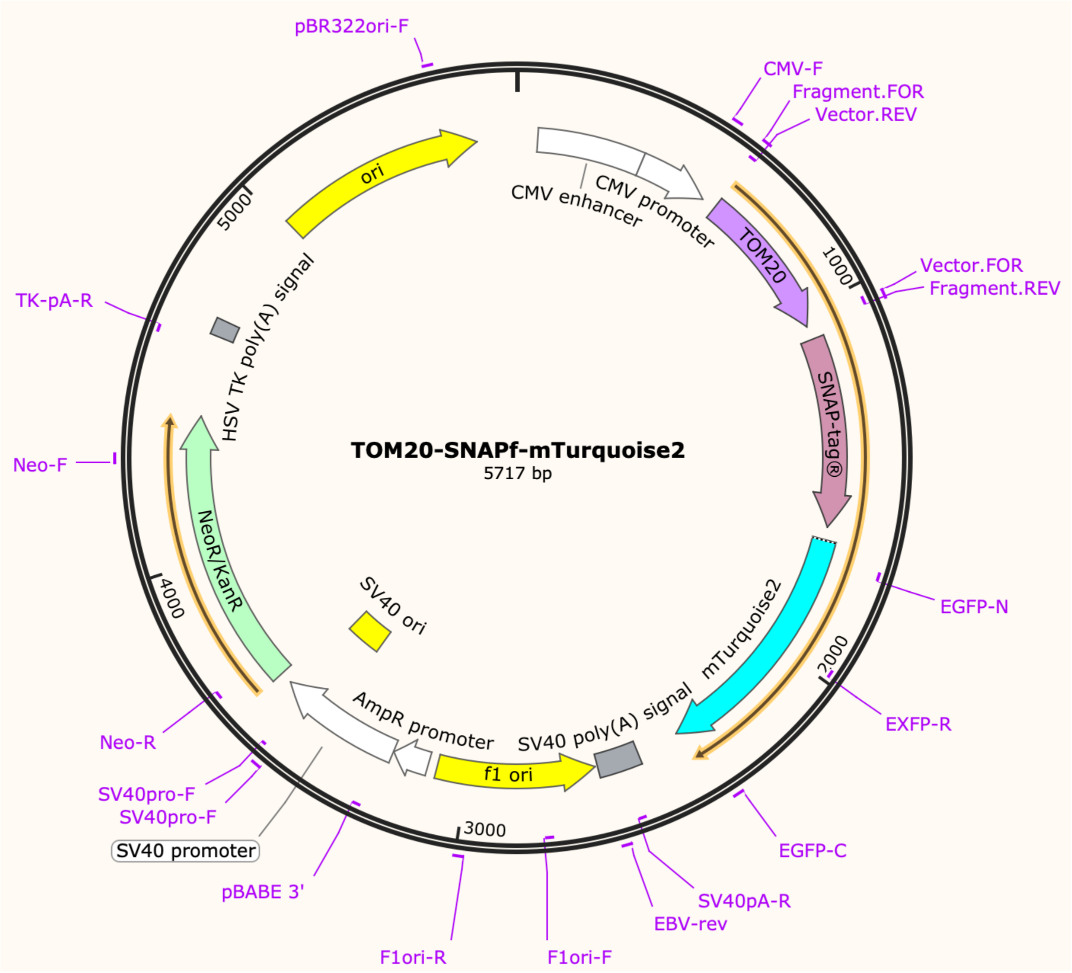

Supplement: Supplementary file 1 — Supplementary Discussions 1 and 2, Figs. 1–69, Tables 1–5, synthetic procedures and plasmid maps. [file 41557_2023_1367_MOESM1_ESM.pdf]
