# Supplementary material for: A general strategy to develop fluorogenic polymethine dyes for bioimaging
Source: Nat Chem. 2023 Nov 27;16(1):28–35. doi: 10.1038/s41557-023-01367-y (PMC10774129; doi:10.1038/s41557-023-01367-y)
Supplement: Supplementary file 2 — Reporting Summary [file 41557_2023_1367_MOESM2_ESM.pdf]

Corresponding author(s): Pablo Rivera-Fuentes

Last updated by author(s): Aug 8, 2023

Reporting Summary

Nature Portfolio wishes to improve the reproducibility of the work that we publish. This form provides structure for consistency and transparency in reporting. For further information on Nature Portfolio policies, see our Editorial Policies and the Editorial Policy Checklist.

Statistics

For all statistical analyses, confirm that the following items are present in the figure legend, table legend, main text, or Methods section.

n/a	Confirmed	
☐	☒	The exact sample size (*n*) for each experimental group/condition, given as a discrete number and unit of measurement
☐	☒	A statement on whether measurements were taken from distinct samples or whether the same sample was measured repeatedly
☒	☐	The statistical test(s) used AND whether they are one- or two-sided<br>*Only common tests should be described solely by name; describe more complex techniques in the Methods section.*
☒	☐	A description of all covariates tested
☐	☒	A description of any assumptions or corrections, such as tests of normality and adjustment for multiple comparisons
☐	☒	A full description of the statistical parameters including central tendency (e.g. means) or other basic estimates (e.g. regression coefficient) AND variation (e.g. standard deviation) or associated estimates of uncertainty (e.g. confidence intervals)
☒	☐	For null hypothesis testing, the test statistic (e.g. *F*, *t*, *r*) with confidence intervals, effect sizes, degrees of freedom and *P* value noted<br>*Give P values as exact values whenever suitable.*
☒	☐	For Bayesian analysis, information on the choice of priors and Markov chain Monte Carlo settings
☒	☐	For hierarchical and complex designs, identification of the appropriate level for tests and full reporting of outcomes
☒	☐	Estimates of effect sizes (e.g. Cohen's *d*, Pearson's *r*), indicating how they were calculated

Our web collection on statistics for biologists contains articles on many of the points above.

Software and code

Policy information about availability of computer code

Data collection	Xcalibur 4.2 (Thermo, mass spectrometry), TopSpin 4.2.0 (Bruker, NMR), Fluoracle (Edinburgh Instruments, fluorescence and absorbance), NIS elements AR (Nikon, microscopy), LAS X (Leica, FLIM), Chirascan 4.2 (Circular dichroism), Spectra Manager CFR (polarimatery), Leica LAS X Navigator (FLIM).
Data analysis	MestreNova 14.2 (mass spectrometry and NMR), Prism 9 (optical spectroscopy), Fiji (confocal and SMLM), LAS X Phasor (Leica, FLIM), ThunderSTORM 1.3 (SMLM)

For manuscripts utilizing custom algorithms or software that are central to the research but not yet described in published literature, software must be made available to editors and reviewers. We strongly encourage code deposition in a community repository (e.g. GitHub). See the Nature Portfolio guidelines for submitting code & software for further information.

Data

Policy information about availability of data

All manuscripts must include a data availability statement. This statement should provide the following information, where applicable:

- Accession codes, unique identifiers, or web links for publicly available datasets
- A description of any restrictions on data availability
- For clinical datasets or third party data, please ensure that the statement adheres to our policy

All data supporting this paper, including coordinates for all calculated structures, are available through Zenodo (DOI: 10.5281/zenodo.7588846). X-ray

crystallographic datasets used for modeling are available from the PDB under accession numbers 6Y8P and 1DNH. Source data are provided with this paper. Samples of small-molecule probes are available from the authors upon reasonable request.

Human research participants

Policy information about studies involving human research participants and Sex and Gender in Research.

Reporting on sex and gender	N/A
Population characteristics	N/A
Recruitment	N/A
Ethics oversight	N/A

Note that full information on the approval of the study protocol must also be provided in the manuscript.

Field-specific reporting

Please select the one below that is the best fit for your research. If you are not sure, read the appropriate sections before making your selection.

☒ Life sciences ☐ Behavioural & social sciences ☐ Ecological, evolutionary & environmental sciences

For a reference copy of the document with all sections, see nature.com/documents/nr-reporting-summary-flat.pdf

Life sciences study design

All studies must disclose on these points even when the disclosure is negative.

Sample size	No sample-size calculation was performed because no experiments in this paper were aimed at describing the properties of a population based on a subsample. In other words, all measurements presented in this paper are discussed independently with no assumption that any specific effect exists or there is a null hypothesis to be rejected. Thus, we do not compare effect sizes or discuss the statistical significance of any observed effects.
Data exclusions	For microscopy studies, dead cells (as judged by cell morphology and excesive blebbing) were excluded from analysis. No other data were excluded.
Replication	Optical spectroscopic measurements (UV and fluorescence) were repeated three times with samples that were prepared from independent batches of the molecule, diluted in independent batches of buffer or solvent, and measured independently. Live-cell experiments were carried out using three different batches of cells (independently cultured from different passage numbers) and measured on different imaging sessions (usually on different days). In all cases, the results of all replicates were virtually identical.
Randomization	No specific randomization strategy was followed. For microscopy studies, cells from different passage numbers were considered different experimental groups. For spectroscopic measurements, separate batches of small molecule or protein were considered independent samples.
Blinding	Blinding was not necessary because all imaging results were obtained using automated computational analysis, thus the researcher cannot affect the results. For all other experiments, group allocation was predetermined by the existence of specific batches of compounds or protein and the experimenter had no influence over the potential variability between groups.

Reporting for specific materials, systems and methods

We require information from authors about some types of materials, experimental systems and methods used in many studies. Here, indicate whether each material, system or method listed is relevant to your study. If you are not sure if a list item applies to your research, read the appropriate section before selecting a response.

Materials & experimental systems

n/a	Involved in the study	
☒	☐	Antibodies
☐	☒	Eukaryotic cell lines
☒	☐	Palaeontology and archaeology
☒	☐	Animals and other organisms
☒	☐	Clinical data
☒	☐	Dual use research of concern

Methods

n/a	Involved in the study	
☒	☐	ChIP-seq
☒	☐	Flow cytometry
☒	☐	MRI-based neuroimaging

Eukaryotic cell lines

Policy information about cell lines and Sex and Gender in Research

Cell line source(s)	HeLa (CLS 300194CP5)
Authentication	Cells were used as received from the distributor (CLS) without further authentication
Mycoplasma contamination	Cells were not tested for mycoplasma contamination
Commonly misidentified lines (See ICLAC register)	No commonly misidentified cell lines were used in this study.
